# Facile and Practical Synthesis of Substituted Piperidine‐2,6‐Diones Under Transition‐Metal Free Condition

**DOI:** 10.1002/open.202500067

**Published:** 2025-06-27

**Authors:** Yue‐Hua Liu, Zhang‐Qin Xue, Kai‐Wen Yang, Hao‐Wen Yin, Bo Yang, Rui Zhang, Hao Zhong, Zhu‐Shuang Bai

**Affiliations:** ^1^ School of Pharmaceutical Sciences and Institute of Materia Medical National Key Laboratory of Advanced Drug Delivery System Key Laboratory for Biotechnology Drugs of National Health Commission (Shandong Academy of Medical Sciences) Key Lab for Rare and Uncommon Diseases of Shandong Province Shandong First Medical University and Shandong Academy of Medical Sciences Jinan 250117, Shandong China; ^2^ Medical Science and Technology Innovation Center (Institute of Translational Medicine) Shandong First Medical University and Shandong Academy of Medical Sciences) Jinan 250117, Shandong China; ^3^ Hubei Key Laboratory of Low Dimensional Optoelectronic Material and Devices Hubei University of Arts and Science, 296 Longzhong Road Xiangyang 441053, Hubei P. R. China; ^4^ Department School of Chemical Engineering and Food Science Hubei University of Arts and Science, 296 Longzhong Road Xiangyang 441053, Hubei P. R. China

**Keywords:** Piperidine-2,6-dione scaffold, Facile and practical synthesis, Basic promoter, Transition-metal free, Michael addition/intramolecular imidation cascade

## Abstract

Substituted piperidine‐2,6‐diones are privileged scaffolds in numerous bioactive molecules and their facile and practical preparation still remains unsolved. In this paper, a facile and practical approach for the construction of α‐substituted and α,α‐/α,β‐disubstituted piperidine‐2,6‐diones from abundant methyl acetates and acrylamides under transition‐metal free condition was disclosed. It features mild reaction condition, operational simplicity, and excellent functional group tolerance, delivering a wide range of piperidine‐2,6‐diones in moderate to good yield. Furthermore, the application potential was further demonstrated by reaction scale‐up (5 kilo‐gram scale) and bio‐active molecule synthesis (Aminoglutethimide and Niraparib). Additional control experiments revealed that the radical process could be excluded from this reaction and a michael addition/intramolecular imidation cascade sequence was proposed based on the control experiments. All these results demonstrated its significant application potential both in academic and industrial production.

## Graphical abstract

KOtBu‐promoted facile and practical synthesis of various substituted piperidine‐2,6‐diones between methyl acetates and acrylamides via a Michael addition/intramolecular imidation cascade sequence has been disclosed. And reaction scale‐up, further transformation of representative products, and preparation of bioactive molecules (e. g. Aminoglutethimide and Niraparib) were achieved in good yield using this newly developed method.

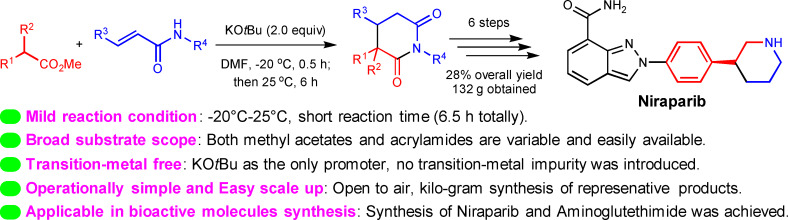

## Introduction

**Figure 1:**
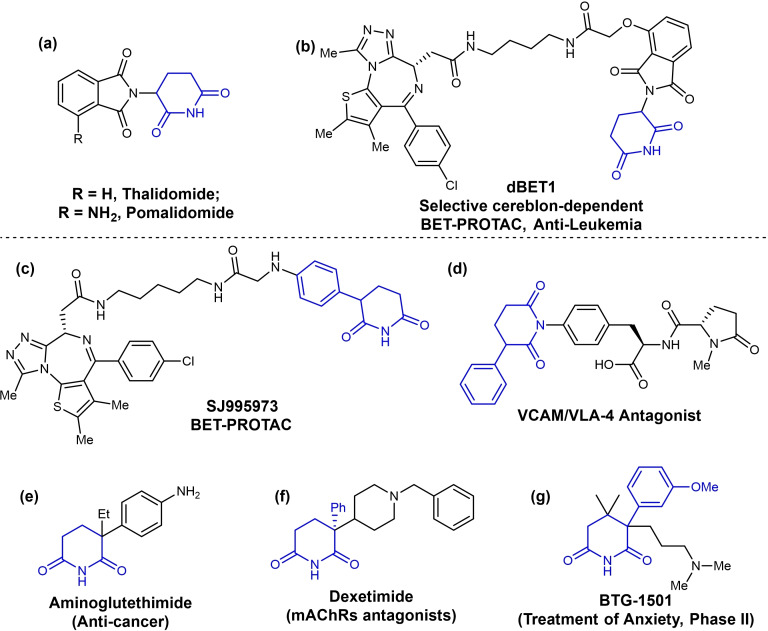
Representative bioactive molecules contained substituted piperidine‐2,6‐diones.

Piperidine‐2,6‐dione motif are found in a variety of bioactive molecules and has gained great interest as promising candidates in various diseases treatment (Figure 1).^[1]^ One of the most famous examples is their application in the field of targeted protein degraders (e. g. proteolysis targeting chimeras, PROTACs), and have been widely applied in clinical cancer treatment, immune disorders, viral infections and neurodegenerative.^[2]^ Traditional PROTAC molecules features C(sp^3^)‐N linked piperidine‐2,6‐dione scaffold based on the chemical structure of Thalidomide and Pomalidomide (Figure 1a), which have proven to be powerful in binding to the E3 ligase/CRBN.^[3]^ dBET1 is a selective cereblon‐dependent BET‐PROTAC, which can delay the progression of Leukemia (Figure 1b).^[4]^ While α‐aryl piperidine‐2,6‐diones features a C(sp^3^)‐C(sp^2^) linkage characteristic, exhibiting a better stability than traditional C(sp3)‐N linked PROTAC molecules and the substituent on the arenes can largely influence the hydrogen acidity at α‐position of piperidine‐2,6‐diones. Therefore, they are identified to be a powerful alternative for traditional PROTACs, acting on the ligase‐binding moiety, with little influence on the binding ability to the E3 ligase cereblon.^[5]^ In 2021, Ranovic and co‐workers developed another α‐aryl piperidine‐2,6‐dione based BET‐PROTAC, namely SJ995973, which can inhibit the viability of human acute myeloid leukemia MV4–11 cells at low picomolar concentration (Figure 1c).^[6]^ Additionally, α‐substituted piperidine‐2,6‐dione motif are also core backbones of various drugs, including VCAM/VLA‐4 antagonists,^[7]^ the aromatase inhibitor Aminoglutethimide for breast cancer (Figure 1e),^[8]^ the mAChRs antagonist Dexetimide for Parkinson's disease,^[9]^ and BTG‐1501, a phase II drug for the treatment of anxiety.^[10]^ Moreover, substituted piperidines, which is widely existed in drugs, could be obtained from the carbonyl reduction of piperidine‐2,6‐diones.^[11]^

Therefore, development of novel approach for the facile construction of substituted piperidine‐2,6‐dione motif is in great demand both in organic synthesis and industrial production.

**Scheme 1:**
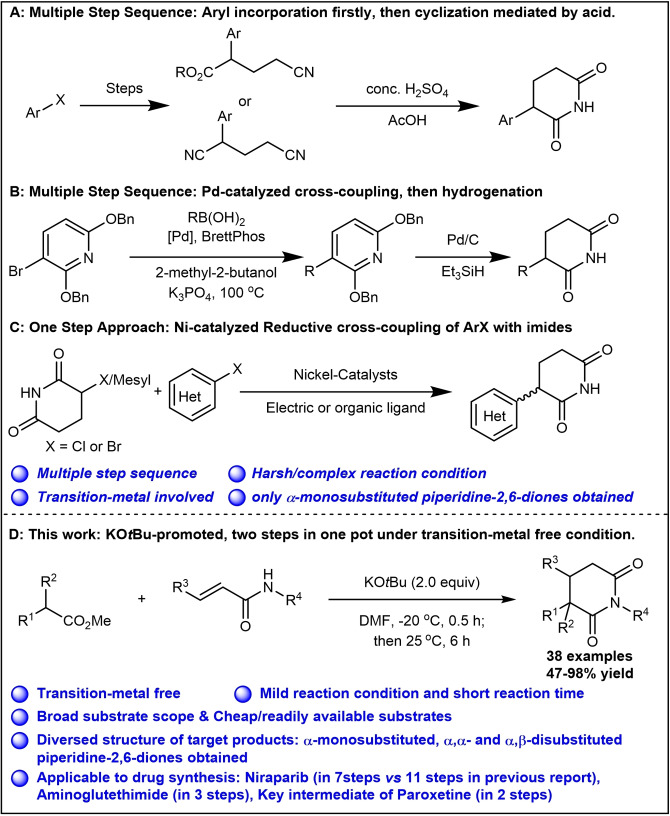
Approaches for the synthesis of piperidine‐2,6‐diones.

To date, synthesis of C3(sp^3^)‐C(sp^2^) featured piperidine‐2,6‐dione motif still remained challenging, with only three distinct strategies being utilized: (1) construction of 2‐aryl methyl 4‐cyano‐butanoate or pentanedinitrile firstly, then cyclization under acidic condition at relative high temperature (Scheme 1A);^[12]^ (2) Pd‐catalyzed cross‐coupling of 2,6‐bis(benzyloxy)pyridine unit with (Het)‐aryl boronic acid/ester, followed by exhaustive catalytic hydrogenation (Scheme 1B);^[6]^ (3) Ni‐catalyzed cross‐coupling of unprotected electrophiles 3‐halo(Cl or Br)/mesyl‐imide with various (hetero)aryl halides with the assistance of electric or various organic ligands(Scheme 1C).^[13]^ Although the above mentioned approaches could deliver the desired C3(sp^3^)‐C(sp^2^) substituted piperidine‐2,6‐diones, they suffer from several drawbacks, including harsh/complex reaction conditions (e. g. high temperature and strong acidic condition),[^6^, ^12^] multiple step sequences,^[12]^ transition‐metal catalyst involved.^[13]^ To be noticed, using the existed approaches, only α‐monosubstituted piperidine‐2,6‐diones could be obtained. For multi‐substituted piperidine‐2,6‐diones, their facile preparation is still challenging and remained unsolved. Therefore, the development of practical protocol with both step and atom economy is highly desirable. In 2022, Rankovic and co‐workers reported one KO*t*Bu‐promoted procedure to prepare α‐phenyl substituted piperidine‐2,6‐diones in THF under 0–50°C. However, the desired products can be only isolated in low to moderate yield (39–46 % yield). Meanwhile, using the existed procedure, only α‐monophenyl substituted piperidine‐2,6‐diones could be delivered.^[5b]^ With the aim to improve the efficiency of this reaction and further broaden its potential application range, herein we report the result for further optimization of this kind of reaction. Using the current reaction condition, both α‐monosubstituted and disubstituted (including α,α‐disubstituted and α,β‐disubstituted) piperidine‐2,6‐diones from readily available substituted methyl acetates and acrylamides can be obtained in good to excellent yield. At the meantime, this reaction can be easily scaled up to 5‐kilogram scales without significant decrease of the yield of desired products. Furthermore, the current procedure has been applied into drug molecule synthesis. All these results demonstrated its great potential both in organic synthesis and drug development.

## Results and Discussion

We initiated this research on the reaction between methyl phenylacetate **1 a** and acrylamide **2 a** (Table 1). By screening various bases (including organic and inorganic, entries 1–5), KO*t*Bu could provide desired product **3 a** in 42 % yield (entry 5), while other bases only give trace to low yield (entries 1–4 *vs* entry 5). Afterwards, the impact of solvent on the reaction efficiency was screened (entries 6–11), DMF was identified to be the most suitable solvent, delivering **3 a** in 58 % yield at room temperature. Poor yield of **3 a** was detected in MeOH (16 %, entry 9) and MeCN (15 %, entry 10), and no desired product was detected when other solvents was applied, including Toluene (entry 6), EtOAc (entry 7), Hexane (entry 8). To our delight, an increased yield of desired product **3 a** was obtained when reaction temperature was decreased to 0 °C (70 %, entry 12) and −20 °C (88 %, entry 13), respectively. Meanwhile, other metal *tert*‐butoxides (entries 14–15) were assessed for their ability to promote this transformation. NaO*t*Bu (entry 14) yielded **3 a** in a slightly lower yield (86 %) compared to KO*t*Bu (88 %, entry 13), while LiO*t*Bu (entry 15) produced **3 a** only in 66 % yield. Finally, the reaction under solvent‐free condition was also evaluated, furnishing **3 a** in 75 % yield. Therefore, the optimized reaction condition was selected as described in entry 13 (**1 a**:**2 a**:KO*t*Bu=2 : 1 : 2, −20 °C for 0.5 h, 25 °C for another 6.0 h in DMF).

**Table 1:** Optimization of the reaction conditions.^[a]^

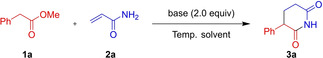				
Entry	Base	Solvent	Temp.(°C)	Yield (%)^[b]^
1	Cs_2_CO_3_	THF	25	13
2	K_2_CO_3_	THF	25	0
3	DBU	THF	25	0
4	KOH	THF	25	23
5	KO*t*Bu	THF	25	42
6	KO*t*Bu	Toluene	25	0
7	KO*t*Bu	EtOAc	25	0
8	KO*t*Bu	Hexane	25	0
9	KO*t*Bu	MeOH	25	16
10	KO*t*Bu	MeCN	25	15
11	KO*t*Bu	DMF	25	58
12^[c]^	KO*t*Bu	DMF	0~25	70
13^[d]^	KO*t*Bu	DMF	−20~25	88
14	NaO*t*Bu	DMF	−20~25	86
15	LiO*t*Bu	DMF	−20~25	66
16^[e]^	KO*t*Bu	–	−20~25	75

[a] Reaction conditions: **1 a** (6 mmol), **2 a** (3 mmol), base (6 mmol), solvent (3 mL), at 25 °C for 6 h. [b] Isolated yield. [c] At 0 °C for 0.5 h, then 25 °C for 6 h. [d] At −20 °C for 0.5 h, then 25 °C for 6 h. [e] **1 a** (15 mmol), **2 a** (3 mmol) without DMF.

**Scheme 2:**
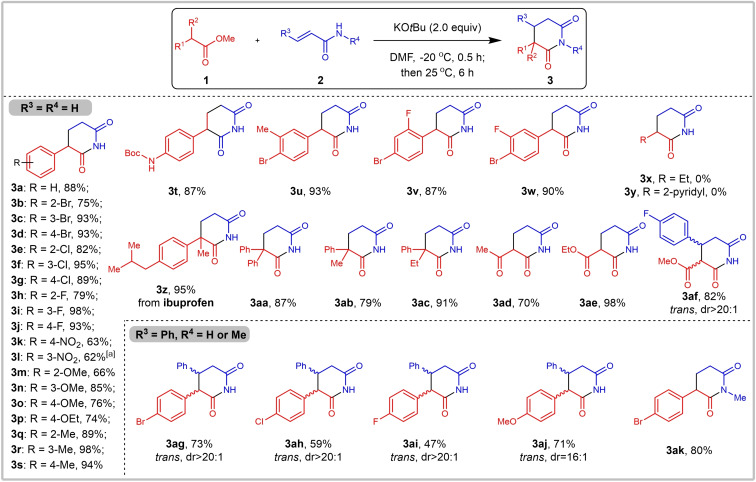
Substrate scope. Reaction conditions: **1** (6 mmol), **2** (3 mmol), KO*t*Bu (6 mmol) in DMF (3 mL), at −20 °C for 0.5 h, then 25 °C for another 6 h. Isolated yields. [a] At −20 °C for 0.5 h, then 50 °C for 6 h

**Scheme 3:**
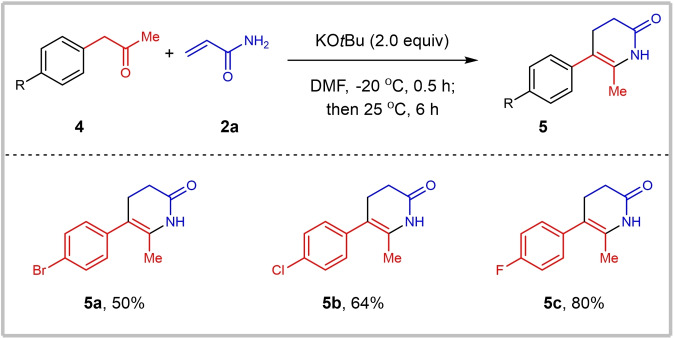
Reactions of α‐aryl ketones **4** with acrylamide **2 a**. Reaction condition: **4** (6 mmol), **2 a** (3 mmol), KO*t*Bu (6 mmol) in DMF (3 mL), at −20 °C for 0.5 h, then 25 °C for another 6 h. Isolated yields.

With a robust set of optimized reaction conditions in hand, substrate scope was further explored as depicted in Scheme 2. Firstly, the reaction of methyl arylacetates **1** bearing various functional groups with acrylamide **2 a** was evaluated. For mono‐substituted substrates, to our delight, both substrates bearing electron‐withdrawing groups (including ‐F, ‐Cl, ‐Br and ‐NO2) or electron‐donating groups (including ‐Me, ‐OMe and ‐OEt) were suitable substrates in this reaction, various α‐aryl piperidine‐2,6‐diones could be obtained in moderate to excellent yields (**3 a**–**3 s**, 62–98 %). Substituents at the *para*‐ and *meta*‐position of the benzene ring are more favorable than those at the *ortho*‐position, as indicated by the yield of the desired products. It's worth noting that NH‐Boc group is also tolerated, the desired product **3 t** was delivered in 87 % yield. For *di*‐substituted benzene ring, the corresponding products **3 u**–**3 w** were obtained in 87 %‐93 % yield successfully. Apart from aryl substituted substrate, methyl butyrate (**1 x**) and methyl 2‐(pyridin‐2‐yl)acetate (**1 y**), were also evaluated, unfortunately, desired products 3x and 3y cannot be delivered under standard reaction condition, indicating **1 x** and **1 y** are not suitable substrates. In previous report, only α‐monosubstituted aryl piperidine‐2,6‐diones could be obtained, di‐ or multi‐substituted piperidine‐2,6‐diones could not be furnished. To be noticed, using our optimized reaction condition, steric hindered α,α‐disubstituted piperidine‐2,6‐diones. The corresponding products (**3 z**–**3 ac**) were delivered in good yield (79–95 %). Notably, when ethyl acetoacetate (**1 ad**), diethyl malonate (**1 ae**) or dimethyl malonate (**1 af**) was employed as substrate, desired 3‐acyl (**3 ad**) or 3‐ester (**3 ae** and *trans*‐**3 af**) substituted piperidine‐2,6‐diones could be furnished in 70–98 % yield. Afterwards, the scope of acrylamides was also explored. As reported, methyl cinnamates or cinnamamides are not effective Michael acceptors, particularly for aryl Michael donors. KO*t*Bu‐promoted Michael addition reactions have primarily focused on oxygen/nitrogen centered nucleophile^[14]^ or acidic hydrogen atom contained carbon‐centered nucleophiles^[15]^ as donors. As for unactivated Michael donors, desired 1,4‐addition products of carbon‐centered Michael donors were obtained via transition‐metal catalyzed coupling reactions.^[16]^ Surprisingly, cinnamamide **2 b** can be transformed into α,β‐diaryl substituted products **3 ag**‐**3 aj** in moderate to good yield (47–73 %) under our standard reaction condition and the dr value was determined by the analysis of original NMR data. Meanwhile, *N*‐Methyacrylamide **2 c** was also tolerated in this reaction, *N*‐methylated α‐aryl piperidine‐2,6‐dione **3 ak** was obtained in 80 % yield. To further enhance the utility of this protocol, reactions of α‐aryl ketones **4** with acrylamide **2 a** were examined (Scheme 3). This Michael addition/intramolecular imidation sequence proceed smoothly, affording 5‐aryl‐6‐methyl‐3,4‐dihydropyridin‐2(1*H*)‐ones (**5 a**‐**5 c**) in moderate to good yield (50–80 %). All these results demonstrate significant application potential of this protocol in organic chemistry and industries.

**Scheme 4:**
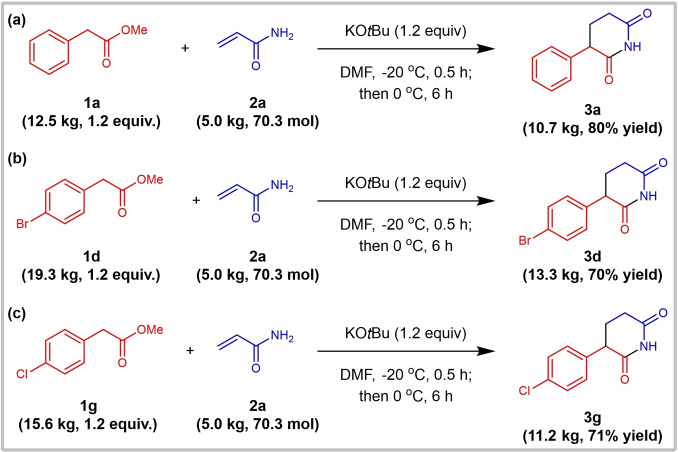
Scheme caption Synthesis of representative products (**3 a**, **3 d** and **3 g**) in kilogram scale

From the perspective of potential practical applications, the scalability of a protocol is a pivotal factor for industrial use. Consequently, the production of representative products was carried out on a kilogram scale (Scheme 4). To reduce costs, the equivalent ratio of **2 a**:**1**:KO*t*Bu was adjusted to 1 : 1.2 : 1.2. Surprisingly, the corresponding products (**3 a**, **3 d** and **3 g**) could be easily obtained through neutralization with 6 N HCl, followed by filtration and recrystallization using DMF/Ethanol (V/V, 1 : 1) in good yield (80 %, 70 % and 71 % yield, respectively).

**Scheme 5:**
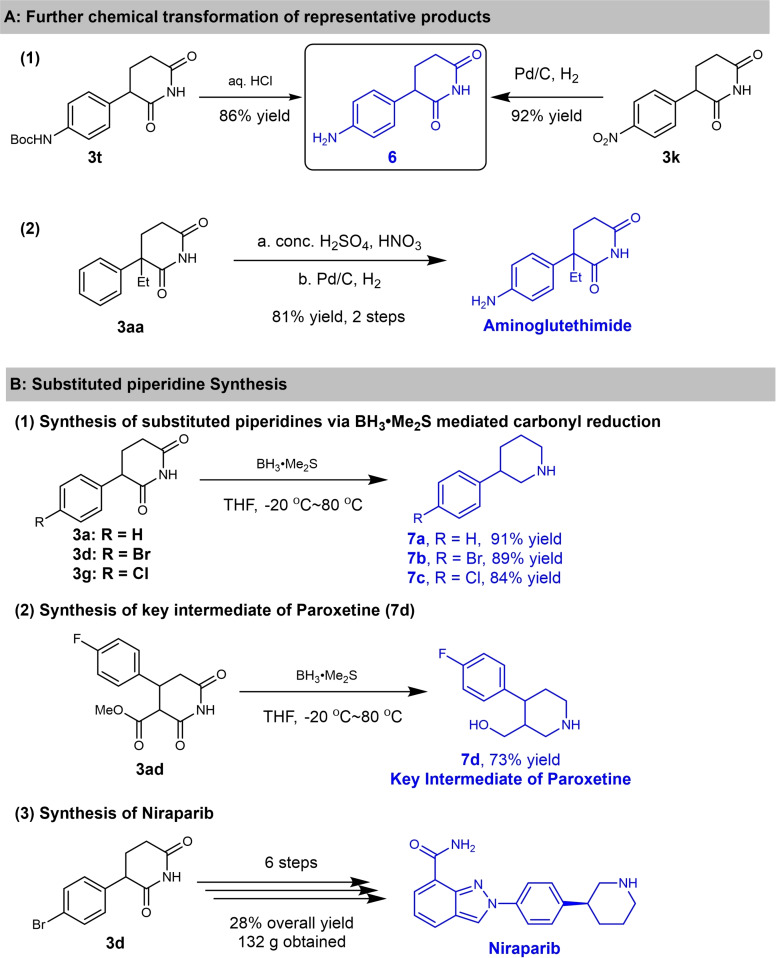
Reaction Application: (A) Further chemical transformation of representative products: (1) 3‐(4‐aminophenyl)piperidine‐2,6‐dione synthesis from **3 t** and **3 k**; (2) Synthesis of Aminoglutethimide; (B) Substituted piperidine synthesis via BH_3_⋅Me_2_S mediated carbonyl reduction: (1) 3‐aryl piperidine synthesis (**7 a**‐**7 c**); (2) Synthesis of key intermediate of Paroxetine (**7 d**); (3) Synthesis of Niraparib.

**Scheme 6:**
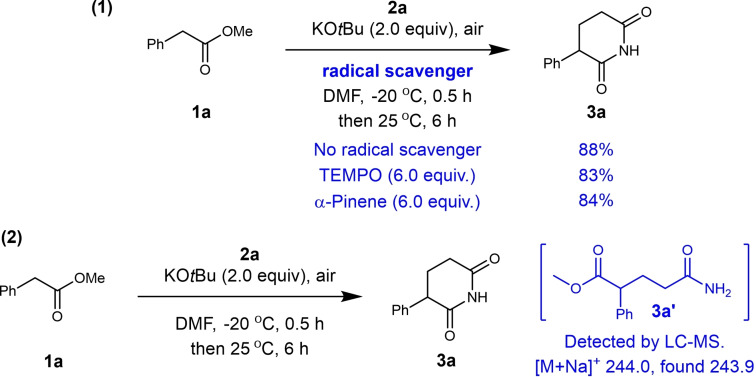
Control experiments: (1) Radical capture by different radical scavenger; (2) Detection of possible intermediate generated during the reaction process. Condition: **1a** (6 mmol), 2a (3 mmol), KOtBu (2.0 equiv.), radical scavenger (12.0 mmol) in DMF (3.0 ml) under air, at ‐25 ^o^C for 0.5 h, then 25 ^o^C for another 6 h. Isolated yield.

**Figure 2:**
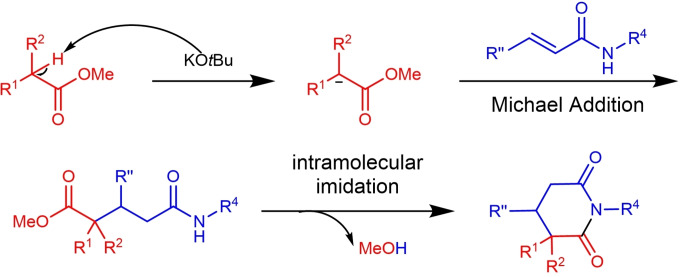
Proposed reaction pathway of KO*t*Bu promoted piperidine‐2,6‐dione formation reaction.

To further demonstrate the potential applications of this protocol, several chemical transformations and the preparation of bioactive molecules were conducted and are outlined in Scheme 5. As reported, 3‐(4‐aminophenyl)piperidine‐2,6‐dione **6** is an effective CRBN ligand for PROTACs. It can be synthesized by Boc‐deprotection of **3 t** under acidic conditions, or through catalytic hydrogenation of **3 k** using Pd/C and H_2_ (Scheme 5A‐1). Anticancer drug Aminoglutethimide can be easily prepared from **3 aa** *via* nitration and reduction process (Scheme 5A‐2). As we all know, the synthesis of substituted piperidines remains an unresolved issue.^[17]^ Therefore, we explored the possibility of synthesizing piperidines through the carbonyl reduction of piperidine‐2,6‐diones using BH_3_⋅Me_2_S. Firstly, α‐aryl piperidines **7 a**‐**7 c** could be obtained from the corresponding starting material (**3 a**, **3 d** and **3 g**) using BH_3_⋅Me_2_S as reductant in good to excellent yield (84–91 % yield, Scheme 5B‐1). Then key intermediate of Paroxetine **7 d** was prepared using **3 ad** as starting material following a similar procedure in 73 % yield (Scheme 5B‐2). Previously, Niraparib, a third generation first‐line drug for ovarian cancer treatment, could be prepared in 11 steps with 11 % overall yield.^[18]^ Using this newly developed protocol, we achieved this goal just in 6 steps on a 100 g scale with 28 % overall yield using **3 d** as key intermediate (Scheme 5B‐3), and detailed synthetic route and procedures are shown in Figure S1 (See Supporting information). These results imply this approach can serve as a general and modular protocol for substituted piperidine synthesis. Subsequently, additional control experiments were carried out to give further insight into the reaction mechanism (as listed in Scheme 6). Firstly, in order to verify whether a radical process was involved in this transformation, radical scavengers (e. g.TEMPO, β‐Pinene) were added into the reaction mixture under standard reaction condition (Scheme 6–1). It's worth noting that the addition of radical scavenger has no significant effect on the yield of 3a (83 %, 84 % vs 88 %). Therefore, the radical process is excluded for this transformation. Afterwards, experiment on the detection of possible reaction intermediate was conducted, possible intermediate **3 a’** could be detected by LC–MS (Figure S2, Supporting information). Based on the above results, a plausible reaction pathway comprising a Michael addition mediated by KO*t*Bu and subsequent intramolecular imidation process was proposed and shown in Figure 2. Firstly, compound **1** was deprotonated by the treatment of KO*t*Bu, then undergoes the Michael addition reaction with compound **2**, furnishing key intermediate **I** smoothly (detected by LC–MS). As previously reported, metal *tert*‐butoxides (metal=K or Na) have been utilized in the direct intermolecular amidation reactions between esters with non‐nucleophilic amines under mild reaction conditions.^[19]^ We envisioned that the intramolecular imidation of primary or N‐Methylated amides with esters could occurred under our standard conditions, deliberating the desired products **3** simultaneously.

## Conclusions

In summary, we have successfully demonstrated a facile and practical approach to generate substituted piperidine‐2,6‐diones from available substituted methyl acetates and acrylamides under transition metal free condition, providing a broad range of functionalized piperidine‐2,6‐diones. Additionally, the method is amenable to scale‐up to kilogram scale and applied into the synthesis of substituted piperidines and bioactive drugs. Additional control experiments revealed a one‐pot Michael addition/intramolecular imidation sequence was involved in this transformation. We believe that this facile and practical synthetic approach will become a versatile and powerful tool for the preparation of substituted piperidine‐2,6‐diones, and further application of this protocol in the design and synthesis of novel PROTAC molecules and scale‐up preparation of drugs are still underway in our laboratory.

## Conflict of Interests

The authors declare no conflict of interest.

## Supporting information

As a service to our authors and readers, this journal provides supporting information supplied by the authors. Such materials are peer reviewed and may be re‐organized for online delivery, but are not copy‐edited or typeset. Technical support issues arising from supporting information (other than missing files) should be addressed to the authors.

Supporting Information

## Data Availability

The data that support the findings of this study are available in the supplementary material of this article.
