# Supplementary material for: Facile and Practical Synthesis of Substituted Piperidine‐2,6‐Diones Under Transition‐Metal Free Condition
Source: ChemistryOpen. 2025 Jun 27;14(11):e202500067. doi: 10.1002/open.202500067 (PMC12598798; doi:10.1002/open.202500067)

ChemistryOpen

Supporting Information

Facile and Practical Synthesis of Substituted Piperidine-2,6-Diones Under Transition-Metal Free Condition

Yue-Hua Liu, Zhang-Qin Xue, Kai-Wen Yang, Hao-Wen Yin, Bo Yang, Rui Zhang,*
Hao Zhong,* and Zhu-Shuang Bai*

Supporting Information

Facile and practical synthesis of substituted piperidine-2,6-diones under transition-metal free condition

Yue-Hua Liu, [‡,a] Zhang-Qin Xue, [‡,a] Kai-Wen Yang,[a] Hao-Wen Yin,[a] Bo Yang,[d] Rui Zhang[c, d,]* Hao Zhong,[b,]* and Zhushuang Bai[a,]*

[a] School of Pharmaceutical Sciences & Institute of Materia Medica, Shandong First Medical University & Shandong Academy of Medical Sciences, National Key Laboratory of Advanced Drug Delivery System, Key Laboratory for Biotechnology Drugs of National Health Commission (Shandong Academy of Medical Sciences), Key Lab for Rare & Uncommon Diseases of Shandong Province, Jinan 250117, Shandong, China. E-mail: baizhushuang@163.com

[b] Medical Science and Technology Innovation Center (Institute of Translational Medicine), Shandong First Medical University & Shandong Academy of Medical Sciences), Jinan 250117, Shandong, China. E-mail: 2411594035@qq.com

[c] Hubei Key Laboratory of Low Dimensional Optoelectronic Material and Devices, Hubei University of Arts and Science, 296 Longzhong Road, Xiangyang 441053, Hubei, P. R. China. E-mail: rzhangccnu@163.com

[d.]School of Chemical Engineering and Food Science, Hubei University of Arts and Science, 296 Longzhong Road, Xiangyang 441053, Hubei, P. R. China.

[‡]Yue-Hua Liu and Zhang-Qin Xue contributed equally to this work.

Table of Contents

1. General information 2
2. General procedure 2
3. Characterization data 3
4. Synthetic procedure for kilogram synthesis of 3a, 3d and 3g 15
5. Further transformations 16
6. Synthesis of Niraparib 18
7. Detection of possible reaction intermediate of 1a with 2a by LC-MS 21
8. Reference 26

9. Spectroscopic data 27

1. General information

^{1}H NMR, ^{13}C NMR data were obtained on Bruker 400MHz Nuclear magnetic resonance NMR spectrometer and AVANCE III Bruker 800 MHz nuclear magnetic resonance spectrometers unless otherwise noted. Chemical shifts (in ppm) were referenced to tetramethylsilane (TMS) (δ = 0.00 ppm) in $CDCl_3$ or dimethyl sulfoxide (δ = 2.50 ppm) in DMSO-d_6 as an internal standard. The data of ^{1}H-NMR was reported as follows: chemical shift, multiplicity (s = singlet, d = doublet, t = triplet, m = multiplet and br = broad), coupling constant (J values) in Hz and integration. ^{13}C-NMR spectra were obtained by the same NMR spectrometers and calibrated with $CDCl_3$ (δ = 77.00 ppm) or DMSO-d_6 (δ = 39.50 ppm). Flash chromatography was performed using 300-400 mesh silica gel with the indicated eluent according to standard techniques. Analytical thin-layer chromatography (TLC) was performed on pre-coated, glass-backed silica gel plates. Analysis of crude reaction mixture was done on an Agilent 7890 GC System with an Agilent 5975 Mass Selective Detector. Visualization of the developed chromatogram was performed by UV absorbance (254 nm) unless otherwise noted. High-resolution mass spectral (HRMS) data were recorded on Bruker APEX IV Fourier transform ion cyclotron resonance mass spectrometer using electrospray ionization (ESI) mode.

2. General procedure

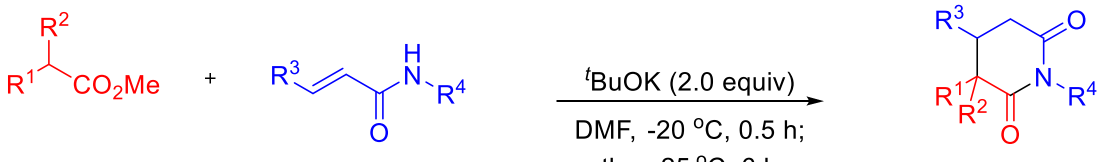

1 **2** **3**

In a 25 ml Schlenk tube, substituted methyl acetates (6 mmol) were mixed with DMF (3.0 ml), and stirred at -20°C for 5 min, then KO*t*Bu (6 mmol) was added into the mixture for 20 min. followed by the addition of acrylamide (3 mmol). The resulting mixture was allowed to stir at 25°C for another 6 h until the complete consumption of **2**, monitored by TLC. Iced water (20 ml) and aq HCl (10 mmol, 6 N) were slowly added into the reaction mixture. The resulting mixture was extracted by ethyl acetate (15 mL* 3), washed with saturated brine (20 ml), dried over Na_2SO_4, filtered and

concentrated under reduced pressure. The residue was purified by column chromatography on silica gel (200-300 mesh) using petroleum ether/ethyl acetate (4/1, v/v) as eluent to give the corresponding products.

3. Characterization data

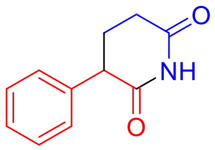

3-Phenylpiperidine-2,6-dione (3a)[1]

The title compound was purified by column chromatography (petroleum ether/ethyl acetate = 2:1) as a white solid in 88% yield. **^{1}H-NMR** (400 MHz, $CDCl_3$) δ 8.48 (s, 1H), 7.37 (dd, J = 8.0, 6.5 Hz, 2H), 7.34 – 7.28 (m, 1H), 7.23 – 7.18 (m, 2H), 3.78 (dd, J = 9.3, 5.4 Hz, 1H), 2.74 – 2.60 (m, 2H), 2.31 – 2.18 (m, 2H).**^{13}C-NMR** (100 MHz, $CDCl_3$) δ 173.4, 172.6, 137.1, 128.9, 128.9, 128.1, 128.1,127.8, 47.9, 30.8, 26.4. **HRMS (ESI):** Calculated for $C_{11}H_{12}NO_2^+$ $[M+H]^+$ 190.0863, found 190.0865.

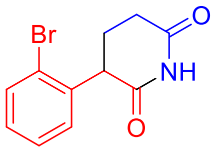

3-(2-Bromophenyl)piperidine-2,6-dione (3b)

The title compound was isolated by column chromatography (petroleum ether/ethyl acetate = 2:1) as a white solid in 75% yield. **^{1}H-NMR** (400 MHz, DMSO-d_6) δ 10.74 (s, 1H), 7.26 (td, J = 8.8, 2.4 Hz, 1H), 7.14 (dd, J = 7.4, 1.6 Hz, 1H), 7.00 (d, J = 8.1 Hz, 1H), 6.91 (t, J = 7.4 Hz, 1H), 3.92 (dd, J = 11.8, 5.2 Hz, 1H), 2.70 – 2.64 (m, 1H), 2.47 (dt, J = 17.0, 3.8 Hz, 1H), 2.20 (ddd, J = 25.0, 12.9, 4.3 Hz, 1H), 1.92 – 1.87 (m, 1H). **^{13}C-NMR** (100 MHz, DMSO-d_6) δ 173.7, 173.6, 139.1, 133.2, 129.6, 129.6, 128.4, 128.4, 48.1, 32.0, 25.2. **HRMS (ESI):** Calculated for $C_{11}H_{11}BrNO_2^+$ $[M+H]^+$ 267.9968, found 267.9970.

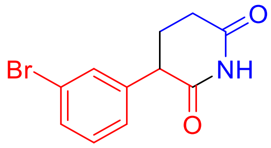

3-(3-Bromophenyl)piperidine-2,6-dione (3c)[2]

The title compound was isolated by column chromatography (petroleum ether/ethyl acetate = 2:1) as a white solid in 93% yield. **^{1}H-NMR** (800 MHz, DMSO-d_6) δ 10.88 (s, 1H), 7.49 – 7.45 (m, 2H), 7.32 – 7.28 (m, 1H), 7.25 (d, J = 7.7 Hz, 1H), 3.90 (dt, J = 14.3, 7.1 Hz, 1H), 2.71 – 2.62 (m, 1H), 2.55 – 2.49 (m, 1H), 2.25 (q, J = 12.7, 4.3 Hz, 1H), 2.06 – 1.98 (m, 1H). **^{13}C-NMR** (200 MHz, DMSO-d_6) δ 174.4, 173.8, 142.4, 132.0, 130.9, 130.3, 128.4, 122.0, 47.5, 32.1, 26.1. **HRMS (ESI):**

Calculated for $C_{11}H_{11}BrNO_2^+$ $[M+H]^+$ 267.9968, found 267.9970.

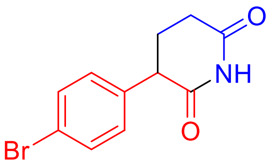

3-(4-Bromophenyl)piperidine-2,6-dione (3d) [3]

The title compound was isolated by column chromatography (petroleum ether/ethyl acetate = 4:1) as a white solid in 93% yield. **^{1}H-NMR** (800 MHz, DMSO-d_6) δ 10.88 (s, 1H), 7.54 (dd, J = 16.3, 8.5 Hz, 2H), 7.21 (t, J = 8.0 Hz, 2H), 3.88 (dd, J = 12.1, 4.9 Hz, 1H), 2.67 (dd, J = 17.5, 12.3, 5.3 Hz, 1H), 2.52 (dt, J = 17.0, 3.7 Hz, 1H), 2.20 (q, J = 12.5, 4.3 Hz, 1H), 2.04 – 1.98 (m, 1H). **^{13}C-NMR** (200 MHz, DMSO-d_6) δ 174.4, 173.8, 139.1, 131.6, 131.6, 131.4, 131.4, 120.6, 47.3, 32.0, 26.1. **HRMS (ESI):** Calculated for $C_{11}H_{11}BrNO_2^+$ $[M+H]^+$ 267.9968, found 267.9971.

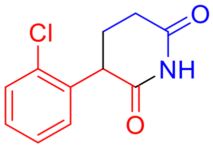

3-(2-Chlorophenyl)piperidine-2,6-dione (3e)

The title compound was isolated by column chromatography (petroleum ether/ethyl acetate = 4:1) as a white solid in 82% yield. **^{1}H-NMR** (800 MHz, DMSO-d_6) δ 10.95 (s, 1H), 7.53 – 7.44 (m, 2H), 7.39 – 7.30 (m, 2H), 4.28 – 4.20 (m, 1H), 2.81 – 2.74 (m, 1H), 2.57 – 2.51 (m, 1H), 2.30 (q, J = 13.0, 4.3 Hz, 1H), 1.99 (dt, J = 13.3, 5.3, 3.0 Hz, 1H). **^{13}C-NMR** (200 MHz, DMSO-d_6) δ 173.8, 173.6, 137.4, 133.8, 131.2, 123.0, 129.3, 127.9, 46.0, 32.0, 24.9. **HRMS (ESI):** Calculated for $C_{11}H_{11}ClNO_2^+$ $[M+H]^+$ 224.0473, found 224.0475.

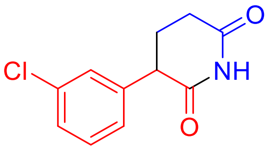

3-(3-Chlorophenyl)piperidine-2,6-dione (3f).

The title compound was isolated by column chromatography (petroleum ether/ethyl acetate = 4:1) as a white solid in 95% yield. **^{1}H-NMR** (800 MHz, DMSO-d_6) δ 10.87 (s, 1H), 7.39 – 7.35 (m, 1H), 7.33 (t, J = 7.8 Hz, 2H), 7.23 – 7.17 (m, 1H), 3.94 – 3.88 (m, 1H), 2.67 (ddd, J = 17.6, 12.5, 5.3 Hz, 1H), 2.55 – 2.49 (m, 1H), 2.25 (qd, J = 12.7, 4.3 Hz, 1H), 2.05 – 1.99 (m, 1H). **^{13}C-NMR** (200 MHz, DMSO-d_6) δ 174.3, 173.8, 142.1, 133.3, 130.6, 129.1, 128.0, 127.4, 47.5, 32.1, 26.1. **HRMS (ESI):** Calculated for $C_{11}H_{11}ClNO_2^+$ $[M+H]^+$ 224.0473, found 224.04751.

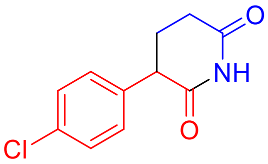

3-(4-Chlorophenyl)piperidine-2,6-dione (3g).

The title compound was isolated by column chromatography (petroleum ether/ethyl acetate = 4:1) as a white solid in 89% yield. **^{1}H-NMR** (800 MHz, DMSO-d_6) δ 10.87 (s, 1H), 7.41 – 7.38 (m, 2H), 7.28 – 7.25 (m, 2H), 3.90 (dd, J = 12.1, 4.9 Hz, 1H), 2.67 (ddd, J = 17.5, 12.3, 5.3 Hz, 1H), 2.52 (dt, J = 16.3, 3.3 Hz, 1H), 2.21 (qd, J = 12.4, 4.4 Hz, 1H), 2.04 – 2.00 (m, 1H). **^{13}C-NMR** (200 MHz, DMSO-d_6) δ 174.5, 173.8, 138.6, 132.1, 131.1, 131.1, 128.7, 128.7,47.2, 32.0, 26.2. **HRMS (ESI):** Calculated for $C_{11}H_{11}ClNO_2^+$ $[M+H]^+$ 224.0473, found 224.0480.

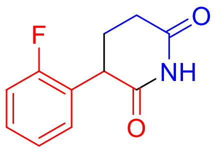

3-(2-Fluorophenyl)piperidine-2,6-dione (3h).

The title compound was isolated by column chromatography (petroleum ether/ethyl acetate = 4:1) as a white solid in 79% yield. **^{1}H-NMR** (800 MHz, DMSO-d_6) δ 10.89 (s, 1H), 7.40 – 7.30 (m, 2H), 7.19 (tt, J = 7.6, 3.8 Hz, 2H), 4.05 (dd, J = 12.7, 5.0 Hz, 1H), 2.82 – 2.68 (m, 1H), 2.57 – 2.45 (m, 1H), 2.21 (qd, J = 13.0, 4.1 Hz, 1H), 2.00 (dtd, J = 13.1, 5.2, 2.9 Hz, 1H). **^{13}C-NMR** (200 MHz, DMSO-d_6) δ 173.8, 173.7, 160.9 (d, J=244Hz), 131.3, 129.6, 126.9, 124.9, 115.9, 43.1, 32.2, 25.2. **^{19}F-NMR** (753 MHz, DMSO) δ -116.5. **HRMS (ESI):** Calculated for $C_{11}H_{11}FNO_2^+$ $[M+H]^+$ 208.0768, found 208.0773.

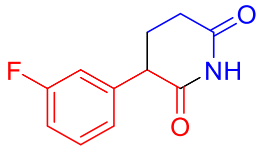

3-(3-Fluorophenyl)piperidine-2,6-dione (3i).

The title compound was isolated by column chromatography (petroleum ether/ethyl acetate = 4:1) as a white solid in 98% yield. **^{1}H-NMR** (800 MHz, DMSO-d_6) δ 10.87 (s, 1H), 7.37 (dt, J = 7.4, 4.3 Hz, 1H), 7.13 – 7.07 (m, 3H), 3.91 (dd, J = 12.1, 4.9 Hz, 1H), 2.67 (ddd, J = 17.5, 12.4, 5.3 Hz, 1H), 2.50 (dd, J = 4.7, 2.9 Hz, 1H), 2.25 (qd, J = 12.5, 4.4 Hz, 1H), 2.03 (ddt, J = 10.2, 8.7, 4.5 Hz, 1H). **^{13}C-NMR** (200 MHz, DMSO-d_6) δ 174.3, 173.8, 162.5 (d, J=242Hz), 142.4, 130.6, 125.4, 116.0, 114.3, 47.6, 32.0, 26.1. **^{19}F-NMR** (753 MHz, DMSO) δ -113.6 (d, J = 6.2 Hz). **HRMS (ESI):** Calculated for $C_{11}H_{11}FNO_2^+$ $[M+H]^+$ 208.0768, found 208.0773.

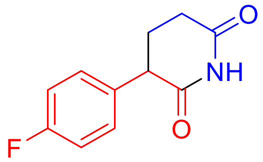

3-(4-Fluorophenyl)piperidine-2,6-dione (3j).

The title compound was isolated by column chromatography (petroleum ether/ethyl acetate = 4:1) as a white solid in 93% yield. **^{1}H-NMR** (800 MHz, DMSO-d_6) δ 10.84 (s, 1H), 7.28 – 7.26 (m, 2H), 7.16 (t, J = 8.4 Hz, 2H), 3.88 (dd, J = 12.0, 4.8 Hz, 1H), 2.67 (ddd, J = 17.4, 12.4, 5.2 Hz, 1H), 2.50 (s, 1H), 2.21 (qd, J = 12.5, 4.1 Hz, 1H), 2.02 (dt, J = 13.1, 4.3 Hz, 1H). **^{13}C-NMR** (200 MHz,

DMSO-d_6) δ 174.67 (s), 173.86 (s), 161.65 (d, J=242Hz), 135.82 (s), 131.06 (s), 131.06 (s), 115.53 (s), 115.53 (s),47.08 (s), 32.03 (s), 26.36 (s). **^{19}F-NMR** (753 MHz, DMSO) δ -116.02 (d, J = 9.4 Hz). **HRMS (ESI):** Calculated for $C_{11}H_{11}FNO_2^+$ [M+H]$^+$ 208.0768, found 208.0773.

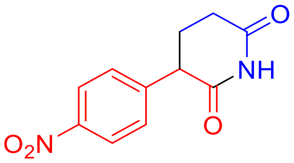

3-(4-Nitrophenyl)piperidine-2,6-dione (3k).[4]

Methyl 2-(4-nitrophenyl)acetate (6 mmol) were placed in dry closed tubes, DMF (3 ml) was added and stirred in a cold trap at -20°C for 5 min, then KO*t*Bu (6 mmol) was added and stirred for 20 min, acrylamide (3 mmol) was added, then the reaction temperature was changed to 50 ℃ and reacted for 6 h. The title compound was isolated by column chromatography (petroleum ether/ethyl acetate = 4:1) as a pale-yellow solid in 63% yield. **^{1}H-NMR** (800 MHz, DMSO-d_6) δ10.97 (s, 1H), 8.22 (dd, J = 16.1, 8.8 Hz, 2H), 7.56 (dd, J = 16.2, 8.7 Hz, 2H), 4.16 – 4.10 (m, 1H), 2.71 (ddd, J = 17.7, 12.7, 5.3 Hz, 1H), 2.56 (dt, J = 17.3, 3.4 Hz, 1H), 2.30 (qd, J = 12.8, 4.3 Hz, 1H), 2.06 (tt, J = 13.0, 8.2, 4.9 Hz, 1H). **^{13}C-NMR** (200 MHz, DMSO-d_6) δ 174.0, 173.7, 147.5, 147.0, 130.7, 130.7, 123.8, 123.8, 47.7, 32.0, 25.9. **HRMS (ESI):** Calculated for $C_{11}H_{11}N_2O_4^+$ [M+H]$^+$ 235.0713, found 235.0741.

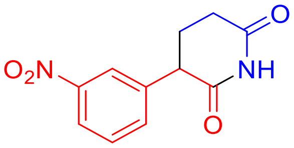

3-(3-Nitrophenyl)piperidine-2,6-dione (3l).[3]

Methyl 2-(3-nitrophenyl)acetate (6 mmol) were placed in dry closed tubes, DMF (3 ml) was added and stirred in a cold trap at -20°C for 5 min, then KO*t*Bu (6 mmol) was added and stirred for 20 min, acrylamide (3 mmol) was added, then the reaction temperature was changed to 50 ℃ and reacted for 6 h. The title compound was isolated by column chromatography (petroleum ether/ethyl acetate = 4:1) as a pale-yellow solid in 62% yield. **^{1}H-NMR** (800 MHz, DMSO-d_6) δ 10.89 (s, 1H), 7.41 – 7.30 (m, 2H), 7.19 (tt, J = 7.6, 3.8 Hz, 2H), 4.05 (dd, J = 12.7, 5.0 Hz, 1H), 2.75 (ddd, J = 18.0, 13.2, 6.6 Hz, 1H), 2.54 (dt, J = 17.2, 3.4 Hz, 1H), 2.21 (qd, J = 13.0, 4.1 Hz, 1H), 2.04 – 1.97 (m, 1H). **^{13}C-NMR** (200 MHz, DMSO-d_6) δ 173.8, 173.7, 161.5, 131.3, 129.7, 126.8, 125.0, 115.9, 43.1, 32.2, 25.2. **HRMS (ESI):** Calculated for $C_{11}H_{11}N_2O_4^+$ [M+H]$^+$ 235.0713, found 235.0726.

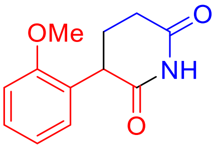

3-(2-Methoxyphenyl)piperidine-2,6-dione (3m).

The title compound was isolated by column chromatography (petroleum ether/ethyl acetate = 4:1)

as a white solid in 66% yield. **^{1}H-NMR** (400 MHz, DMSO-d_6) δ 10.74 (s, 1H), 7.29 – 7.22 (m, 1H), 7.14 (dd, J = 7.4, 1.5 Hz, 1H), 7.01 (d, J = 8.2 Hz, 1H), 6.91 (t, J = 7.4 Hz, 1H), 3.92 (dd, J = 11.8, 5.2 Hz, 1H), 3.74 (s, 3H), 2.72 – 2.63 (m, 1H), 2.49 – 2.43 (m, 1H), 2.20 (ddd, J = 24.9, 12.8, 4.3 Hz, 1H), 1.94 – 1.86 (m, 1H). **^{13}C-NMR** (100 MHz, DMSO-d_6) δ 174.5, 174.0, 157.4, 130.5, 128.9, 128.3, 120.9, 111.9, 56.0, 44.1, 32.0, 25.0. **HRMS (ESI):** Calculated for $C_{12}H_{14}NO_3^+$ [M+H]$^+$ 220.0968, found 220.0974.

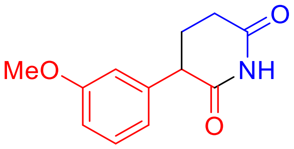

3-(3-Methoxyphenyl)piperidine-2,6-dione (3n).

The title compound was isolated by column chromatography (petroleum ether/ethyl acetate = 4:1) as a white solid in 85% yield. **^{1}H-NMR** (800 MHz, DMSO-d_6) δ 10.83 (s, 1H), 7.24 (t, J = 7.9 Hz, 1H), 6.85 – 6.83 (m, 1H), 6.80 – 6.78 (m, 2H), 3.82 (dd, J = 11.6, 4.9 Hz, 1H), 3.74 (s, 3H), 2.65 (ddd, J = 17.2, 11.9, 5.3 Hz, 1H), 2.48 (dt, J = 17.3, 4.3 Hz, 1H), 2.23 – 2.17 (m, 1H), 2.04 – 2.01 (m, 1H). **^{13}C-NMR** (200 MHz, DMSO-d_6) δ 174.6, 173.9, 159.7, 141.2, 129.8, 121.3, 115.0, 112.7, 55.5, 47.9, 31.8, 26.3. **HRMS (ESI):** Calculated for $C_{12}H_{14}NO_3^+$ [M+H]$^+$ 220.0968, found 220.0974.

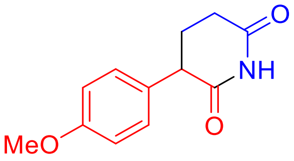

3-(4-Methoxyphenyl)piperidine-2,6-dione (3o).

The title compound was isolated by column chromatography (petroleum ether/ethyl acetate = 4:1) as a white solid in 76% yield. **^{1}H-NMR** (800 MHz, DMSO-d_6) δ 10.81 (s, 1H), 7.13 (d, J = 8.5 Hz, 2H), 6.89 (d, J = 8.6 Hz, 2H), 3.78 (dd, J = 11.5, 4.9 Hz, 1H), 3.73 (s, 3H), 2.64 (ddd, J = 17.2, 11.8, 5.2 Hz, 1H), 2.50 – 2.46 (m, 1H), 2.15 (qd, J = 12.2, 4.3 Hz, 1H), 2.00 (dt, J = 8.9, 4.7 Hz, 1H). **^{13}C-NMR** (200 MHz, DMSO-d_6) δ 175.0, 174.0, 158.6, 131.5, 130.0, 130.0, 114.2, 114.2, 55.5, 47.0, 31.9, 26.5. **HRMS (ESI):** Calculated for $C_{12}H_{14}NO_3^+$ [M+H]$^+$ 220.0968, found 220.0974.

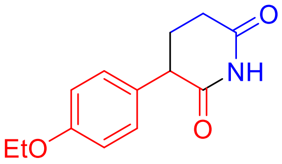

3-(4-Ethoxyphenyl)piperidine-2,6-dione (3p).

The title compound was isolated by column chromatography (petroleum ether/ethyl acetate = 4:1) as a white solid in 74% yield. **^{1}H-NMR** (800 MHz, DMSO-d_6) δ 10.81 (s, 1H), 7.12 (d, J = 8.6 Hz, 2H), 6.88 (d, J = 8.6 Hz, 2H), 4.01 (q, J = 7.0 Hz, 2H), 3.78 (dd, J = 11.5, 4.9 Hz, 1H), 2.65 (td, J = 17.1, 11.7, 5.3 Hz, 1H), 2.48 (dt, J = 17.3, 4.2 Hz, 1H), 2.15 (qd, J = 11.9, 4.4 Hz, 1H), 2.03 – 1.99 (m, 1H), 1.32 (t, J = 7.0 Hz, 3H). **^{13}C-NMR** (200 MHz, DMSO-d_6) δ 175.0, 173.9, 157.9, 131.4,

130.0, 130.0,114.7, 114.7,63.4, 47.0, 31.8, 26.5, 15.2. **HRMS (ESI):** Calculated for $C_{13}H_{16}NO_3^+$ $[M+H]^+$ 234.1125, found 234.1129.

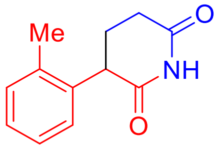

3-(O-tolyl)piperidine-2,6-dione (3q).[5]

The title compound was isolated by column chromatography (petroleum ether/ethyl acetate = 4:1) as a white solid in 89% yield. **^{1}H-NMR** (800 MHz, DMSO-d_6) δ 10.84 (s, 1H), 7.17 (dd, J = 10.2, 4.5 Hz, 1H), 7.16 – 7.12 (m, 2H), 7.09 (dd, J = 12.8, 9.7 Hz, 1H), 4.03 (dd, J = 12.0, 5.0 Hz, 1H), 2.73 (dd, J = 17.5, 12.5, 5.3 Hz, 1H), 2.51 (dt, J = 16.9, 3.6 Hz, 1H), 2.26 (s, 3H), 2.17 (qd, J = 12.6, 4.2 Hz, 1H), 1.97 (ddd, J = 13.4, 8.7, 4.9 Hz, 1H). **^{13}C-NMR** (200 MHz, DMSO-d_6) δ 174.7, 174.0, 138.2, 137.0, 130.7, 128.4, 127.3, 126.4, 44.8, 32.1, 25.5, 19.8. **HRMS (ESI):** Calculated for $C_{12}H_{14}NO_2^+$ $[M+H]^+$ 204.1019, found 204.1024.

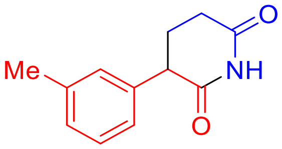

3-(M-tolyl)piperidine-2,6-dione (3r).

The title compound was isolated by column chromatography (petroleum ether/ethyl acetate = 4:1) as a white solid in 98% yield. **^{1}H-NMR** (800 MHz, DMSO-d_6) δ 10.84 (s, 1H), 7.22 (t, J = 7.6 Hz, 1H), 7.08 (d, J = 7.5 Hz, 1H), 7.04 (s, 1H), 7.01 (d, J = 7.7 Hz, 1H), 3.81 (dd, J = 11.4, 5.0 Hz, 1H), 2.66 (dd, J = 17.1, 11.7, 5.3 Hz, 1H), 2.53 – 2.48 (m, 1H), 2.30 (s, 3H), 2.18 (qd, J = 11.9, 4.4 Hz, 1H), 2.05 – 2.01 (m, 1H). **^{13}C-NMR** (200 MHz, DMSO-d_6) δ 174.8, 173.9, 139.6, 137.8, 129.7, 128.7, 128.0, 126.1, 47.8, 31.8, 26.5, 21.5. **HRMS (ESI):** Calculated for $C_{12}H_{14}NO_2^+$ $[M+H]^+$ 204.1019, found 204.1022.

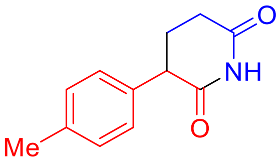

3-(P-tolyl)piperidine-2,6-dione (3s).

The title compound was isolated by column chromatography (petroleum ether/ethyl acetate = 4:1) as a white solid in 94% yield. **^{1}H-NMR** (800 MHz, DMSO-d_6) δ 10.82 (s, 1H), 7.13 (d, J = 7.9 Hz, 2H), 7.10 (d, J = 8.1 Hz, 2H), 3.79 (dd, J = 11.4, 5.0 Hz, 1H), 2.66 – 2.61 (m, 1H), 2.47 (dt, J = 17.3, 4.3 Hz, 1H), 2.28 (s, 3H), 2.15 (qd, J = 11.8, 4.4 Hz, 1H), 2.02 – 1.99 (m, 1H). **^{13}C-NMR** (200 MHz, DMSO-d_6) δ 174.9, 173.9, 136.6, 136.4, 129.4, 129.4,128.9, 128.9, 47.4, 31.8, 26.4, 21.1. **HRMS (ESI):** Calculated for $C_{12}H_{14}NO_2^+$ $[M+H]^+$ 204.1019, found 204.1021.

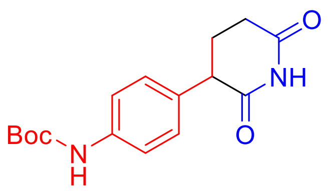

tert-butyl (4-(2,6-dioxopiperidin-3-yl)phenyl)carbamate (3t).

The title compound was isolated by column chromatography (petroleum ether/ethyl acetate = 4:1) as a pale-yellow solid in 87% yield. **^{1}H-NMR** (800 MHz, DMSO-d_6) δ 10.79 (s, 1H), 9.30 (s, 1H), 7.39 (d, J = 7.9 Hz, 2H), 7.09 (d, J = 8.6 Hz, 2H), 3.76 (dd, J = 11.4, 4.9 Hz, 1H), 2.64 (ddd, J = 17.1, 11.7, 5.3 Hz, 1H), 2.49 – 2.45 (m, 1H), 2.14 (ddt, J = 13.0, 11.7, 5.8 Hz, 1H), 2.02 – 1.99 (m, 1H), 1.47 (s, 9H). **^{13}C-NMR** (200 MHz, DMSO-d_6) δ 174.9, 173.9, 153.3, 138.7, 133.1, 129.2, 129.2, 118.6, 118.6, 79.4, 47.2, 31.8, 28.6, 28.6, 28.6, 26.4. **HRMS (ESI):** Calculated for $C_{16}H_{21}N_2O_4^+$ $[M+H]^+$ 305.1496, found 305.1505.

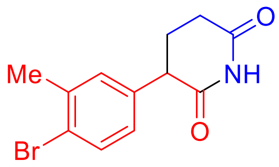

3-(4-Bromo-3-methylphenyl)piperidine-2,6-dione (3u).

The title compound was isolated by column chromatography (petroleum ether/ethyl acetate = 4:1) as a white solid in 93% yield. **^{1}H-NMR** (800 MHz, DMSO-d_6) δ 10.86 (s, 1H), 7.53 (d, 1H), 7.24 (s, 1H), 7.01 (d, 1H), 3.83 (dd, J = 12.0, 4.8 Hz, 1H), 2.67 (ddd, J = 17.4, 12.3, 5.3 Hz, 1H), 2.53 – 2.50 (dt, J = 16.8, 4.0 Hz, 1H), 2.34 (s, 3H), 2.20 (qd, J = 12.5, 4.3 Hz, 1H), 2.04 – 1.99 (m, 1H). **^{13}C-NMR** (200 MHz, DMSO-d_6) δ 174.4, 173.8, 139.3, 137.5, 132.4, 131.9, 128.7, 123.0, 47.3, 32.0, 26.2, 22.9. **HRMS (ESI):** Calculated for $C_{12}H_{13}BrNO_2^+$ $[M+H]^+$ 282.0124, found 282.0131.

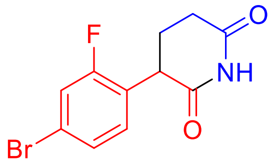

3-(4-Bromo-2-fluorophenyl)piperidine-2,6-dione (3v).

The title compound was isolated by column chromatography (petroleum ether/ethyl acetate = 4:1) as a white solid in 87% yield. **^{1}H-NMR** (400 MHz, DMSO-d_6) δ 10.91 (s, 1H), 7.53 (dd, J = 10.0, 1.9 Hz, 1H), 7.41 (dd, J = 8.3, 1.9 Hz, 1H), 7.31 (t, J = 8.1 Hz, 1H), 4.08 (dd, J = 12.8, 4.9 Hz, 1H), 2.75 (ddd, J = 18.3, 13.2, 5.4 Hz, 1H), 2.55 (ddd, J = 17.2, 4.0, 2.9 Hz, 1H), 2.27 – 2.16 (m, 1H), 2.00 (ddt, J = 13.1, 5.3, 2.7 Hz, 1H). **^{13}C-NMR** (100 MHz, DMSO-d_6) δ 173.7, 173.3, 162.1 (d, J=248Hz), 132.8, 128.09 (s), 126.5, 121.0, 119.4, 42.7, 32.1, 24.8. **^{19}F-NMR** (377 MHz, DMSO) δ -113.3. **HRMS (ESI):** Calculated for $C_{11}H_{10}BrFNO_2^+$ $[M+H]^+$ 285.9874, found 285.9881.

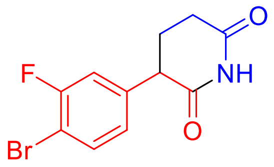

3-(4-Bromo-3-fluorophenyl)piperidine-2,6-dione (3w).

The title compound was isolated by column chromatography (petroleum ether/ethyl acetate = 4:1) as a white solid in 90% yield. **^{1}H-NMR** (400 MHz, DMSO-d_6) δ 10.88 (s, 1H), 7.66 (t, J = 7.9 Hz, 1H), 7.31 (dd, J = 10.2, 1.9 Hz, 1H), 7.07 (dd, J = 8.2, 1.9 Hz, 1H), 3.94 (dd, J = 12.3, 4.9 Hz, 1H), 2.68 (ddd, J = 17.6, 12.5, 5.4 Hz, 1H), 2.57 – 2.49 (m, 1H), 2.26 (qd, J = 12.6, 4.5 Hz, 1H), 2.03 (ddt, J = 10.2, 8.5, 4.4 Hz, 1H). **^{13}C-NMR** (100 MHz, DMSO-d_6) δ 174.0, 173.7, 159.7 (d, *J*=242Hz), 142.0, 133.6, 127.2, 117.7, 106.9, 47.3, 32.0, 25.8. **^{19}F-NMR** (377 MHz, DMSO) δ -108.7. **HRMS (ESI):** Calculated for $C_{11}H_{10}BrFNO_2{}^+$ $[M+H]^+$ 285.9874, found 285.9880.

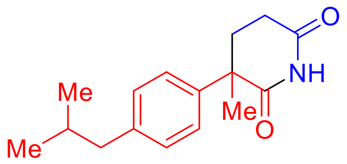

3-(4-Isobutylphenyl)-3-methylpiperidine-2,6-dione (3z).

The title compound was isolated by column chromatography (petroleum ether/ethyl acetate = 4:1) as a white solid in 95% yield. **^{1}H-NMR** (800 MHz, DMSO-d_6) δ 10.92 (s, 1H), 7.19 (d, *J* = 8.1 Hz, 2H), 7.16 (d, *J* = 8.1 Hz, 2H), 3.35 (s, 1H), 2.45 – 2.40 (m, 3H), 2.34 (dt, *J* = 13.5, 4.1 Hz, 1H), 2.09 (td, *J* = 13.1, 4.1 Hz, 1H), 2.02 (ddd, *J* = 17.5, 12.5, 4.8 Hz, 1H), 1.81 (td, *J* = 13.2, 6.5 Hz, 1H), 1.43 (s, 3H), 0.85 (d, *J* = 6.8 Hz, 6H). **^{13}C-NMR** (200 MHz, DMSO-d_6) δ 177.1, 173.5, 140.4, 139.5, 129.8, 129.8,125.9, 125.9,46.8, 44.5, 31.4, 30.0, 29.8, 26.7, 22.6 (d, *J* = 3.7 Hz). **HRMS (ESI):** Calculated for $C_{16}H_{22}NO_2{}^+$ $[M+H]^+$ 260.1645, found 260.1650.

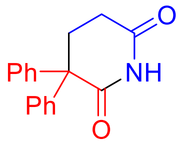

3,3-Diphenylpiperidine-2,6-dione (3aa).[6]

The title compound was isolated by column chromatography (petroleum ether/ethyl acetate = 4:1) as a white solid in 87% yield. **^{1}H-NMR** (800 MHz, DMSO-d_6) δ 11.11 (s, 1H), 7.39 – 7.33 (m, 4H), 7.32 – 7.24 (m, 2H), 7.19 – 7.12 (m, 4H), 2.77 (dd, *J* = 14.4, 8.2 Hz, 2H), 2.33 (dt, *J* = 12.7, 6.3 Hz, 2H). **^{13}C-NMR** (200 MHz, DMSO-d_6) δ 175.3, 173.1, 141.6, 128.8, 128.8, 128.8, 128.8, 128.6, 128.6, 128.6, 128.6, 127.7, 56.5, 30.1 (d, *J* = 10.6 Hz). **HRMS (ESI):** Calculated for $C_{17}H_{16}NO_2{}^+$ $[M+H]^+$ 266.1176, found 266.1179.

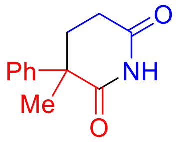

3-Methyl-3-phenylpiperidine-2,6-dione (3ab).[7]

The title compound was isolated by column chromatography (petroleum ether/ethyl acetate = 4:1) as a white solid in 79% yield. **^{1}H-NMR** (800 MHz, DMSO-d_6) δ 10.95 (s, 1H), 7.40 – 7.36 (m, 2H), 7.29 (dd, J = 8.4, 4.8, 1.9 Hz, 3H), 2.45 (dt, J = 17.7, 3.9 Hz, 1H), 2.39 – 2.35 (m, 1H), 2.13 – 2.08 (m, 1H), 2.03 (ddd, J = 17.5, 12.4, 4.9 Hz, 1H), 1.45 (s, 3H). **^{13}C-NMR** (200 MHz, DMSO-d_6) δ 177.0, 173.4, 142.3, 129.3, 129.3, 127.6, 126.2, 126.2, 47.1, 31.3, 29.8, 26.6. **HRMS (ESI):** Calculated for $C_{12}H_{14}NO_2^+$ $[M+H]^+$ 204.1019, found 204.1023.

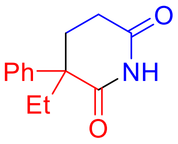

3-Ethyl-3-phenylpiperidine-2,6-dione (3ac).[8]

The title compound was isolated by column chromatography (petroleum ether/ethyl acetate = 4:1) as a white solid in 91% yield. **^{1}H-NMR** (800 MHz, DMSO-d_6) δ 10.89 (s, 1H), 7.40 – 7.37 (m, 2H), 7.32 – 7.28 (m, 3H), 2.47 (dt, J = 17.6, 3.2 Hz, 1H), 2.36 (ddd, J = 14.0, 5.0, 2.8 Hz, 1H), 2.20 – 2.16 (m, 1H), 2.10 (dd, J = 18.1, 13.2, 5.0 Hz, 1H), 1.89 (dt, J = 14.8, 7.3 Hz, 1H), 1.84 – 1.79 (m, 1H), 0.77 (t, J = 7.4 Hz, 3H). **^{13}C-NMR** (200 MHz, DMSO-d_6) δ 176.2, 173.2, 140.3, 129.2, 129.2, 127.6, 126.7, 126.7,50.8, 32.6, 29.6, 26.5, 9.4. **HRMS (ESI):** Calculated for $C_{13}H_{16}NO_2^+$ $[M+H]^+$ 218.1176, found 218.1181.

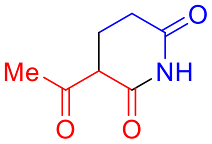

3-Acetylpiperidine-2,6-dione (3ad).[9]

The title compound was isolated by column chromatography (1:2=ethyl acetate/petroleum ether) as a white solid in 70% yield. **^{1}H-NMR** (800 MHz, DMSO-d_6) δ 10.89 (s, 1H), 3.83 (dd, J = 8.8, 5.3 Hz, 1H), 2.48 (d, J = 5.4 Hz, 1H), 2.43 (dd, J = 16.8, 6.3, 4.8 Hz, 1H), 2.27 (s, 3H), 2.14 – 2.09 (m, 1H), 2.02 (s, 1H). **^{13}C-NMR** (200 MHz, DMSO-d_6) δ 205.1, 173.4, 171.6, 95.8, 54.6, 30.1, 20.3. **HRMS (ESI):** Calculated for $C_7H_{10}NO_3^+$ $[M+H]^+$ 156.0655, found 156.0658.

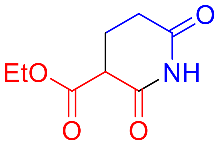

Ethyl-2,6-dioxopiperidine-3-carboxylate (3ae).[10]

The title compound was isolated by column chromatography (petroleum ether/ethyl acetate = 4:1) as a white solid in 98% yield. **^{1}H-NMR** (800 MHz, $CDCl_3$) δ8.63 (s, 1H), 4.29 – 4.24 (m, 2H), 3.60 (dd, J = 7.1, 5.4 Hz, 1H), 2.72 (ddd, J = 17.8, 8.8, 5.1 Hz, 1H), 2.61 (ddd, J = 17.8, 7.5, 5.1 Hz, 1H), 2.36 (dtd, J = 12.6, 7.4, 5.2 Hz, 1H), 2.24 – 2.19 (m, 1H), 1.31 (t, J = 7.2 Hz, 3H). **^{13}C-NMR** (200 MHz, $CDCl_3$) δ 172.0, 168.7, 168.2, 62.3, 48.1, 29.5, 21.4, 14.1. **HRMS (ESI):** Calculated for $C_8H_{12}NO_4^+$ $[M+H]^+$ 186.0761, found 186.0766.

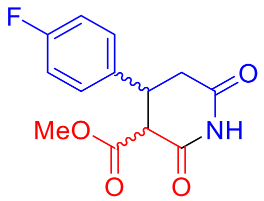

Methyl 4-(4-fluorophenyl)-2,6-dioxopiperidine-3-carboxylate (3af).

The title compound was isolated by column chromatography (petroleum ether/ethyl acetate = 4:1) as a white solid in 82% yield. **^{1}H-NMR** (400 MHz, DMSO-d_6) δ 11.23 (s, 1H), 7.35 (dd, J = 8.7, 5.5 Hz, 1H), 7.17 (t, J = 8.9 Hz, 1H), 4.19 (d, J = 12.5 Hz, 1H), 3.70 – 3.64 (m, 1H), 3.48 (s, 3H), 2.90 (dd, J = 11.2, 5.7 Hz, 1H), 2.60 (dd, J = 16.9, 4.3 Hz, 1H). **^{13}C-NMR** (100 MHz, DMSO-d_6) δ 172.1, 170.3, 169.0, 161.8 (d, J=242Hz), 136.6, 129.7, 129.7, 116.1, 116.1, 55.3, 52.5, 38.8, 38.6. **HRMS (ESI):** Calculated for $C_{13}H_{13}FNO_4^+$ $[M+H]^+$ 266.0823, found 266.0835.

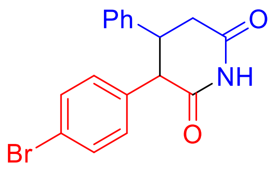

3-(4-Bromophenyl)-4-phenylpiperidine-2,6-dione (3ag).[11]

The title compound was isolated by column chromatography (petroleum ether/ethyl acetate = 4:1) as a white solid in 73% yield(dr＞20:1). **^{1}H-NMR** (800 MHz, DMSO-d_6) δ 11.10 (s, 1H), 7.33 (d, J = 8.0 Hz ,2H), 7.21 – 7.16 (m, 4H), 7.10 – 7.07 (m, 1H), 7.05 (d, J = 8.0 Hz, 2H), 4.31 (d, J = 4.8 Hz, 1H), 3.79 (td, J = 12.7, 4.1 Hz, 1H), 3.03 (dd, J = 16.9, 12.9 Hz, 1H), 2.63 (dd, J = 16.9, 4.1 Hz, 1H). **^{13}C-NMR** (200 MHz, DMSO-d_6) δ 174.3, 172.5, 141.6, 137.7, 132.2, 132.2, 131.2, 131.2, 128.9, 128.9, 127.9, 127.9,127.2, 120.3, 53.4, 42.2, 40.6. **HRMS (ESI):** Calculated for $C_{17}H_{15}BrNO_2^+$ $[M+H]^+$ 344.0281, found 344.0284.

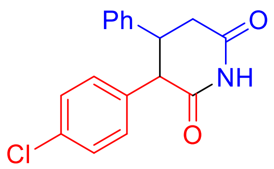

3-(4-Chlorophenyl)-4-phenylpiperidine-2,6-dione (3ah). [11]

The title compound was isolated by column chromatography (petroleum ether/ethyl acetate = 4:1) as a white solid in 59% yield(dr＞20:1). **^{1}H-NMR** (800 MHz, DMSO-d_6) δ 11.07 (s, 1H), 7.37 – 7.30 (m, 1H), 7.18 – 7.15 (m, 4H), 7.11 – 7.09 (m, 2H), 6.96 (t, J = 8.9 Hz, 2H), 4.28 (d, J = 12.3 Hz, 1H), 3.77 (td, J = 12.7, 4.1 Hz, 1H), 3.04 (dd, J = 16.9, 12.9 Hz, 1H), 2.62 (dd, J = 16.8, 4.0 Hz, 1H). **^{13}C-NMR** (200 MHz, DMSO-d_6) δ 174.5, 172.5, 161.9, 160.7, 141.7, 134.4, 131.8, 128.8, 127.9, 127.1, 115.1, 115.0, 53.3, 42.5, 40.6. **HRMS (ESI):** Calculated for $C_{17}H_{15}ClNO_2^+$ $[M+H]^+$ 300.0786, found 300.0792.

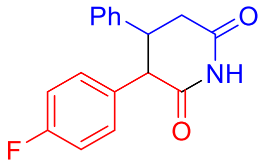

3-(4-Fluorophenyl)-4-phenylpiperidine-2,6-dione (3ai).[12]

The title compound was isolated by column chromatography (petroleum ether/ethyl acetate = 4:1) as a white solid in 47% yield(dr＞20:1). **^{1}H-NMR** (800 MHz, DMSO-d_6) δ 11.07 (s, 1H), 7.20 – 7.16 (m, 4H), 7.13 – 7.07 (m, 3H), 6.97 (t, J = 8.8 Hz, 2H), 4.29 (d, J = 12.3 Hz, 1H), 3.78 (td, J = 12.6, 4.0 Hz, 1H), 3.04 (dd, J = 16.9, 12.9 Hz, 1H), 2.62 (dd, J = 16.9, 4.0 Hz, 1H). **^{13}C-NMR** (200 MHz, DMSO-d_6) δ 174.6, 172.5, 161.3 (d, J=240Hz), 141.7, 134.4, 131.8, 131.8,128.8, 128.8, 127.9, 127.92 (s), 127.1, 115.1, 115.1,53.3, 42.5, 40.6. **^{19}F-NMR** (377 MHz, DMSO) δ -116.3. **HRMS (ESI):** Calculated for $C_{17}H_{15}FNO_2^+$ [M+H]$^+$ 284.1081, found 284.1073.

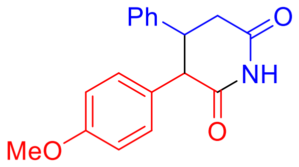

3-(4-Methoxyphenyl)-4-phenylpiperidine-2,6-dione (3aj).

The title compound was isolated by column chromatography (petroleum ether/ethyl acetate = 4:1) as a white solid in 71% yield(dr=16:1). **^{1}H-NMR** (800 MHz, DMSO-d_6) δ 11.00 (s, 1H), 7.19 – 7.16 (m, 4H), 7.09 – 7.06 (m, 1H), 6.98 (d, J = 8.7 Hz, 2H), 6.70 (d, J = 8.7 Hz, 2H), 4.18 (d, J = 12.1 Hz, 1H), 3.73 (dd, J = 8.4, 4.0 Hz, 1H), 3.64 (s, 3H), 3.00 (dd, J = 16.9, 12.7 Hz, 1H), 2.60 (dd, J = 16.8, 4.1 Hz, 1H). **^{13}C-NMR** (200 MHz, DMSO-d_6) δ 174.9, 172.6, 158.2, 142.0, 130.8, 130.1, 130.1,128.8, 128.8, 127.9, 127.9, 127.0, 113.8, 113.8, 55.3, 53.1, 42.5, 40.6. **HRMS (ESI):** Calculated for $C_{18}H_{18}NO_3^+$ [M+H]$^+$ 296.1281, found 296.1292.

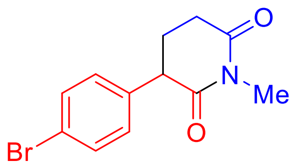

3-(4-Bromophenyl)-1-methylpiperidine-2,6-dione (3ak).

The title compound was isolated by column chromatography (petroleum ether/ethyl acetate = 4:1) as a white solid in 80% yield. **^{1}H-NMR** (800 MHz, DMSO-d_6) δ 7.55 – 7.51 (m, 2H), 7.21 – 7.19 (m, 2H), 3.98 (dd, J = 12.1, 4.9 Hz, 1H), 3.03 (s, 3H), 2.78 (ddd, J = 17.5, 12.3, 5.3 Hz, 1H), 2.66 (dt, J = 17.3, 4.1 Hz, 1H), 2.20 (qd, J = 12.4, 4.4 Hz, 1H), 2.02 – 1.99 (m, 1H). **^{13}C-NMR** (200 MHz, DMSO-d_6) δ 173.9, 172.9, 139.5, 131.6, 131.6, 131.4, 131.4, 120.6, 47.9, 32.3, 26.8, 25.1. **HRMS (ESI):** Calculated for $C_{12}H_{13}BrNO_2^+$ [M+H]$^+$ 282.0124, found 282.0131.

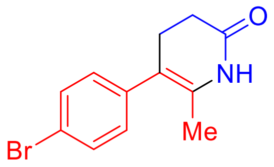

5-(4-Bromophenyl)-6-methyl-3,4-dihydropyridin-2(1H)-one (5a).
The title compound was isolated by column chromatography (petroleum ether/ethyl acetate = 4:1) as a white solid in 50% yield. **^{1}H-NMR** (800 MHz, DMSO-d_6) δ 9.23 (s, 1H), 7.49 (d, J = 8.8 Hz, 2H), 7.16 (d, J = 8.5 Hz, 2H), 2.52 (ddd, J = 8.4, 7.1, 1.5 Hz, 2H), 2.40 (dd, J = 8.9, 7.1 Hz, 2H), 1.77 (s, 3H). **^{13}C-NMR** (200 MHz, DMSO-d_6) δ 170.4, 140.3, 131.4, 131.4, 130.8, 130.8, 119.2, 111.0, 31.2, 27.0, 17.1. **HRMS (ESI):** Calculated for $C_{12}H_{13}BrNO^+$ $[M+H]^+$ 266.0175, found 266.0180.

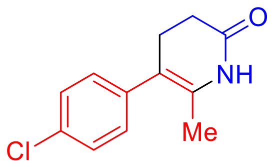

5-(4-Chlorophenyl)-6-methyl-3,4-dihydropyridin-2(1H)-one (5b).
The title compound was isolated by column chromatography (petroleum ether/ethyl acetate = 4:1) as a white solid in 64% yield. **^{1}H-NMR** (800 MHz, DMSO-d_6) δ 9.23 (s, 1H), 7.38 – 7.37 (m, 2H), 7.24 – 7.22 (m, 2H), 2.53 (ddd, J = 8.3, 7.0, 1.5 Hz, 2H), 2.40 (dd, J = 8.8, 7.1 Hz, 2H), 1.77 (s, 3H). **^{13}C-NMR** (200 MHz, DMSO-d_6) δ 170.4, 139.9, 139.9, 130.8, 130.8, 130.5, 130.5, 128.5, 110.9, 31.2, 27.1, 17.1. **HRMS (ESI):** Calculated for $C_{12}H_{13}ClNO^+$ $[M+H]^+$ 222.0680, found 222.0684.

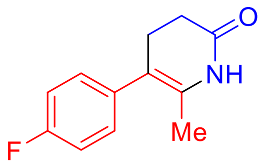

5-(4-Fluorophenyl)-6-methyl-3,4-dihydropyridin-2(1H)-one (5c).
The title compound was isolated by column chromatography (petroleum ether/ethyl acetate = 4:1) as a white solid in 80% yield. **^{1}H-NMR** (800 MHz, DMSO-d_6) δ 9.20 (s, 1H), 7.24 (dd, J = 8.5, 5.7 Hz, 2H), 7.15 (t, J = 8.8 Hz, 2H), 2.53 (t, J = 8.2 Hz, 2H), 2.41 (t, J = 7.9 Hz, 2H), 1.76 (s, 3H). **^{13}C-NMR** (200 MHz, DMSO-d_6) δ 170.4, 160.9 (d, J=240Hz), 137.3, 130.6, 130.6, 130.2, 115.3, 115.3, 115.2, 111.2, 31.2, 27.3, 17.0. **^{19}F-NMR** (377 MHz, DMSO) δ -116.7. **HRMS (ESI):** Calculated for $C_{12}H_{13}FNO^+$ $[M+H]^+$ 206.0976, found 206.0979.

4. Synthetic procedure for kilogram synthesis of 3a, 3d and 3g

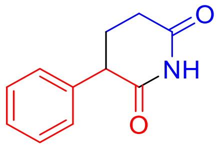

3-Phenylpiperidine-2,6-dione (3a).

To a 100 L reaction vessel was added 8 kg DMF and 5 kg acrylamide **2a** at room temperature. The temperature was decreased to -20 °C and 9.5 kg KO*t*Bu was added. Subsequently, 12.5 kg **1a** in 8 kg DMF was added slowly and stirred at this temperature for 0.5 h, then 0 °C for another 6 h. The reaction was quenched by the addition of 14 L aq. HCl (6 N). The precipitate was filtered and washed with water (50 L), then recrystallized in DMF/anhydrous ethanol (v/v=1:1, 32 L) to obtain **3a** as a white solid in 80% yield. Kilogram synthesis of compound **3d** (70% yield) and **3g** (71% yield) were prepared in a similar procedure listed below.

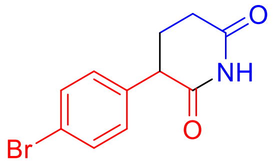

3-(4-Bromophenyl)piperidine-2,6-dione (3d).

To a 100 L reaction vessel was added 8 kg DMF and 5 kg acrylamide **2a** at room temperature. The temperature was decreased to -20 °C and 9.5 kg KO*t*Bu was added. Subsequently, 19.3 kg **1d** in 8 kg DMF was added slowly and stirred at this temperature for 0.5 h, then 0 °C for another 6 h. The reaction was quenched by the addition of 14 L aq. HCl (6 N). The precipitate was filtered and washed with water (50 L), then recrystallized with DMF/anhydrous ethanol (v/v=1:1, 32 L) to obtain **3d** as a white solid in 70% yield.

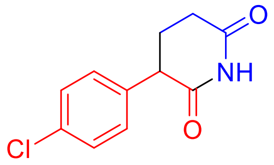

3-(4-Chlorophenyl)piperidine-2,6-dione (3g).

To a 100 L reaction vessel was added 8 kg DMF and 5 kg acrylamide **2a** at room temperature. The temperature was decreased to -20 °C and 9.5 kg KO*t*Bu was added. Subsequently, 15.6 kg **1g** in 8 kg DMF was added slowly and stirred at this temperature for 0.5 h, then 0 °C for another 6 h. The reaction was quenched by the addition of 14 L aq. HCl (6 N). The precipitate was filtered and washed with water (50 L), then recrystallized with DMF/anhydrous ethanol (v/v=1:1, 32 L) to obtain **3d** as a white solid in 71% yield.

5. Further transformations

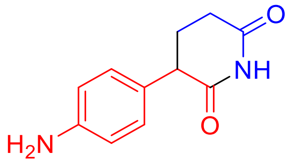

Synthesis of 3-(4-Aminophenyl)piperidine-2,6-dione (6) [7]

(1) Compound **3t** as starting material (De-Boc process)

3t (6 mmol) and concentrated HCl (0.4 ml, 13 N) 30 mL of 1,4-dioxane was added and stirred at room temperature for 8 h. The reaction mixture was quenched by the slow addition of water (200 mL) and saturated $NaHCO_3$ (350 ml), then extracted with DCM (100 ml *3), concentrated under reduced pressure, the title compound was isolated by column chromatography (petroleum ether/ethyl acetate = 4:1) as a pale-yellow solid in 86% yield.

(2) Compound **3k** as starting material (Nitro reduction process)

3k (6 mmol) was dissolved in methanol (12 ml) in an autoclave, and 100 mg of Pd/C was added into the reactor. Then N_2 was charged into the reaction system to remove air, H_2 was charged and stirred at room temperature for 24 h. The resulting mixture was concentrated under reduced pressure, crude product was obtained and purified by column chromatography (petroleum ether/ethyl acetate = 4:1) as a pale-yellow solid in 92% yield. **^{1}H-NMR** (800 MHz, DMSO-d_6) δ 10.79 (s,1H), 7.39 (d, J = 7.9 Hz,2H), 7.09 (d, J = 8.6 Hz,2H), 5.01(s,2H), 3.76 (dd, J = 11.4, 4.9 Hz,1H), 2.64 (dd, J = 17.1, 11.7, 5.3 Hz,1H), 2.49 – 2.45 (m,1H), 2.14 (dd, J = 13.0, 11.7, 5.8 Hz,1H), 2.02 – 1.99 (m,1H). **^{13}C-NMR** (200 MHz, DMSO-d_6) δ 175.3, 174.0, 147.9, 129.3, 129.3, 126.3, 114.3, 114.3, 46.9, 31.5, 26.5. **HRMS (ESI):** Calculated for $C_{11}H_{13}N_2O^+$ [M+H]$^+$ 205.0971, found 205.0971.

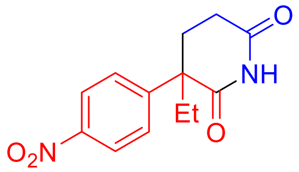

3-Ethyl-3-(4-nitrophenyl)piperidine-2,6-dione.[13]

3aa (6 mmol) in concentrated sulphuric acid (3 mL) at -10℃ was added into the mixture of sulphuric acid (1 g) and 63% azuric acid (1 g) dropwise, then the resulting mixture was stirred at room temperature for 4 h. The reaction mixture was poured onto ice and extracted with DCM (20 ml * 3), crude product was obtained and further purified by column chromatography (petroleum ether/ethyl acetate = 4:1) as a pale-yellow solid in 85% yield. **^{1}H-NMR** (800 MHz, DMSO-d_6) δ 11.03 (s, 1H), 8.25 (d, J = 8.9 Hz, 2H), 7.61 (d, J = 8.9 Hz, 2H), 2.53 (dd, J = 7.7, 3.0 Hz, 1H), 2.45 – 2.42 (m, 1H), 2.27 – 2.23 (m, 1H), 2.14 (ddd, J = 17.8, 12.6, 5.1 Hz, 1H), 1.95 (dq, J = 14.7, 7.4 Hz, 1H), 1.89 (dq, J = 14.6, 7.3 Hz, 1H), 0.78 (t, J = 7.4 Hz, 3H). **^{13}C-NMR** (200 MHz, DMSO-d_6) δ 175.5, 173.2, 148.3, 147.0, 128.5, 128.5, 124.3, 124.3, 51.1, 32.1, 29.4, 26.6, 9.3. **HRMS (ESI):** Calculated for $C_{13}H_{15}N_2O_4^+$ [M+H]$^+$ 263.1026, found 263.1034.

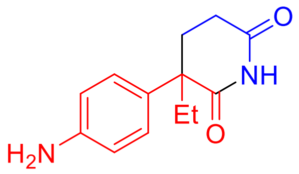

Synthesis of Aminoglutethimide[7]

3-Ethyl-3-(4-nitrophenyl)piperidine-2,6-dione (2 mmol) was dissolved in methanol (25 ml) in an autoclave, then 25 mg of Pd/C added. N_2 was charged into the reactor to remove air, then H_2 was charged and stirred at room temperature for 24 h. Then Pd/C was removed by filtration and the filtrate was concentrated under reduced pressure, crude product was obtained and further purified by column chromatography (petroleum ether/ethyl acetate = 4:1) as a pale-yellow solid in 95% yield. **^{1}H-NMR** (800 MHz, DMSO-d_6) δ 10.72 (s, 1H), 6.92 (d, J = 8.6 Hz, 2H), 6.55 (d, J = 8.6 Hz, 2H), 5.09 (s, 2H), 2.43 – 2.41 (m, 1H), 2.25 – 2.23 (m, 1H), 2.17 – 2.14 (m, 1H), 2.07 (dd, J = 13.5, 4.4 Hz, 1H), 1.79 (dd, J = 13.9, 7.3 Hz, 1H), 1.74 – 1.71 (m, 1H), 0.73 (t, J = 7.4 Hz, 3H). **^{13}C-NMR** (200 MHz, DMSO-d_6) δ 176.8, 173.5, 147.9, 127.2, 127.2, 126.6, 114.5, 114.5, 49.7, 32.8, 29.7, 26.4, 9.4. **HRMS (ESI):** Calculated for $C_{13}H_{17}N_2O_2^+$ $[M+H]^+$ 233.1285, found 233.1287.

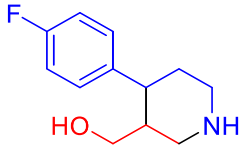

(4-(4-fluorophenyl)piperidin-3-yl)methanol (7d).[14]

In a 50 ml reaction flask, **3ad** (38 mmol) was mixed with THF (30 ml) at -20°C for 5 min, then borane dimethylsulfide (10.5 mL) was added dropwise. During the process, the solid gradually dissolved, after the addition, the reaction mixture was refluxed for 6.0 h until the complete consumption of raw material monitored by TLC. The reaction mixture was cooled to room temperature and quenched by the addition of aq. K_2CO_3 (30 ml, 10wt%), then extracted with EtOAC (10 ml *3), filtered and concentrated under reduced pressure. Crude product 7**d** was isolated and purified by column chromatography (1:5 = methanol/dichloromethane) as a white solid in 73% yield. **^{1}H-NMR** (500 MHz, DMSO-d_6) δ 7.25 – 7.20 (m, 2H), 7.14 – 7.09 (m, 2H), 3.25 (d, J = 10.0 Hz, 1H), 3.09 (dd, J = 10.7, 3.0 Hz, 1H), 3.04 (d, J = 11.9 Hz, 1H), 2.94 (dd, J = 10.7, 7.8 Hz, 1H), 2.63 – 2.54 (m, 1H), 2.42 (dt, J = 12.1, 9.6 Hz, 2H), 1.80 – 1.74 (m, 2H), 1.64 (ddd, J = 11.9, 11.1, 3.4 Hz, 2H). **^{13}C-NMR** (125 MHz, DMSO-d_6) δ 161.1(d, J = 240.0 Hz), 141.5 (d, J = 3.0 Hz), 129.4 (d, J = 7.8 Hz), 115.5, 115.4, 62.0, 49.5, 46.1, 44.0, 43.8, 34.5.

6. Synthesis of Niraparib

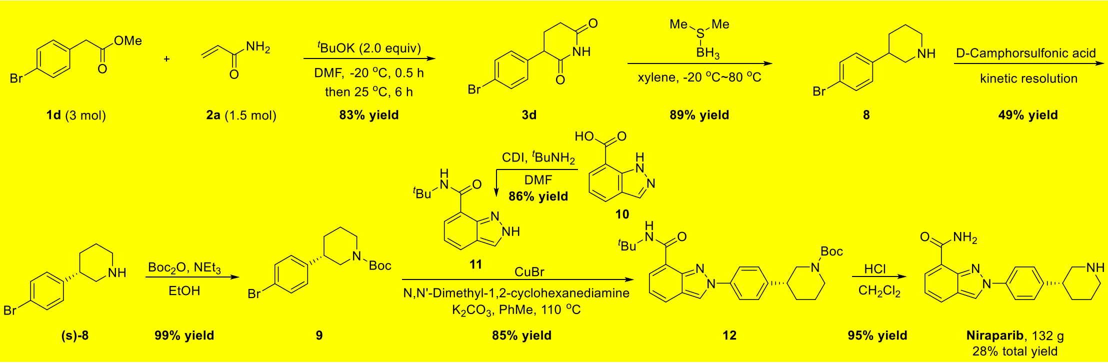

Figure S1 Synthetic route of Niraparib

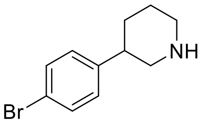

3-(4-Bromophenyl)piperidine (8).

Add xylene (800 mL), 3-(4-bromophenyl)piperidine-2,6-dione (1.2 mol) into 2 L reaction bottle, mechanical stirring, warm up to 70 °C slowly drop borane dimethyl sulfide solution (340 mL). During the process of solid gradually dissolved, there is white flocculent precipitation, drop finished refluxing the reaction for 4h. TLC monitor raw materials to complete the reaction, turn off the heating, the system down to room temperature. Slowly addition of aq 10 wt% K_2CO_3 (1.0 kg) to quench the reaction. Atmospheric distillation of xylene to no liquid outflow after distillation under reduced pressure, receiving temperature of 120 °C fraction is the target compound **8** (89%). **^{1}H-NMR** (400 MHz, $CDCl_3$) δ 7.33 (d, J = 16.8 Hz, 2H), 7.01 (d, J = 16.8 Hz, 2H), 3.04 (t, J = 11.9 Hz, 2H), 2.55 (ddd, J = 12.4, 8.4, 3.2 Hz, 3H), 1.93 – 1.85 (m, 1H), 1.71 (d, J = 7.4 Hz, 2H), 1.55 – 1.43 (m, 2H). **^{13}C-NMR** (100 MHz, $CDCl_3$) δ 143.9, 131.4, 131.4,128.9, 128.9,119.9, 53.9, 46.6, 43.8, 32.1, 27.0. **HRMS (ESI):** Calculated for $C_{11}H_{15}BrN_2^+$ $[M+H]^+$ 240.0382, found 240.0384.

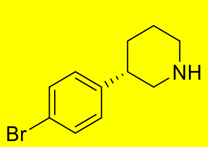

(*S*)-3-(4-bromophenyl)piperidine-2,6-dione [(*S*)-8].

3-(4-Bromophenyl)piperidine(1.1 mol) of the raw material was dissolved in 4.5 L of hot isopropanol: ethyl acetate (20:3) solution, and after complete dissolution an equivalent of D-Camphorsulfonic acid was added, refluxed for one hour and then cooled to 5 °C overnight. The crystalline precipitate was filtered and washed with cold isopropanol and dried under vacuum to an ee value of 99%. To the solid salt was added NaOH solution (1.8 L, 1M), then 2 L of ether was added and stirred. When all the solids were dissolved the two phases were partitioned, the organic phase was dried with anhydrous sodium sulphate, and dried to give a white solid **(s)-8** with 49% yield.

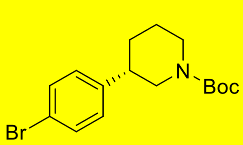

(S)-*tert*-butyl 3-(4-bromophenyl)piperidine-1-carboxylate (9).[15]

3-(4-Bromophenyl)piperidine (0.54 mol) was dissolved in 2 L of anhydrous ethanol, Boc anhydride (0.54 mol) was added and stirred overnight at room temperature. The reaction solution was extracted with water and ethyl acetate. Spin evaporation to give a yellow oily liquid, which crystallizes to a white solid **9** (99%) on standing. **^{1}H-NMR** (800 MHz, DMSO-d_6) δ 7.52 – 7.47 (m,2H), 7.23 (d, J = 8.5 Hz,2H), 3.93 (d, J = 54.0 Hz,2H), 2.79 (dd, J = 61.1, 55.3 Hz,2H), 2.59 (t, J = 11.4, 3.5 Hz,1H), 1.86 – 1.83 (m,1H), 1.69 – 1.66 (m,1H), 1.59 (q, J = 12.5, 3.5 Hz,1H), 1.46 – 1.42 (m,1H), 1.42 – 1.36 (m,9H). **^{13}C-NMR** (200 MHz, DMSO-d_6) δ 154.3, 143.4, 131.8, 131.8, 129.8, 129.8,112.0, 79.1, 50.0, 44.0, 41.9, 31.6, 28.5, 28.5, 28.5, 25.6. **HRMS (ESI):** Calculated for $C_{11}H_{15}BrN_2^+$ $[M+H]^+$ 340.0907, found 340.0909.

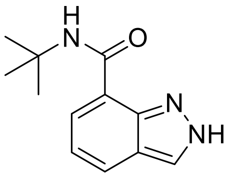

***N*-(*tert*-butyl)-2*H*-indazole-7-carboxamide (11).**[15]

CDI (1.2 mol) was added to a solution of indazole **10** (1 mol) in DMF (750 mL) at 20-25 °C. After 30 min, the reaction mixture was evacuated. After an additional 25 min, ^{t}BuNH$_2$ (2 mol) was added within 3 min. Without external cooling, the batch temperature is raised from room temperature to 40 °C in 40 min. The mixture is then aged for 2.5 hours. After cooling to room temperature, water (300 mL) was added, and the temperature was kept below 25 °C. The crystalline solution was then added to the solution. Crystalline product (0.7 g) was then added to the solution and the slurry was aged at 20-25 °C for 30 minutes. Water (1500 mL) was then added over 80 minutes and the slurry was allowed to age overnight. The slurry was filtered, washed sequentially with 9:1 v/v water/DMF (750 mL) and water (2 × 750 mL), and vacuum dried at 20-25 °C under N_2 purging to yield a beige powder **11**. **^{1}H-NMR** (400 MHz, DMSO-d_6) δ 13.05 (s,1H), 8.13 (s,1H), 7.92 (t, J = 6.4 Hz,3H), 7.16 (t, J = 7.6 Hz,1H), 1.46 (s,9H). **^{13}C-NMR** (100 MHz, DMSO-d_6) δ 166.3, 138.2, 133.9, 125.6, 124.7, 124.3, 119.9, 1188, 51.4, 29.2, 29.2, 29.2. **HRMS (ESI):** Calculated for $C_{12}H_{16}N_3O^+$ $[M+H]^+$ 218.1288, found 218.1292.

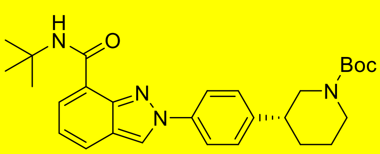

(S)-*tert*-butyl 3-(4-(7-(tert-butylcarbamoyl)-2*H*-indazol-2-yl)phenyl)piperidine-1-carboxylate (12).[15]

The *tert*-butyl 3-(4-bromophenyl)piperidine-1-carboxylate **9** (0.53 mol) was dissolved in toluene (1L). To the reaction system was added to *N*-(*tert*-butyl)-1H-indazole-7-carboxamide (0.53 mol) and K_2CO_3 (1.5 mol) and bubbled with nitrogen at 20-25°C for 1 h. CuBr (25 mmol) and *N*, *N*'-

dimethyl-1,2-cyclohexanediamine (50 mmol) were added, and N_2 sparging continued for 30 min. The reaction mass was then heated to 110 °C for 24 h. After cooling to 40 °C, Celite (130 g) was added, and the mixture aged for 1 h before being filtered, washing the cake with DMAc (800 mL). The combined filtrates were adjusted to 35 °C, then toluene (400 mL) and 10% aq. citric acid (260 mL) were added, followed by seed crystals (3 g) of product. After 1 h additional 10% aq. citric acid (55 mL) was added over 100 min. The resulting slurry was aged for 2 h at 35 °C then at 20−25 °C overnight. Filtration, washing with 2:1 v/v DMAc/water (1.6 L) followed by water (2.7 L), and drying in vacuo at 20−25 °C under a N_2 sweep afforded **12** as a light-yellow power. **^{1}H-NMR** (800 MHz, $CDCl_3$) δ 9.25 (s, 1H), 8.41 (s, 1H), 8.17 (d, *J* = 7.0 Hz, 1H), 7.73 (dd, *J* = 18.3, 7.9 Hz, 3H), 7.33 (d, *J* = 8.5 Hz, 2H), 7.14 – 7.11 (m, 1H), 4.14 (d, *J* = 122.3 Hz, 2H), 2.69 (s, 3H), 2.24 – 2.20 (m, 1H), 1.98 (d, *J* = 13.2 Hz, 1H), 1.70 (d, *J* = 13.0 Hz, 1H), 1.59 (dd, *J* = 12.3, 3.1 Hz, 1H), 1.50 (s, 9H), 1.41 (d, *J* = 15.5 Hz, 9H). **^{13}C-NMR** (200 MHz, DMSO-*d*$_6$) δ 163.1, 153.8, 145.7, 142.9, 137.4, 128.8, 127.4, 127.4, 125.0, 123.0, 122.5, 121.8, 121.6.0, 120.1, 119.5, 78.6, 50.2, 40.9, 30.6, 28.0, 28.0, 28.0, 27.5, 27.45, 27.5, 26.0. **HRMS (ESI):** Calculated for $C_{28}H_{37}N_4O_3^+$ $[M+H]^+$ 477.2760, found 477.2853.

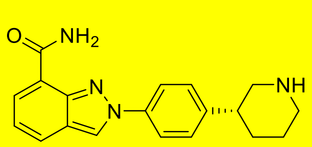

(S)-2-(4-(Piperidin-3-yl)phenyl)-2*H*-indazole-7-carboxamide (Niraparib).

The HCl (40%, 3 mol) was added into a stirred solution of **12** (0.44 mol) in DCM (2.5 L) and the reaction system was stirred at 40°C for 36 h. The reaction was quenched with saturated $NaHCO_3$, extracted with dichloromethane, and concentrated at low pressure. Ultimately, a white solid was obtained in 95% yield. **^{1}H-NMR** (400 MHz, DMSO-*d*$_6$) δ 9.26 (s, 1H), 8.59 (s, 1H), 8.08 (dd, *J* = 7.0, 0.9 Hz, 1H), 8.02 (dd, *J* = 12.6, 4.8 Hz, 3H), 7.92 (s, 1H), 7.46 (d, *J* = 8.6 Hz, 2H), 7.27 (dd, *J* = 8.3, 7.1 Hz, 1H), 2.97 (dd, *J* = 25.3, 12.0 Hz, 2H), 2.70 (ddd, *J* = 11.0, 7.3, 3.9 Hz, 1H), 2.55 (d, *J* = 11.4 Hz, 1H), 2.47 (dd, *J* = 12.0, 2.3 Hz, 1H), 1.90 (d, *J* = 12.9 Hz, 1H), 1.78 – 1.32 (m, 4H). **^{13}C-NMR** (100 MHz, DMSO-*d*$_6$) δ 166.1, 146.5, 146.2, 138.0, 130.1, 128.7, 128.7, 125.8, 123.8, 122.3, 121.9, 121.2, 121.2, 54.0, 46.4, 43.6.0, 32.3, 27.0. **HRMS (ESI):** Calculated for $C_{19}H_{21}N_4O^+$ $[M+H]^+$ 321.1710, found 321.1716.

7. Detection of possible reaction intermediate of 1a with 2a by LC-MS.

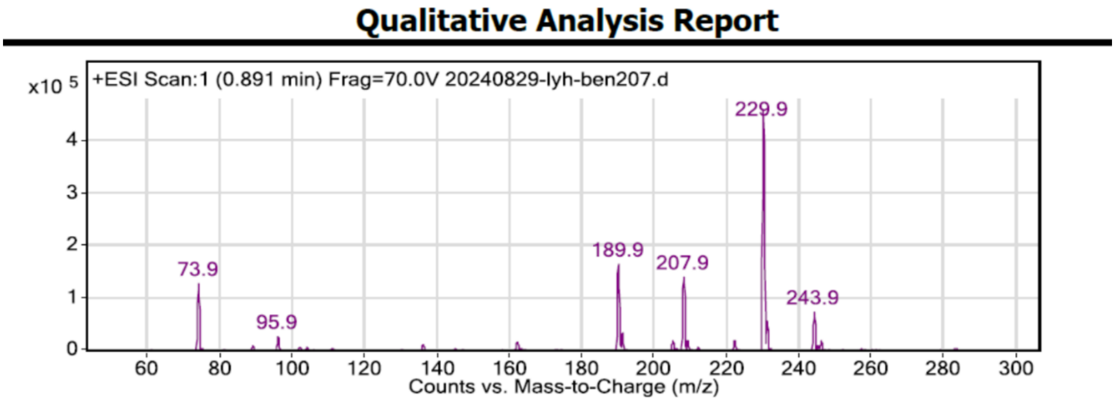

Figure S2 Intermediate detection by LC-MS

8. Chiral HPLC spectrum of intermediate *(S)*-8.

HPLC condition for the measurement of **(S)-8**: ODS-3 column(250 × 4.6 mm, 5 μm particle size);，Run time: 35.2 min; Flow rate: 0.8 mL/min; Pressure: 40 MPa; Sample injection volume: 10 μL; UV detection: 254 nm.

Eluent: A 相：0.1%Formic acid/water B 相：CH_3OH

Time (10min)	Pump	ratio (%)
0.1	B	50
5	B	70
30	B	95
35	B	5
35.2	stop	

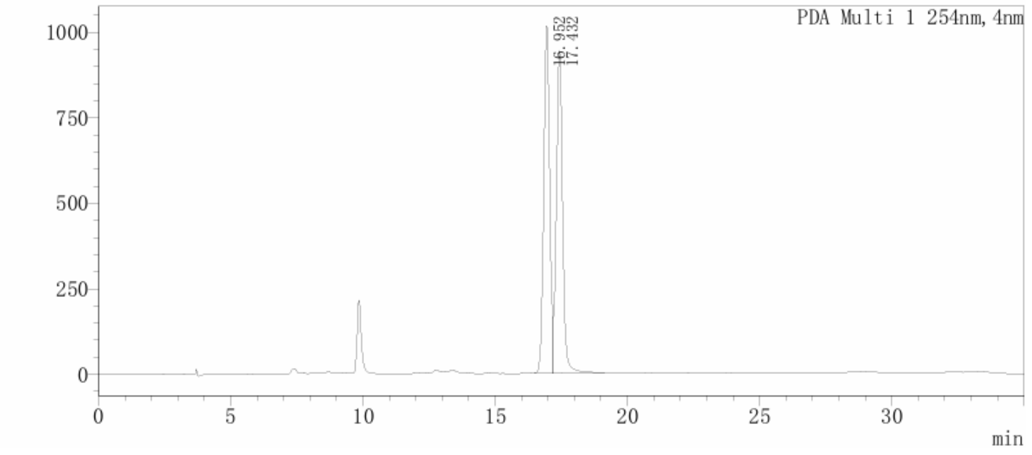

〈峰表〉
PDA Ch1 254nm

峰号	保留时间	面积	面积%	高度
1	16.952	16084617	50.790	1016751
2	17.432	15584258	49.210	943706
总计		31668875	100.000	1960457

Figure S3 Chiral HPLC Spectrum of racemic **8**

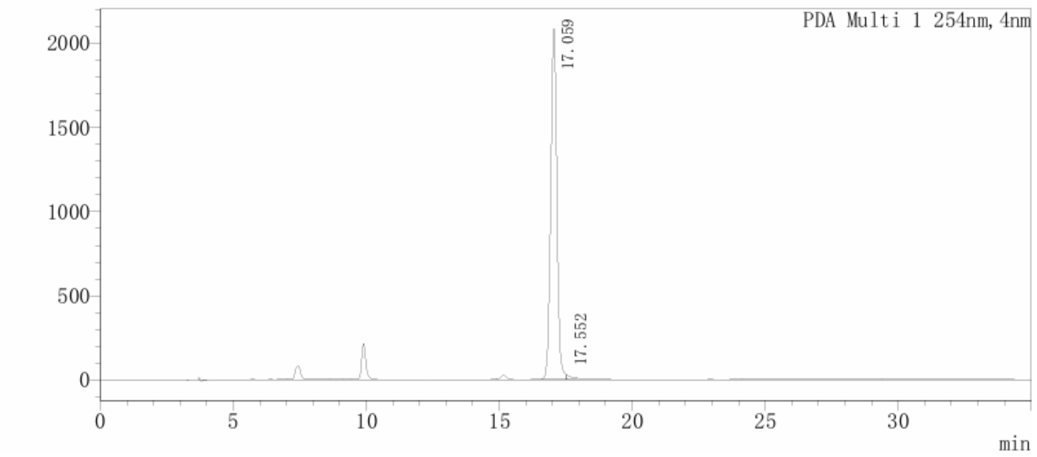

〈峰表〉
PDA Ch1 254nm

峰号	保留时间	面积	面积%	高度
1	17.059	33630832	99.297	2083856
2	17.552	238163	0.703	24950
总计		33868995	100.000	2108807

Figure S4 Chiral HPLC Spectrum of ***(S)*-8** (standard)

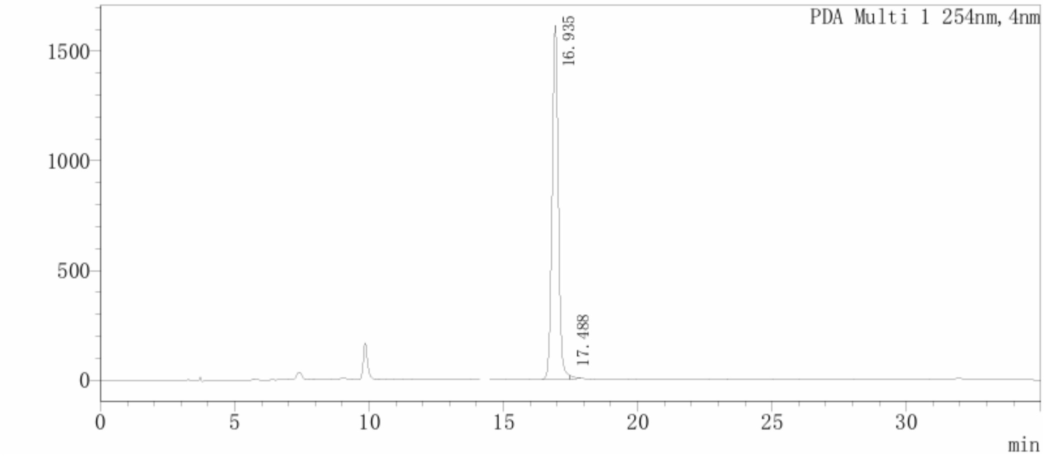

<峰表>
PDA Ch1 254nm

峰号	保留时间	面积	面积%	高度
1	16.935	26234021	99.323	1613734
2	17.488	178773	0.677	16709
总计		26412794	100.000	1630443

Figure S5 Chiral HPLC Spectrum of synthetic **(S)-8**

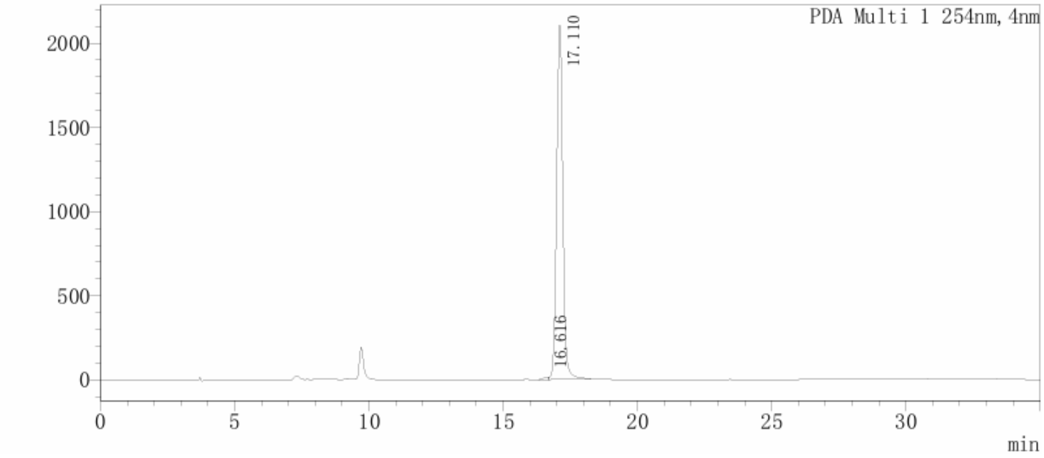

<峰表>
PDA Ch1 254nm

峰号	保留时间	面积	面积%	高度
1	16.616	199474	0.592	14996
2	17.110	33486535	99.408	2104222
总计		33686008	100.000	2119218

Figure S6 Chiral HPLC Spectrum of synthetic **(R)-8**

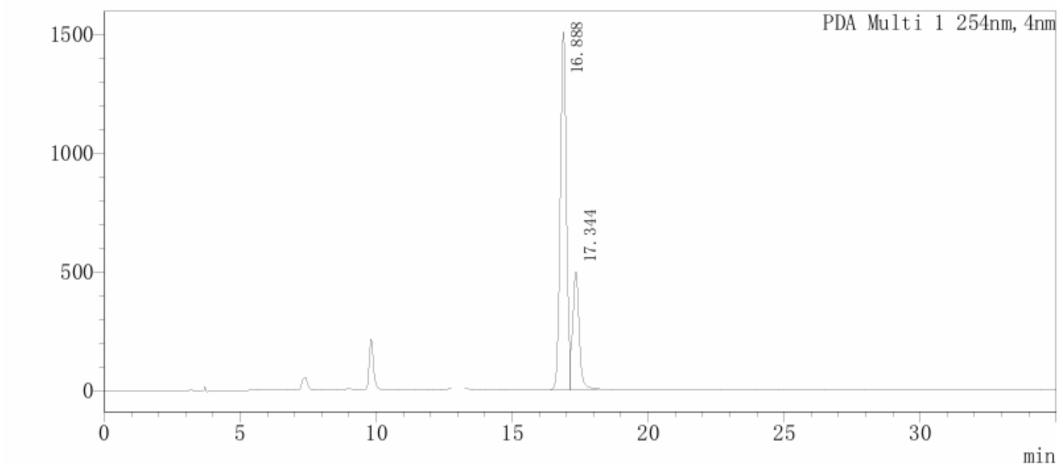

〈峰表〉

PDA Ch1 254nm

峰号	保留时间	面积	面积%	高度
1	16.888	23875421	74.701	1507778
2	17.344	8085826	25.299	495736
总计		31961247	100.000	2003514

Figure S7 Chiral HPLC spectrum of the mixture of synthetic **(S)-8** and racemic **8**

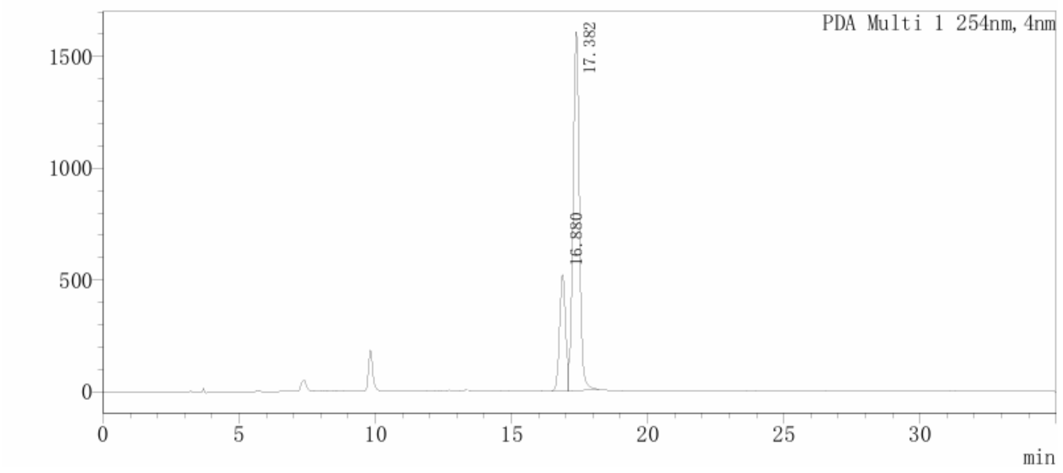

〈峰表〉

PDA Ch1 254nm

峰号	保留时间	面积	面积%	高度
1	16.880	8067319	23.748	519677
2	17.382	25902512	76.252	1606246
总计		33969831	100.000	2125923

Figure S8 Chiral HPLC spectrum of the mixture of synthetic **(R)-8** and racemic **8**

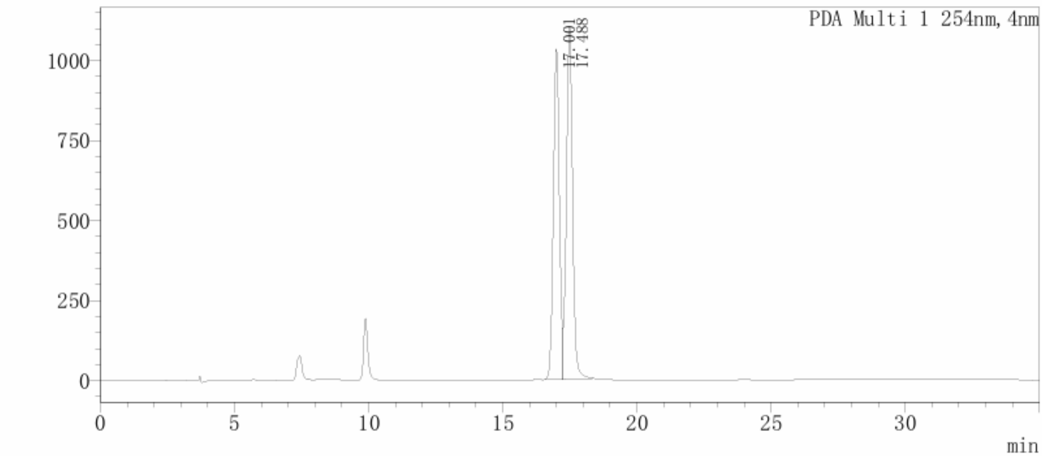

〈峰表〉

PDA Ch1 254nm

峰号	保留时间	面积	面积%	高度
1	17.001	16214203	47.840	1032467
2	17.488	17678342	52.160	1099911
总计		33892545	100.000	2132378

Figure S9 Chiral HPLC spectrum of the mixture of synthetic **(S)-8** and **(R)-8**

8. Reference

[1] M. Israr, H. Xiong, Y. Li, H. Bao, *Adv. Synth. Catal.* **2020**, *362*, 2211-2215.

[2] B. Yang, M. M. Weiss, X. Zhu, H. Dong, I. Marx*, Vol. WO2023278759A1*, WO2023278759A1 ed., **2023**.

[3] L. J. Alcock, Y. Chang, J. A. Jarusiewicz, M. Actis, S. Nithianantham, A. Mayasundari, J. Min, D. Maxwell, J. Hunt, B. Smart, J. J. Yang, G. Nishiguchi, M. Fischer, C. G. Mullighan, Z. Rankovic, *ACS Med. Chem. Lett.* **2022**, *13*, 475-482.

[4] G. B. Hammond, R. G. Plevey, P. Sampson, J. C. Tatlow, *Journal of Fluorine Chemistry* **1988**, *40*, 81-98.

[5] A. J. Phillips, C. G. NASVESCHUK, J. A. Henderson, Y. Liang, M. He, K. Lazarski, G. K. Veits*, Vol. WO2017197046A1*, **2017**.

[6] T. Kametani, W. Taub, D. Ginsburg, *Bulletin of the Chemical Society of Japan* **1958**, *31*, 860-861.

[7] Rolf W. Hartmann, C. Batzl, *J. Med. Chem.* **1986**, *29*, 1362-1369.

[8] K. J. Berger, J. L. Driscoll, M. Yuan, B. D. Dherange, O. Gutierrez, M. D. Levin, *Journal of the American Chemical Society* **2021**, *143*, 17366-17373.

[9] T. Kato, M. Noda, *CHEMICAL & PHARMACEUTICAL BULLETIN* **1974**, *22*, 2947-2952.

[10] A. R. Doumaux, Jr., D. J. Trecker, *The Journal of Organic Chemistry* **1970**, *35*, 2121-2125.

[11] M. M. Al-Arab, A. M. Issa, *Monatshefte für Chemie / Chemical Monthly* **1987**, *118*, 987-

991.

[12] M. Y. Shandala, A.-I. Y. Al-Khashab, M. Afzal, S. S. Ahmad, *Journal of Heterocyclic Chemistry* **1980**, *17*, 1605-1607.

[13] G. Fogliato, G. Fronza, C. Fuganti, P. Grasselli, S. Servi, *The Journal of Organic Chemistry* **1995**, *60*, 5693-5695.

[14] G. de Gonzalo, R. Brieva, V. M. Sánchez, M. Bayod, V. Gotor, *The Journal of Organic Chemistry* **2001**, *66*, 8947-8953.

[15] C. K. Chung, P. G. Bulger, B. Kosjek, K. M. Belyk, N. Rivera, M. E. Scott, G. R. Humphrey, J. Limanto, D. C. Bachert, K. M. Emerson, *Org. Process Res. Dev.* **2013**, *18*, 215-227.

9. Spectroscopic data

^{1}H NMR spectrum of 3-phenylpiperidine-2,6-dione (3a). ($CDCl_3$, 400 MHz)

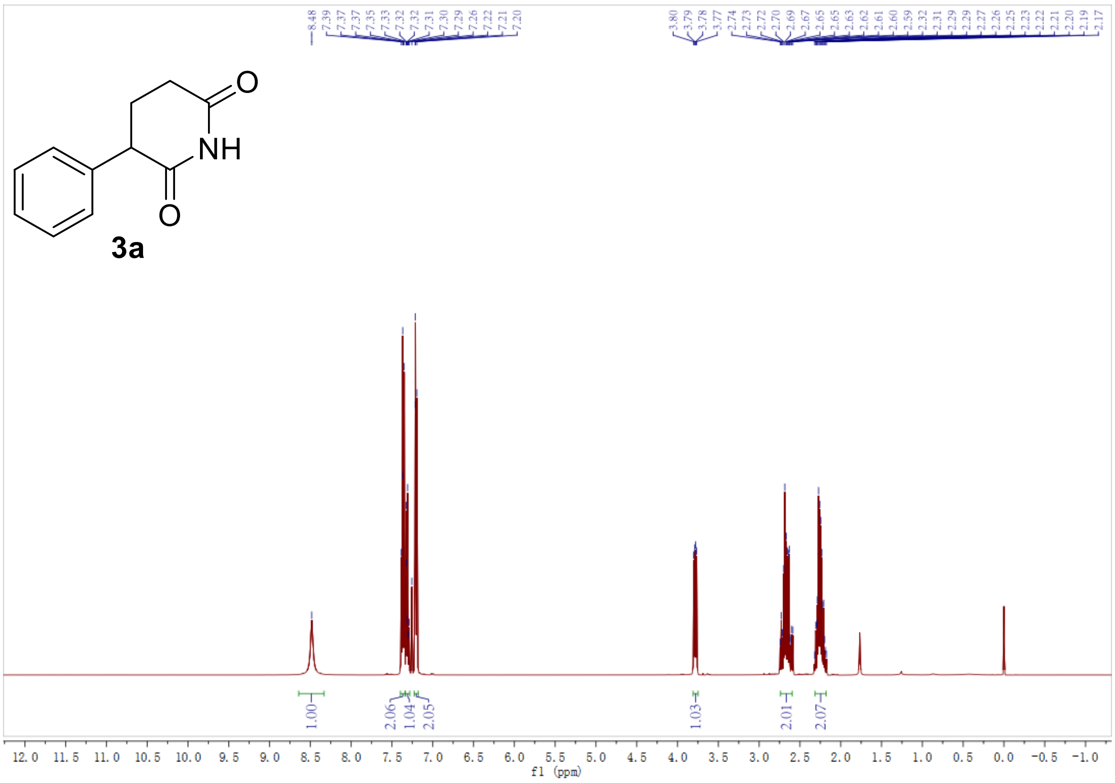

^{13}C NMR spectrum of 3-phenylpiperidine-2,6-dione(3a). ($CDCl_3$, 100 MHz)

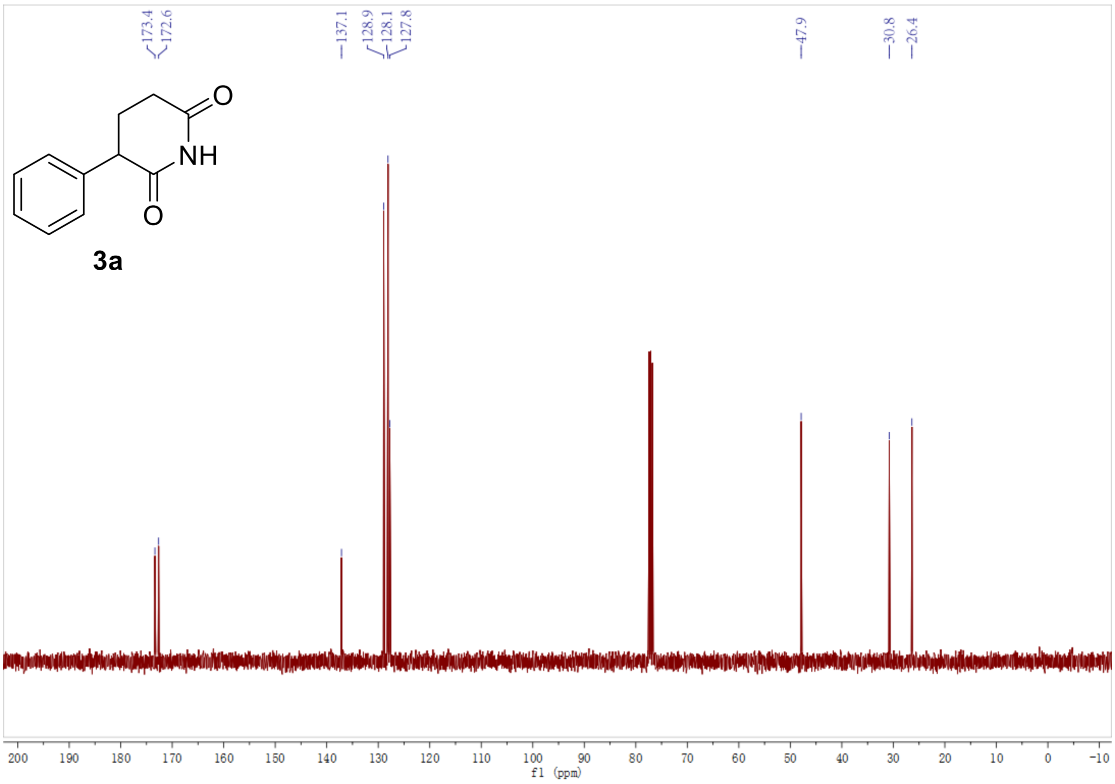

^{1}H NMR spectrum of 3-(2-bromophenyl)piperidine-2,6-dione (3b). (DMSO-d_6, 400 MHz)

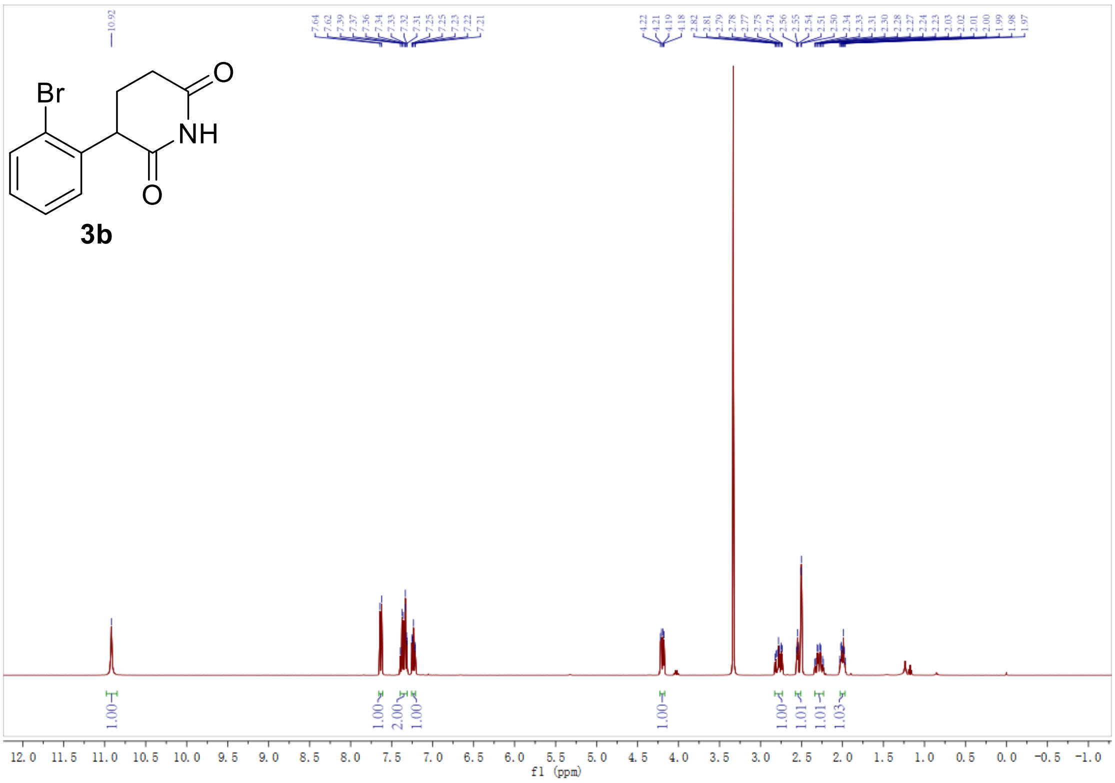

^{13}C NMR spectrum of 3-(2-bromophenyl)piperidine-2,6-dione (3b). (DMSO-d_6, 100 MHz)

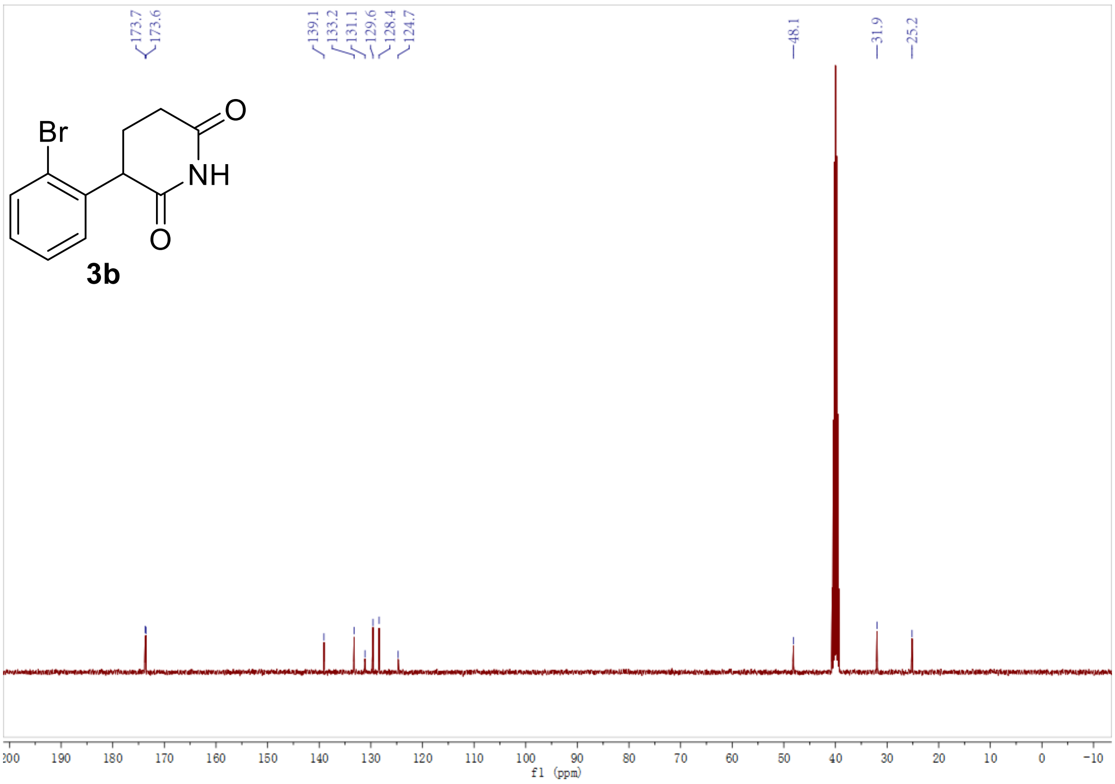

^{1}H NMR spectrum of 3-(3-bromophenyl)piperidine-2,6-dione (3c). (DMSO-d_6, 800 MHz)

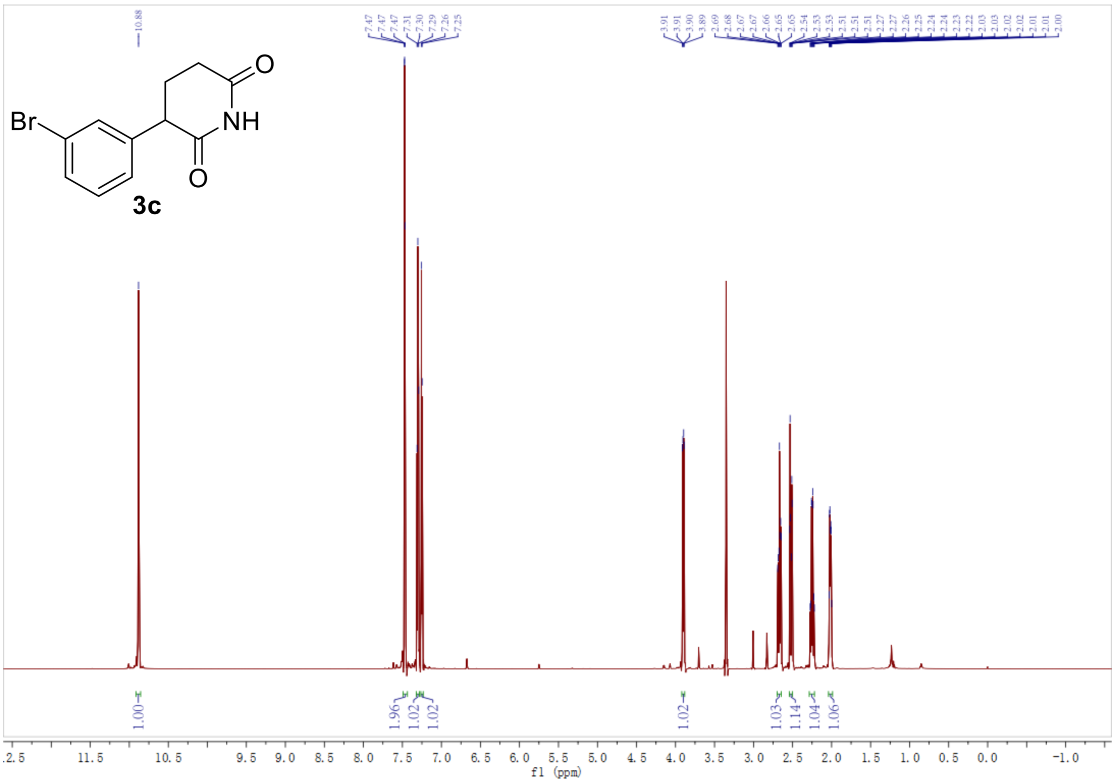

^{13}C NMR spectrum of 3-(3-bromophenyl)piperidine-2,6-dione (3c). (DMSO-d_6, 200 MHz)

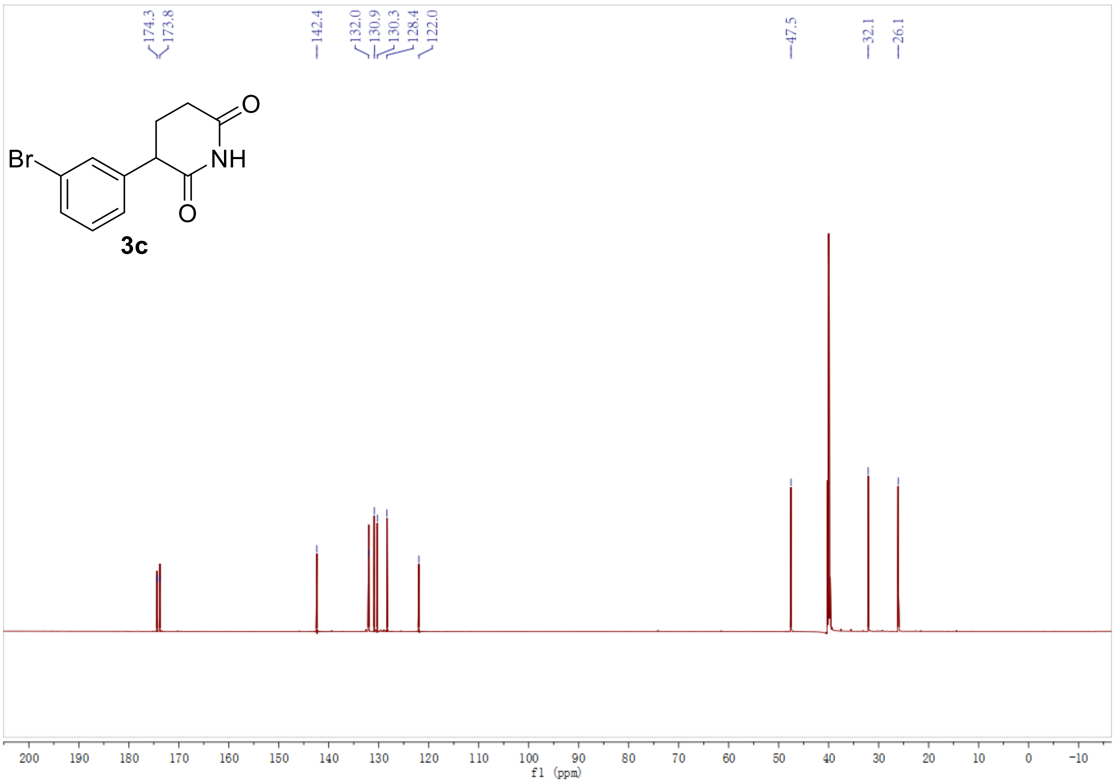

^{1}H NMR spectrum of 3-(4-bromophenyl)piperidine-2,6-dione (3d). (DMSO-d_6, 800 MHz)

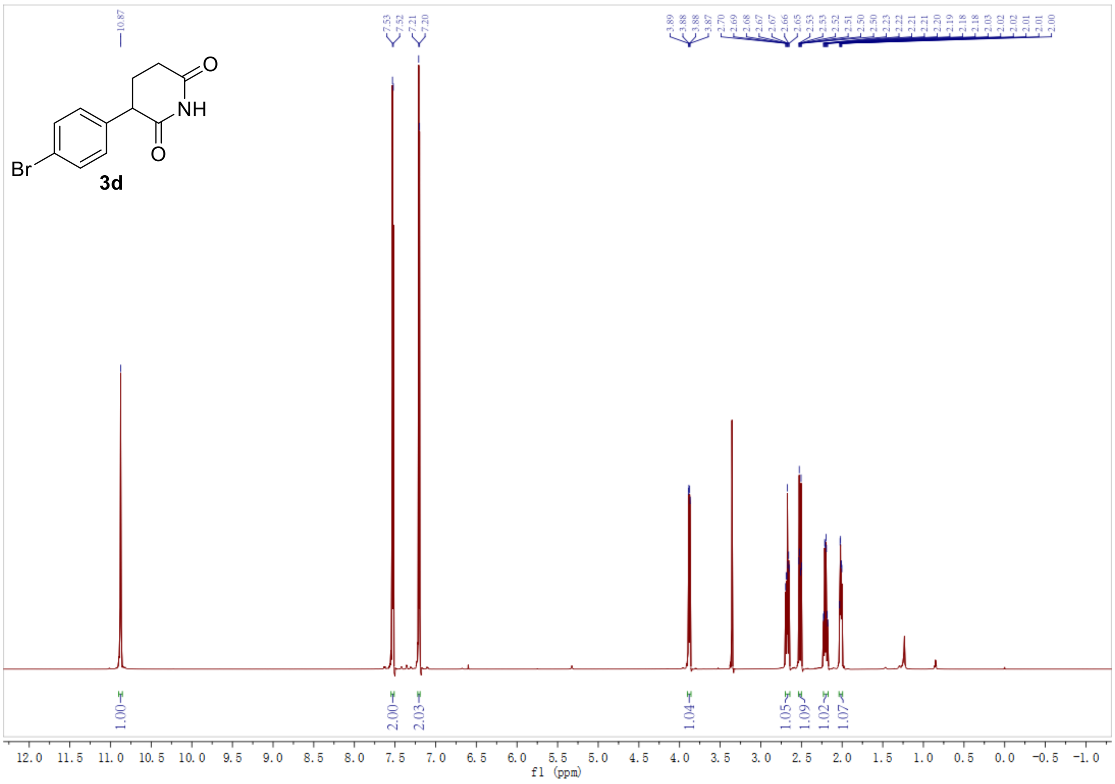

^{13}C NMR spectrum of 3-(4-bromophenyl)piperidine-2,6-dione (3d). (DMSO-d_6, 200 MHz)

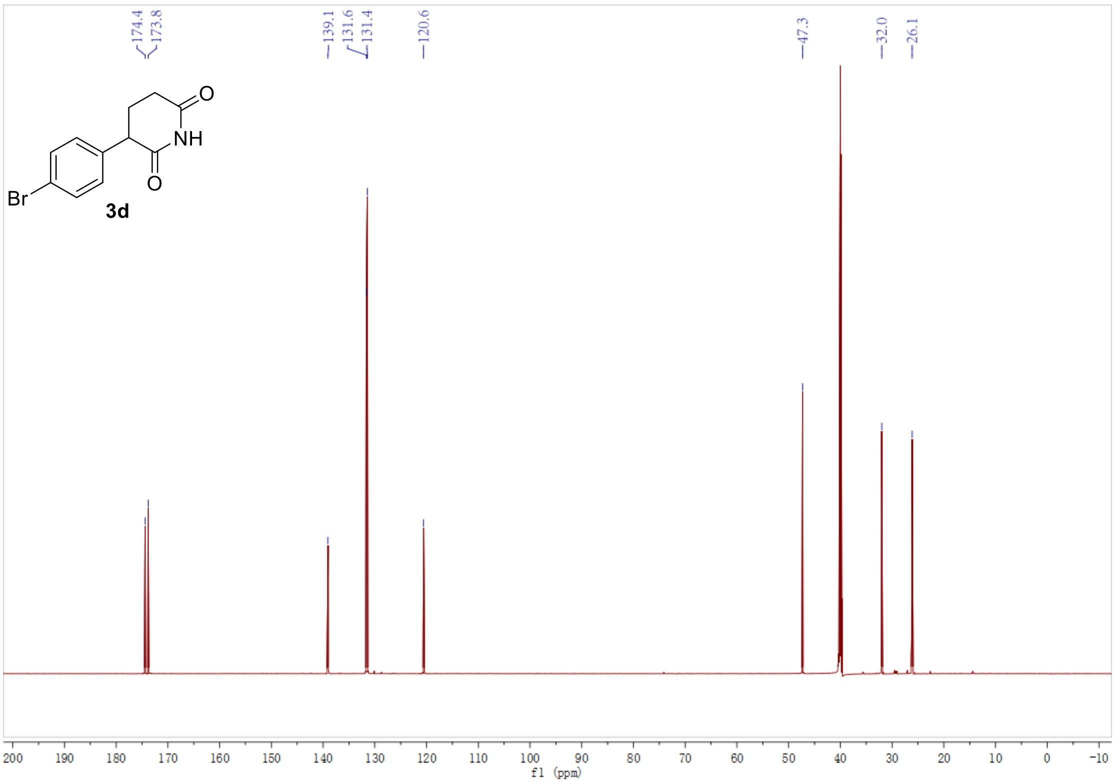

^{1}H NMR spectrum of 3-(2-chlorophenyl)piperidine-2,6-dione (3e). (DMSO-d_6, 800 MHz)

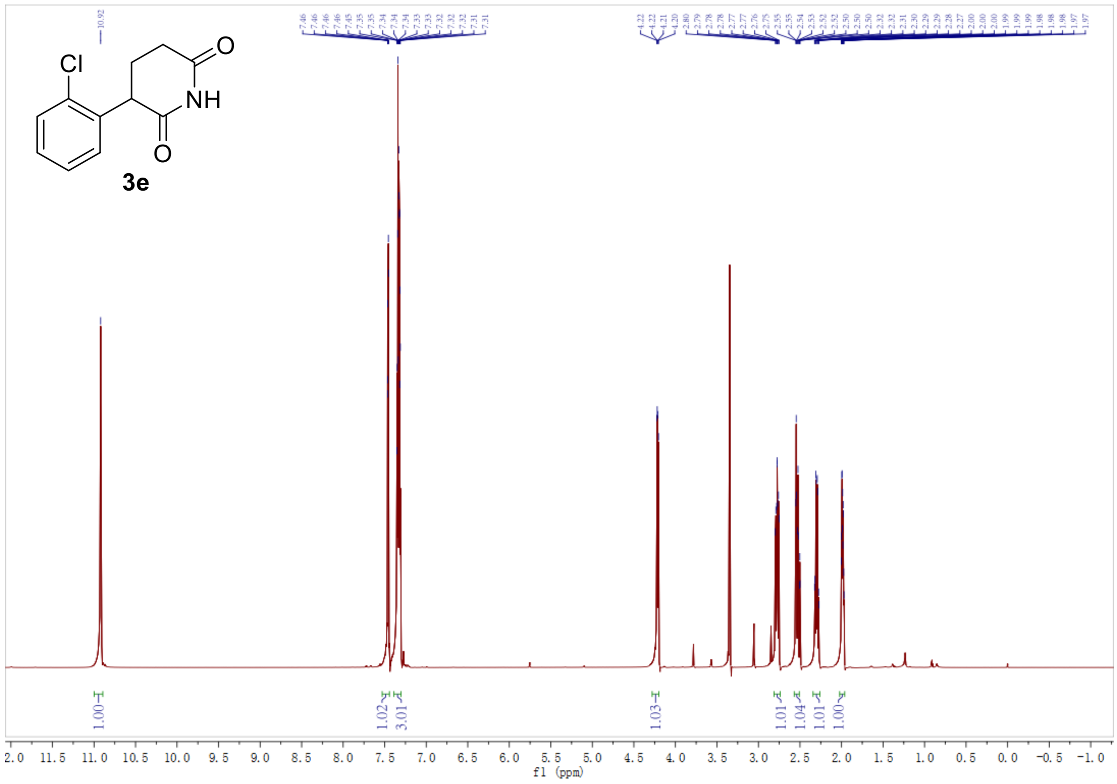

^{13}C NMR spectrum of 3-(2-chlorophenyl)piperidine-2,6-dione (3e). (DMSO-d_6, 200 MHz)

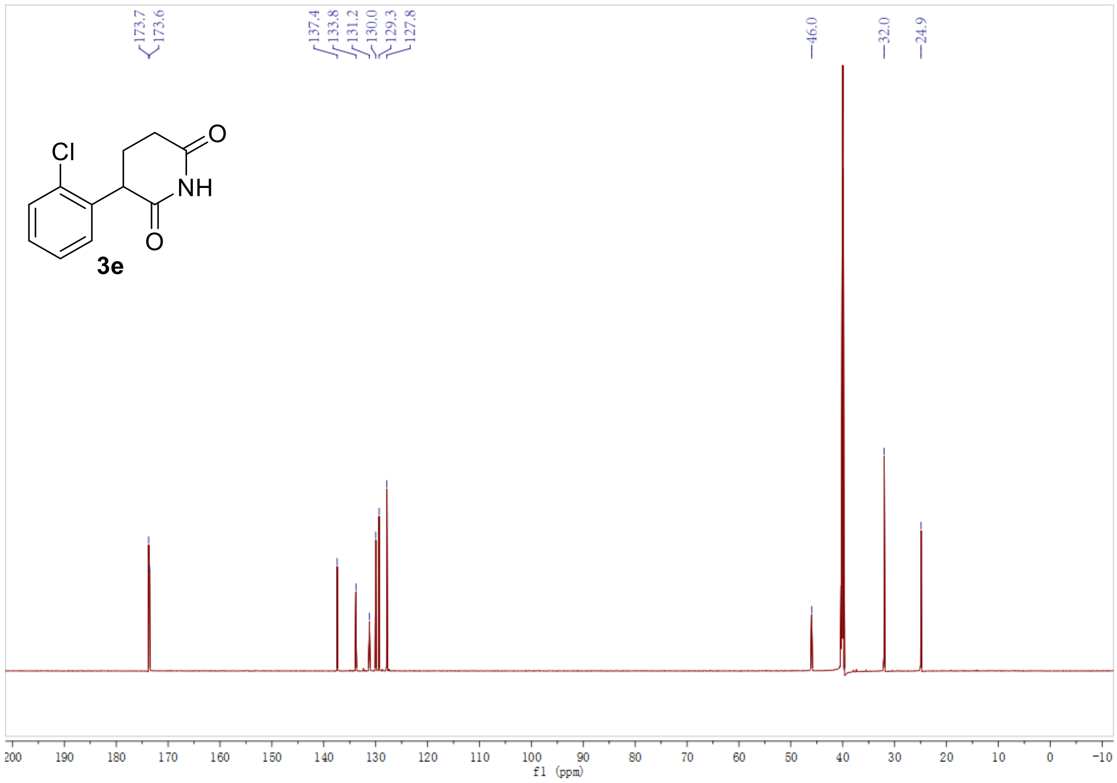

^{1}H NMR spectrum of 3-(3-chlorophenyl)piperidine-2,6-dione (3f). (DMSO-d_6, 800 MHz)

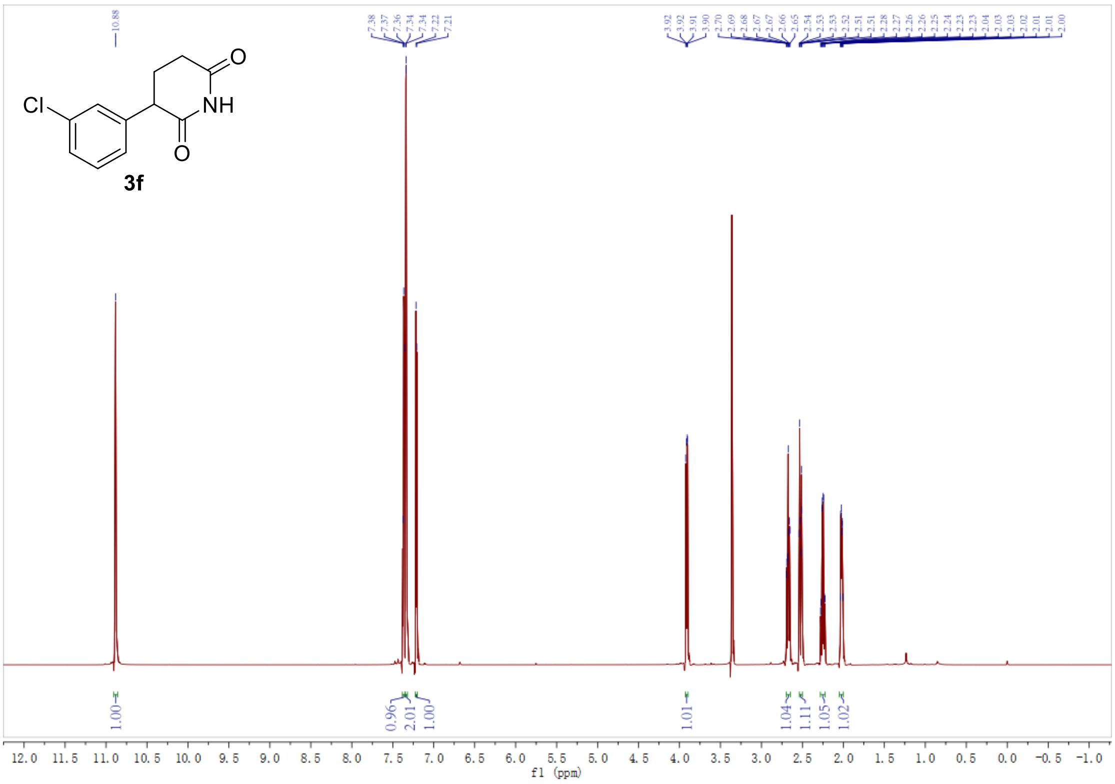

^{13}C NMR spectrum of 3-(2-chlorophenyl)piperidine-2,6-dione (3f). (DMSO-d_6, 200 MHz)

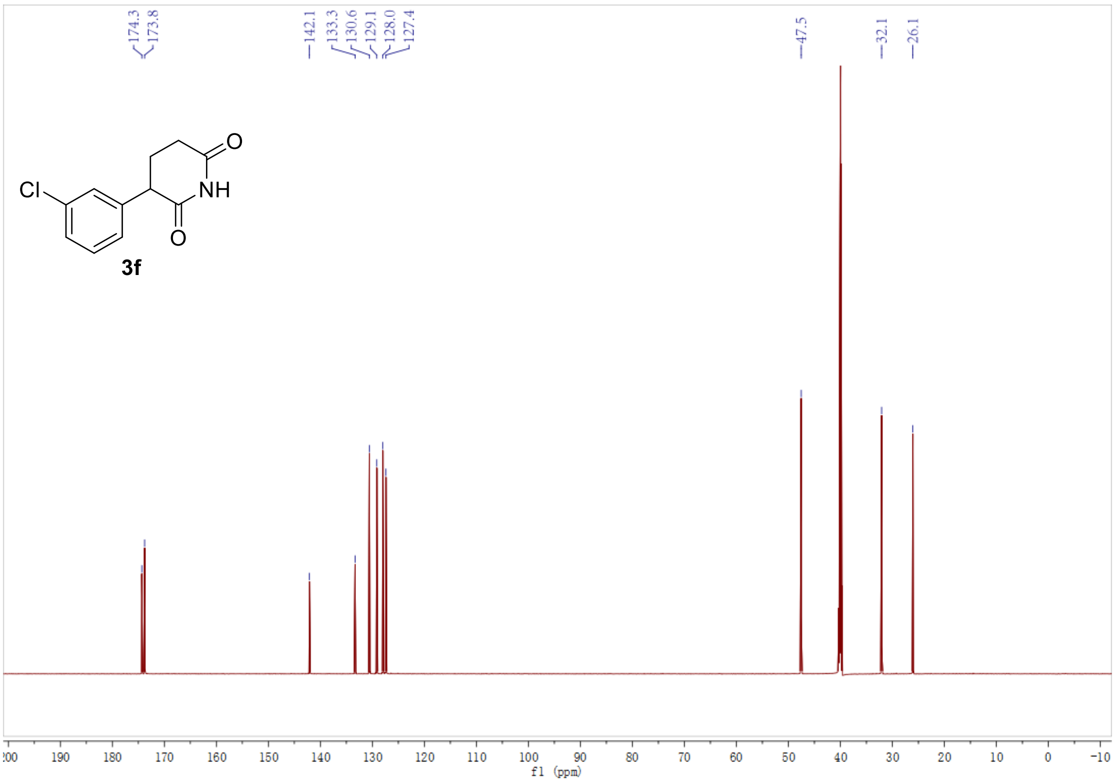

^{1}H NMR spectrum of 3-(4-chlorophenyl)piperidine-2,6-dione (3g). (DMSO-d_6, 800 MHz)

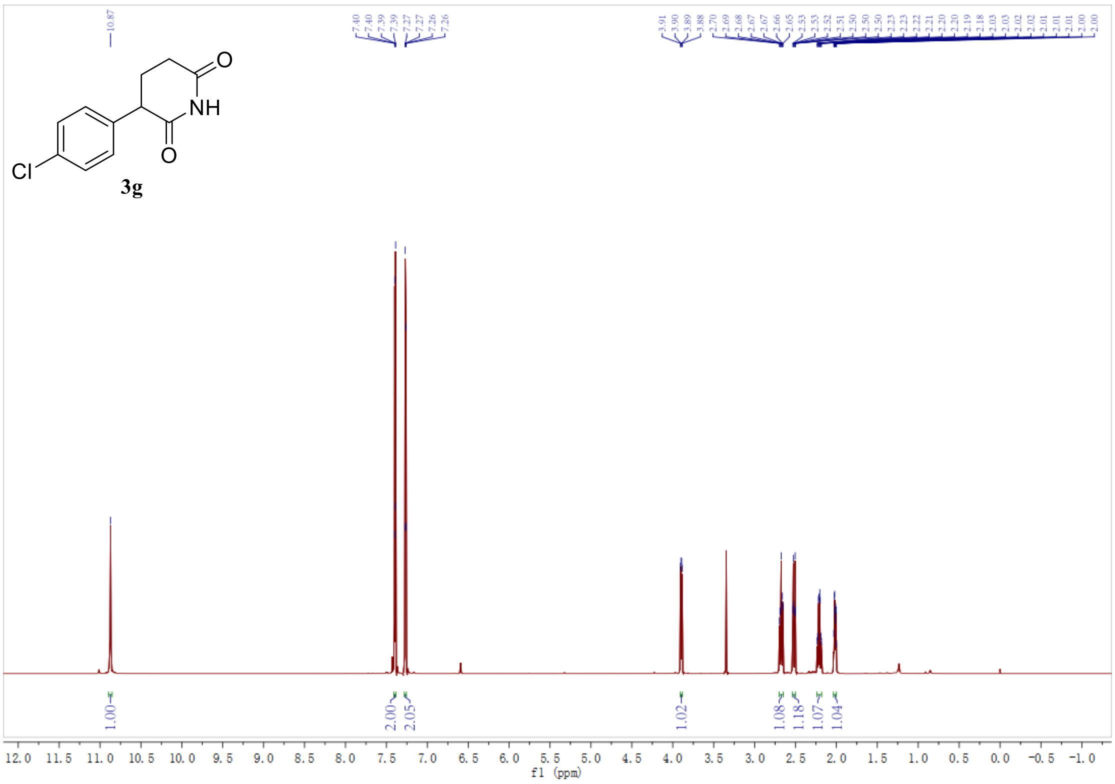

^{13}C NMR spectrum of 3-(4-chlorophenyl)piperidine-2,6-dione (3g). (DMSO-d_6, 200 MHz)

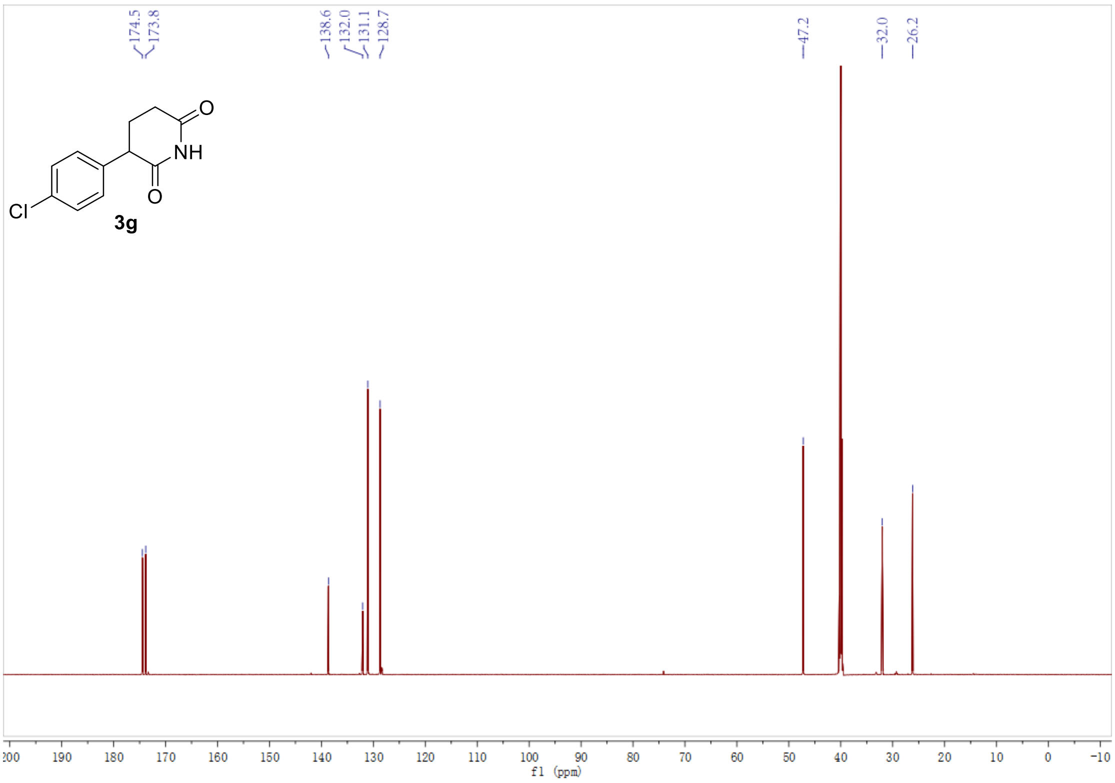

^{1}H NMR spectrum of 3-(2-fluorophenyl)piperidine-2,6-dione (3h). (DMSO-d_6, 800 MHz)

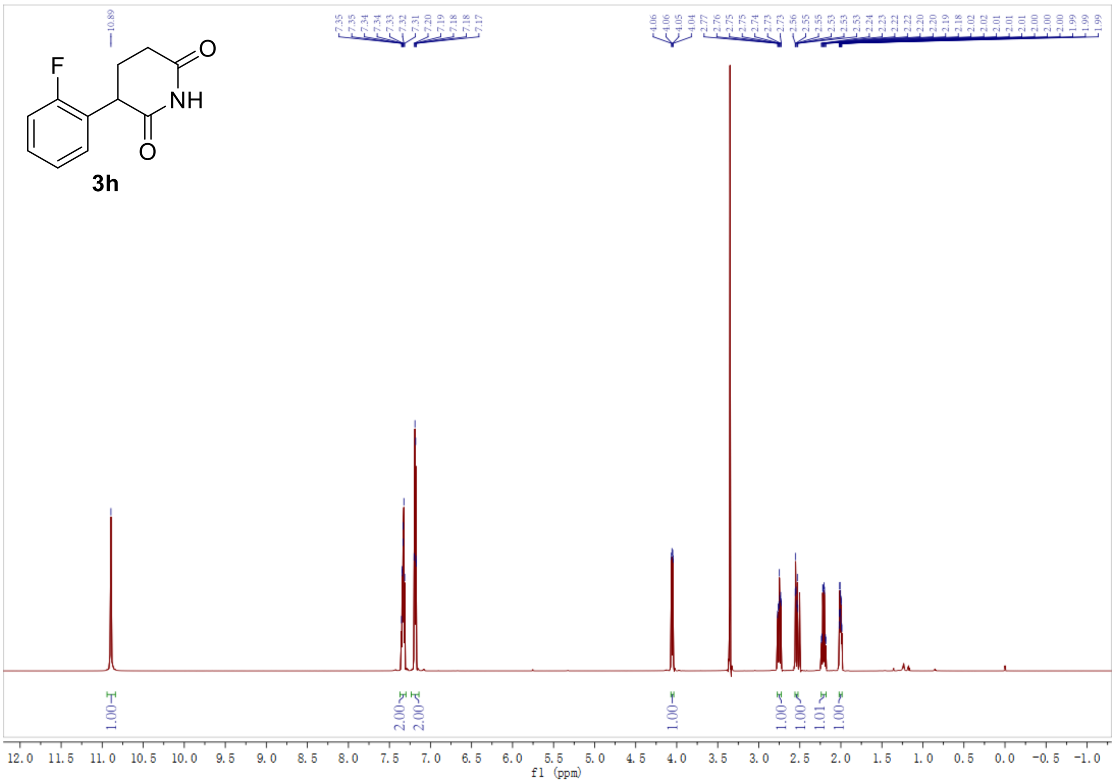

^{13}C NMR spectrum of 3-(2-fluorophenyl)piperidine-2,6-dione (3h). (DMSO-d_6, 200 MHz)

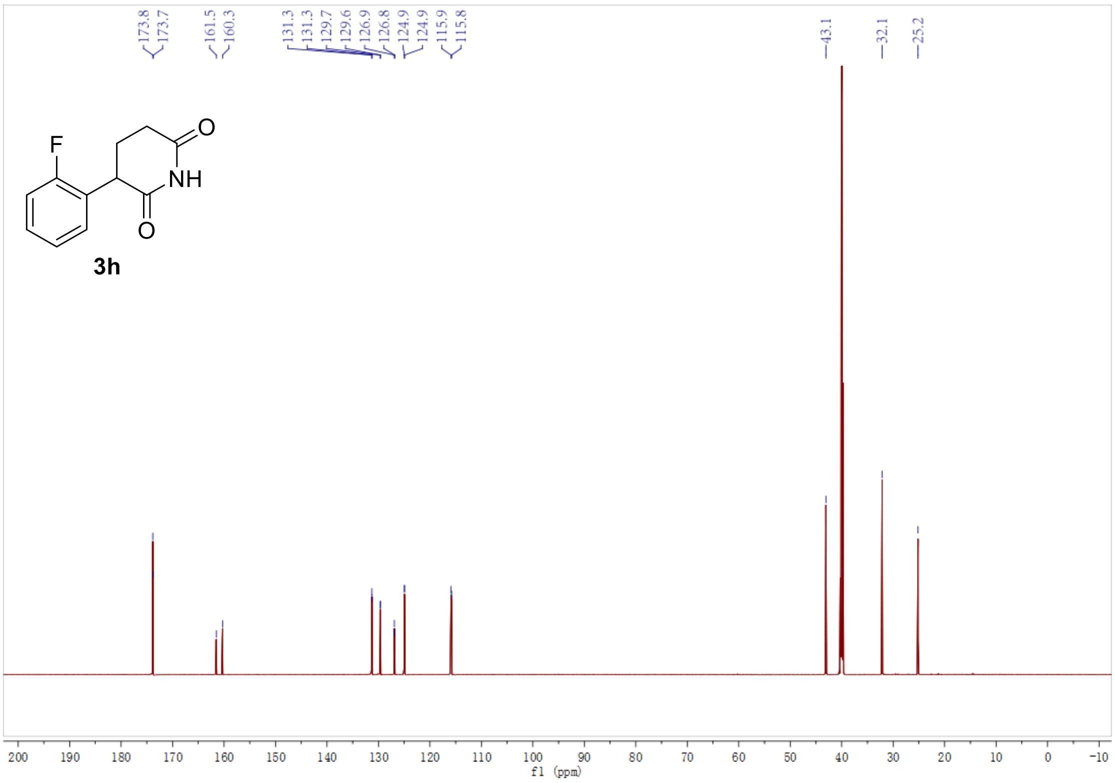

^{19}F NMR spectrum of 3-(2-fluorophenyl)piperidine-2,6-dione (3h). (DMSO-d_6, 753 MHz)

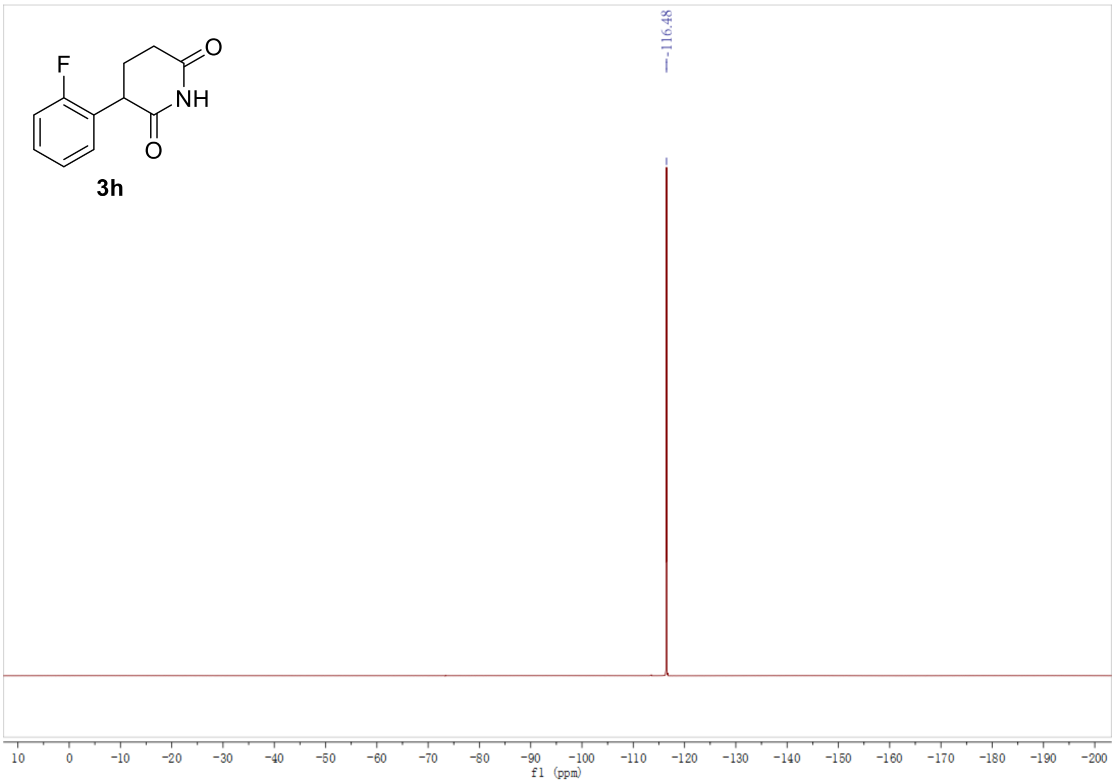

^{1}H NMR spectrum of 3-(3-fluorophenyl)piperidine-2,6-dione (3i). (DMSO-d_6, 400 MHz)

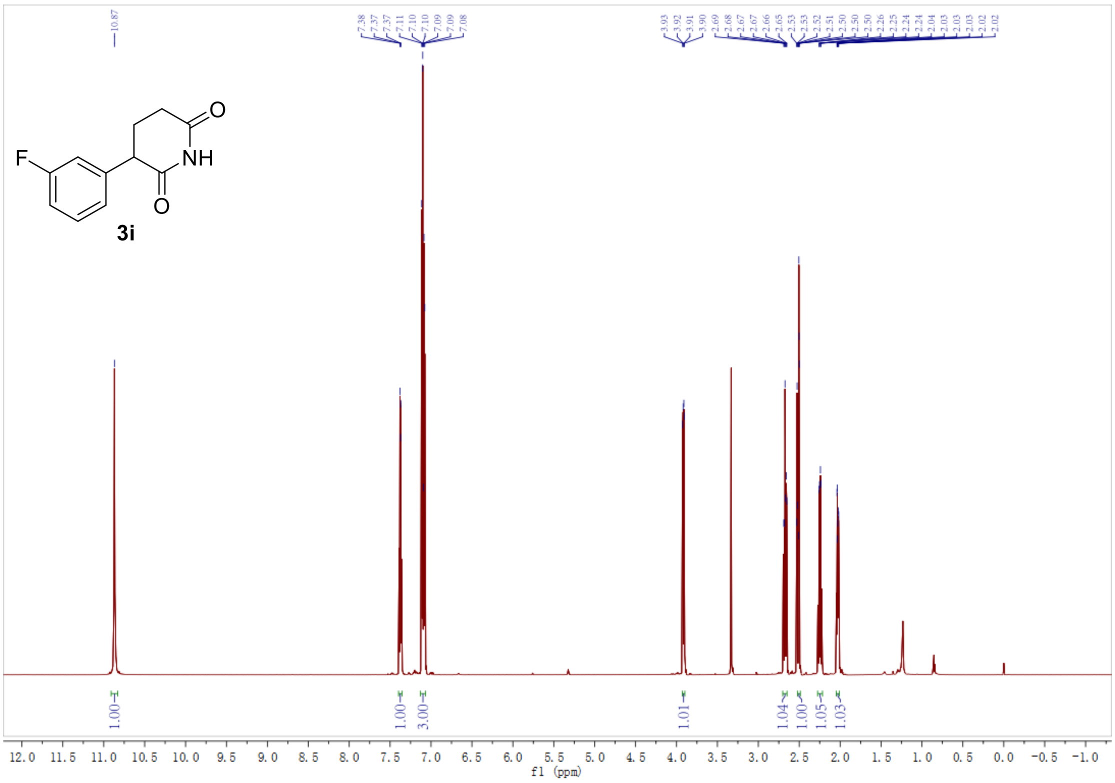

^{13}C NMR spectrum of 3-(3-fluorophenyl)piperidine-2,6-dione (3i). (DMSO-d_6, 100 MHz)

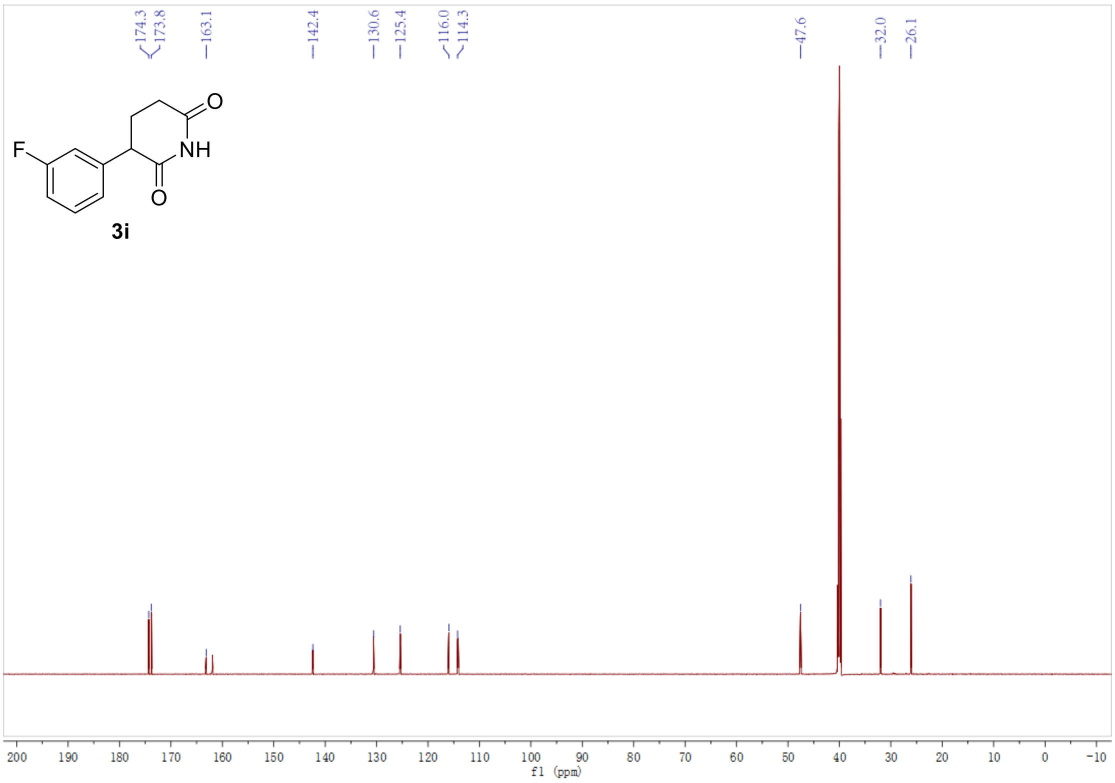

^{19}F NMR spectrum of 3-(3-fluorophenyl)piperidine-2,6-dione (3i). (DMSO-d_6, 753 MHz)

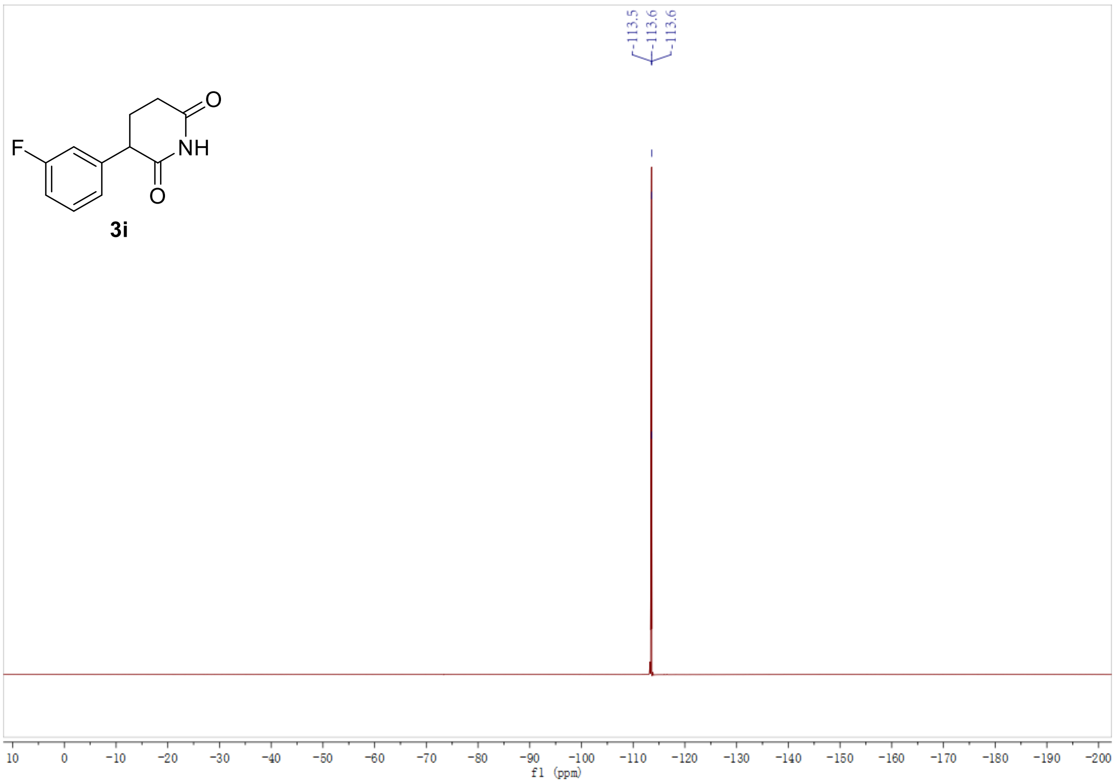

^{1}H NMR spectrum of 3-(4-fluorophenyl)piperidine-2,6-dione (3j). (DMSO-d_6, 800 MHz)

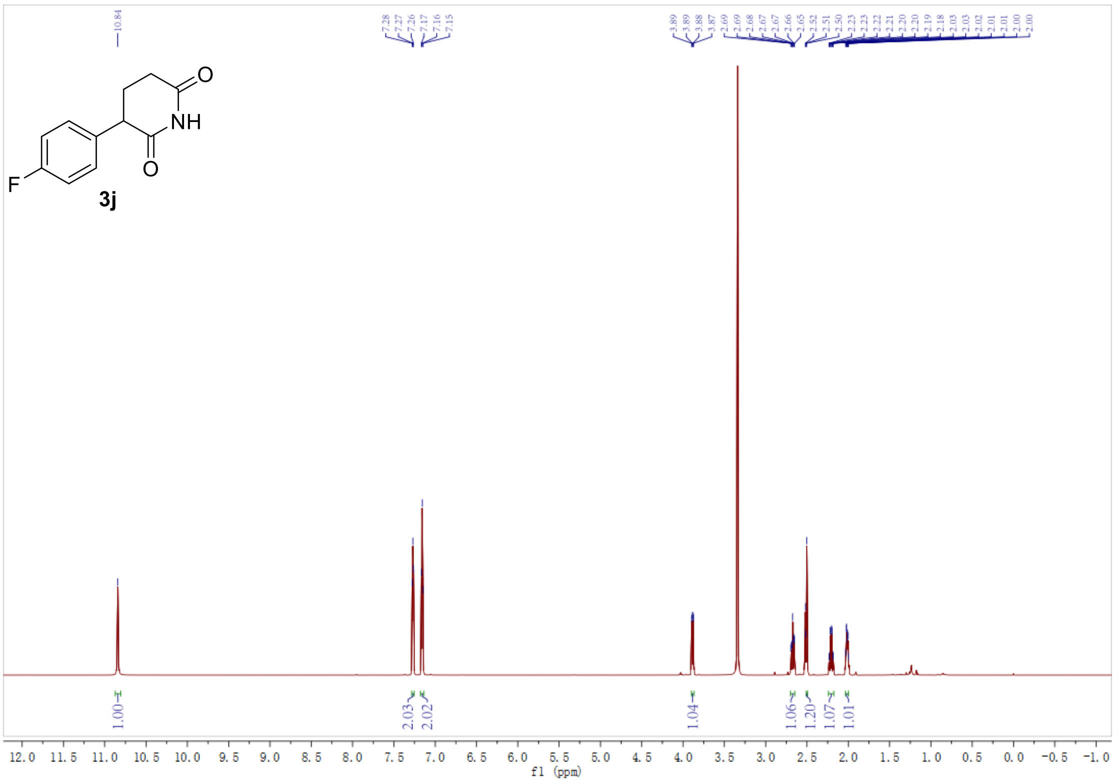

^{13}C NMR spectrum of 3-(4-fluorophenyl)piperidine-2,6-dione (3j). (DMSO-d_6, 200 MHz)

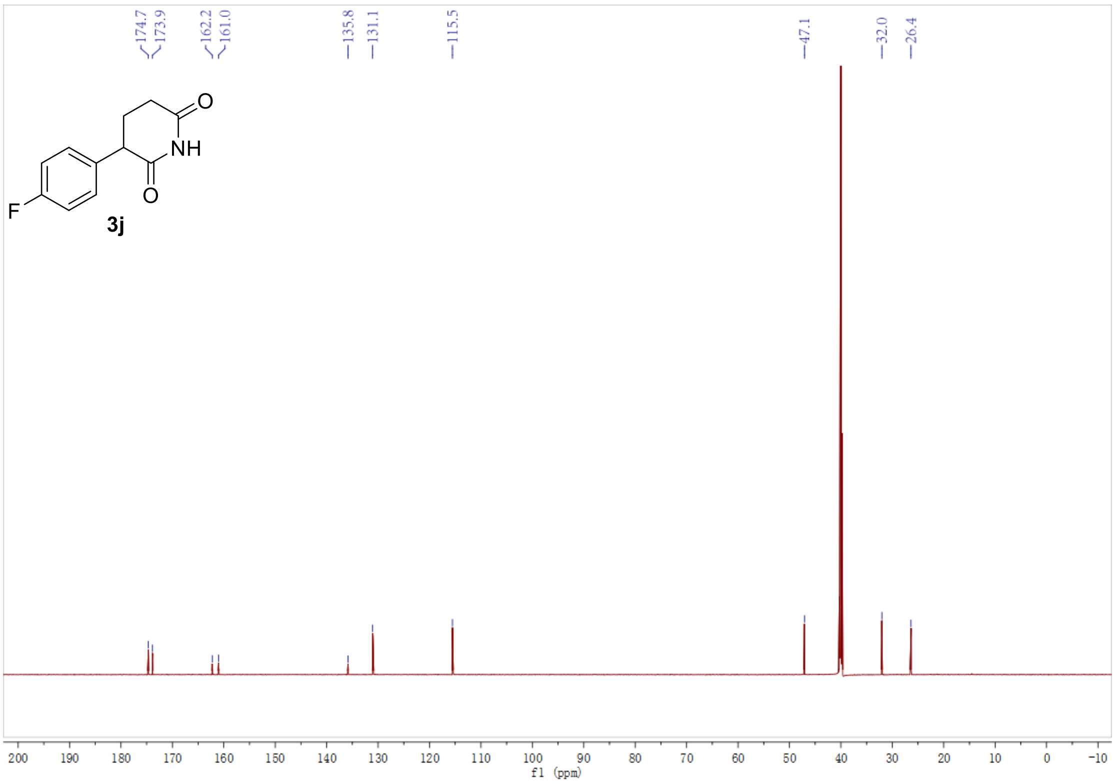

^{19}F NMR spectrum of 3-(4-fluorophenyl)piperidine-2,6-dione (3j). (DMSO-d_6, 753 MHz)

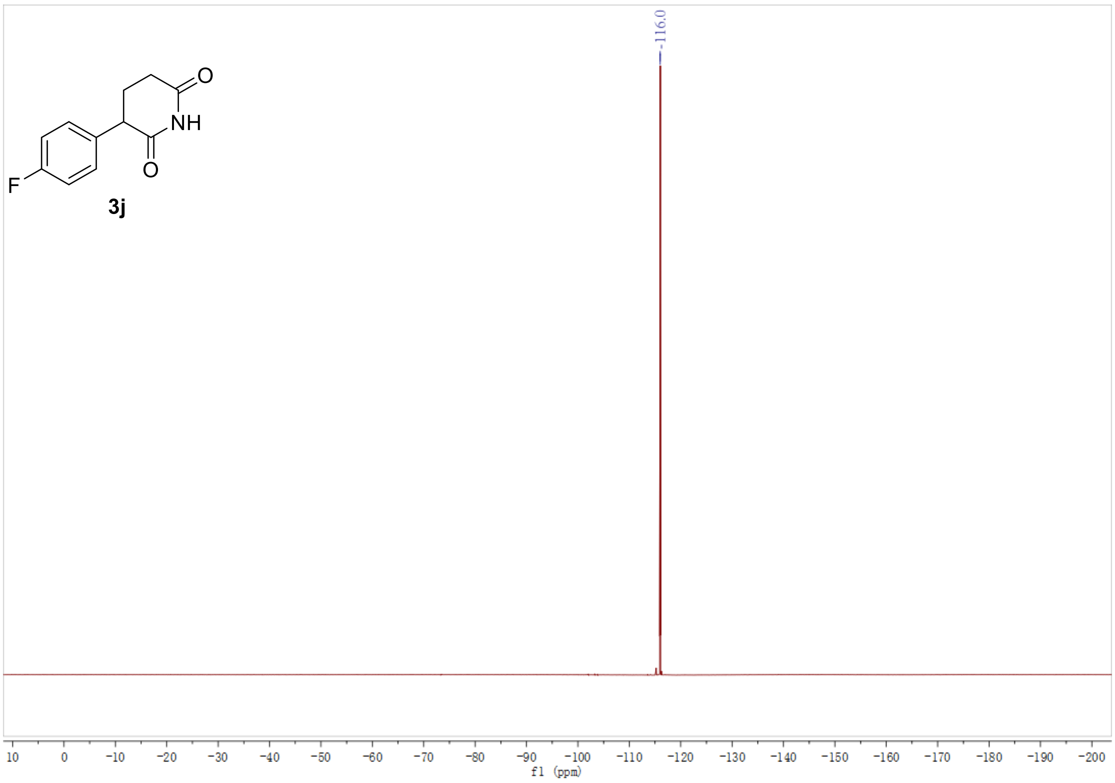

^{1}H NMR spectrum of 3-(4-nitrophenyl)piperidine-2,6-dione (3k). (DMSO-d_6, 800 MHz)

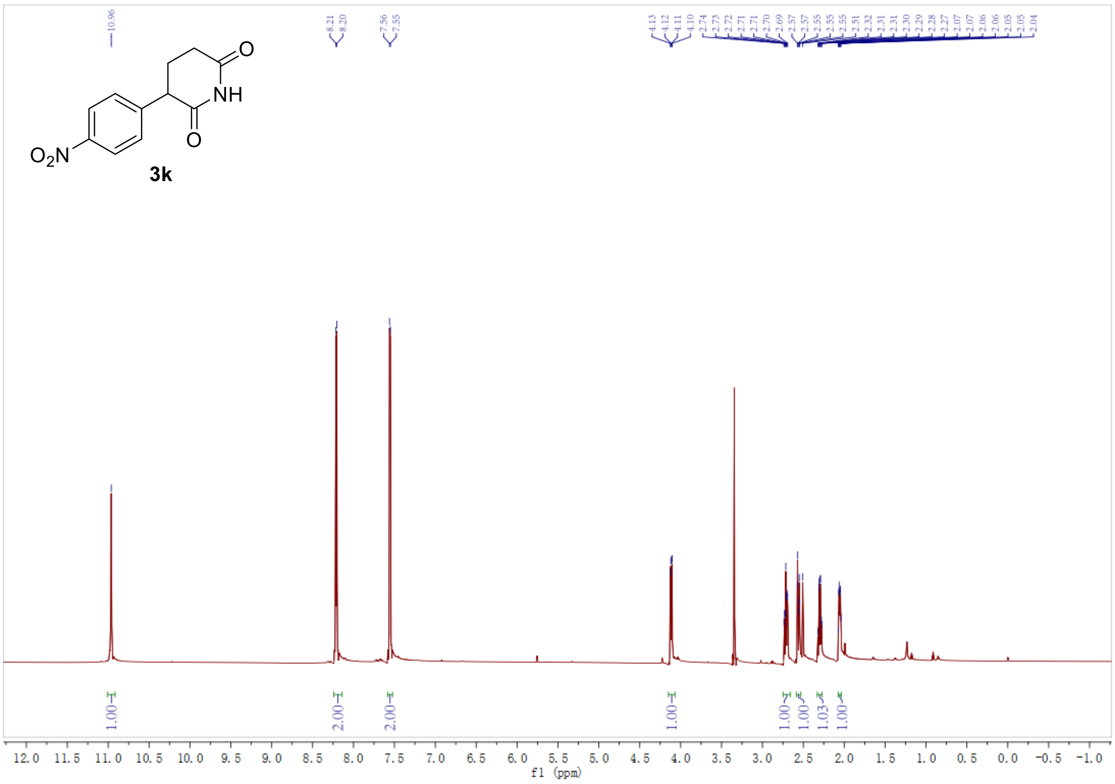

^{13}C NMR spectrum of 3-(4-nitrophenyl)piperidine-2,6-dione (3k). (DMSO-d_6, 200 MHz)

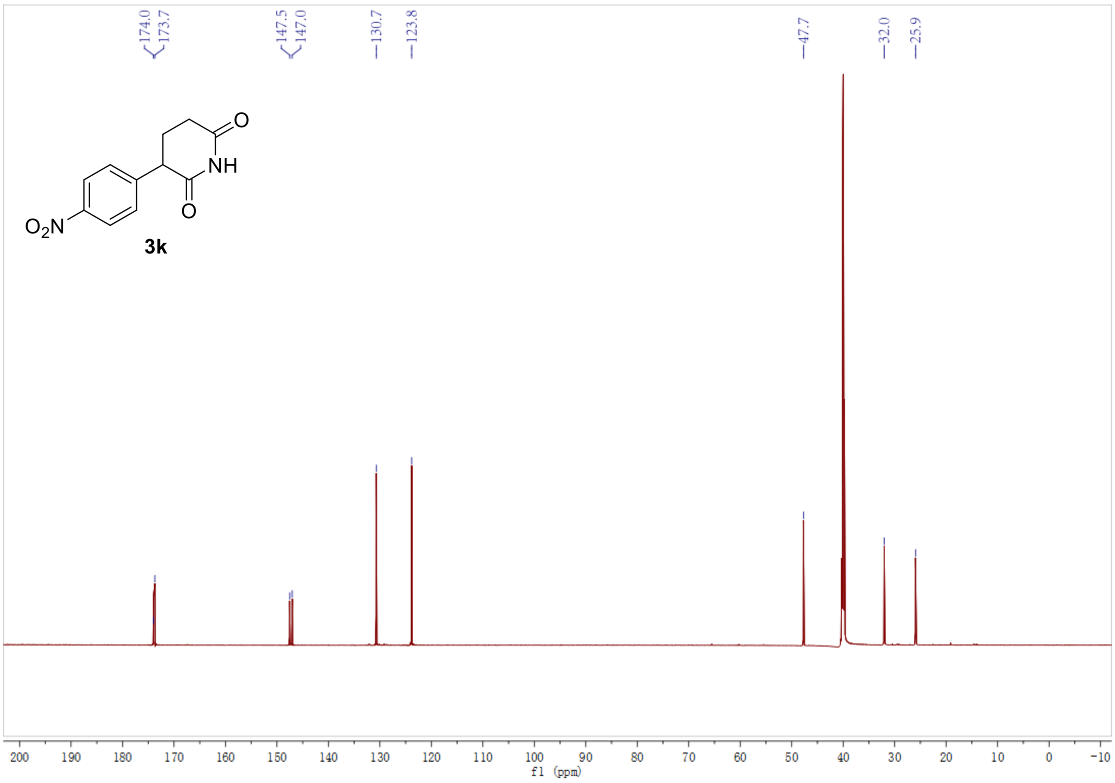

^{1}H NMR spectrum of 3-(3-nitrophenyl)piperidine-2,6-dione (3l). (DMSO-d_6, 800 MHz)

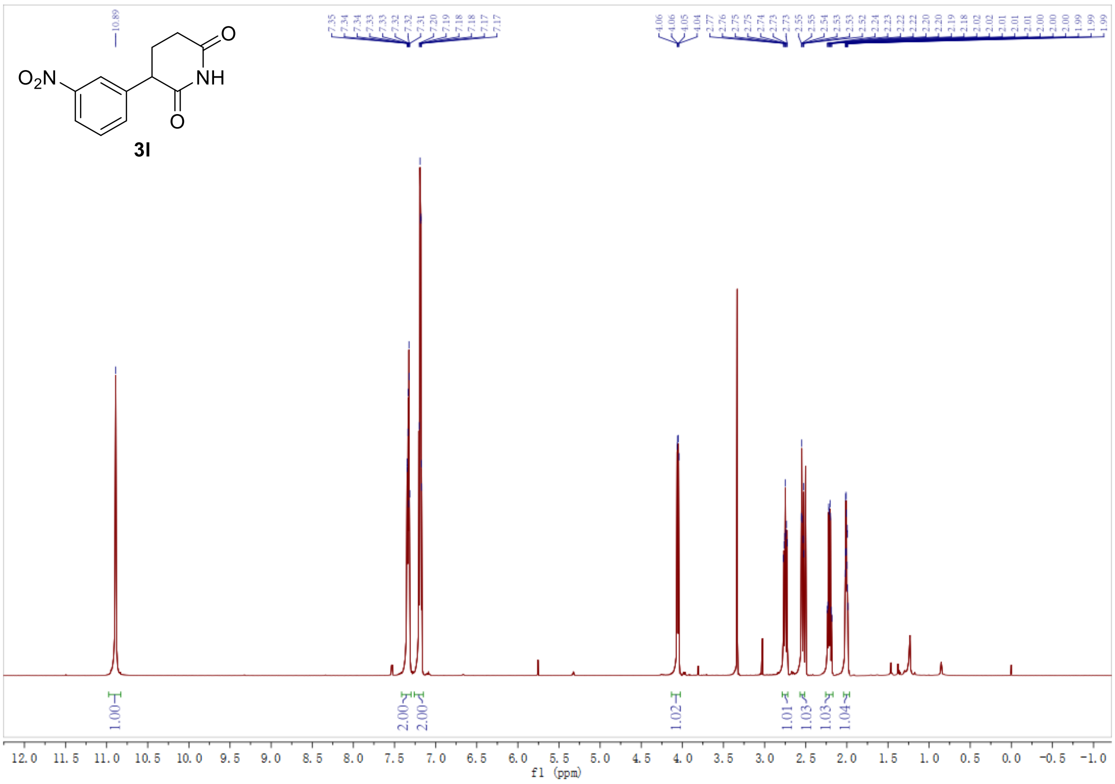

^{13}C NMR spectrum of 3-(3-nitrophenyl)piperidine-2,6-dione (3l). (DMSO-d_6, 200 MHz)

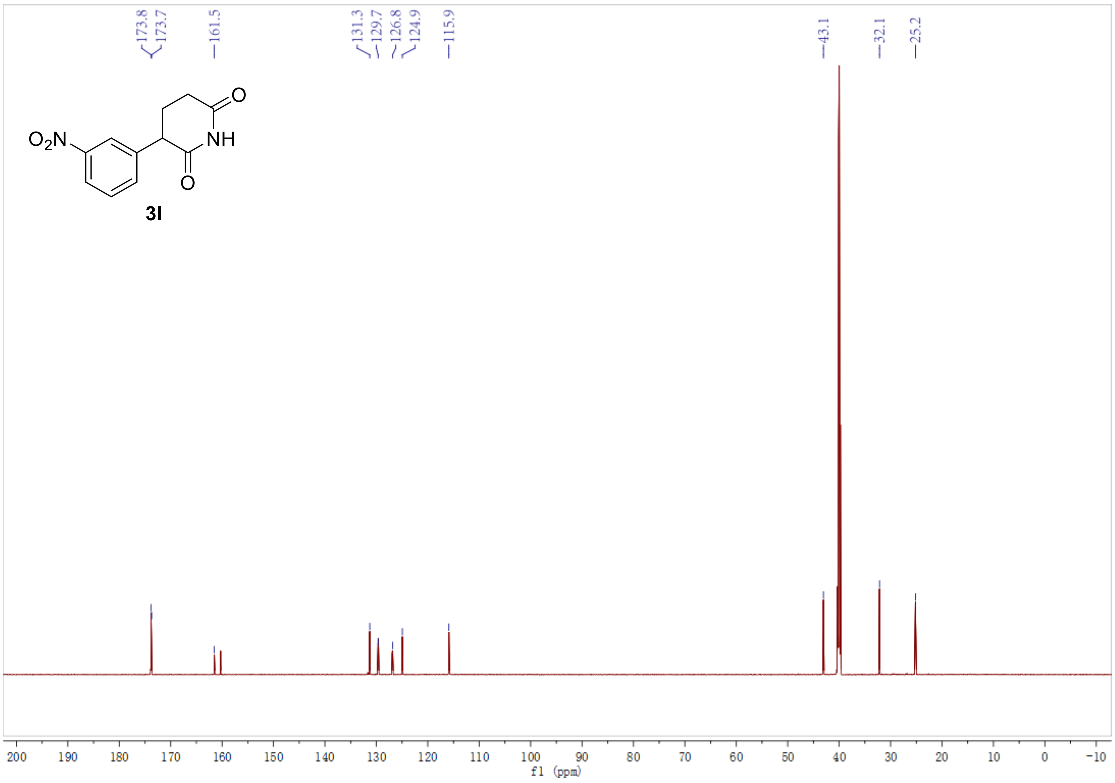

^1H NMR spectrum of 3-(2-methoxyphenyl)piperidine-2,6-dione (3m). (DMSO-d_6, 400 MHz)

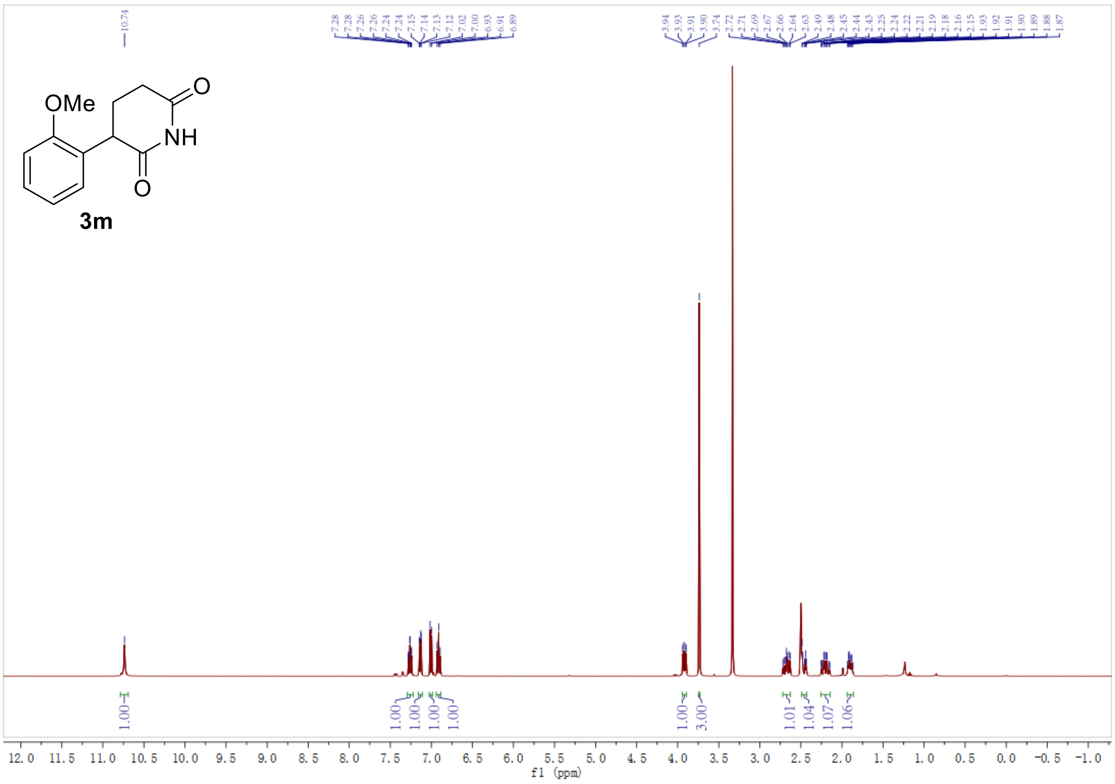

^{13}C NMR spectrum of 3-(2-methoxyphenyl)piperidine-2,6-dione (3m). (DMSO-d_6, 100 MHz)

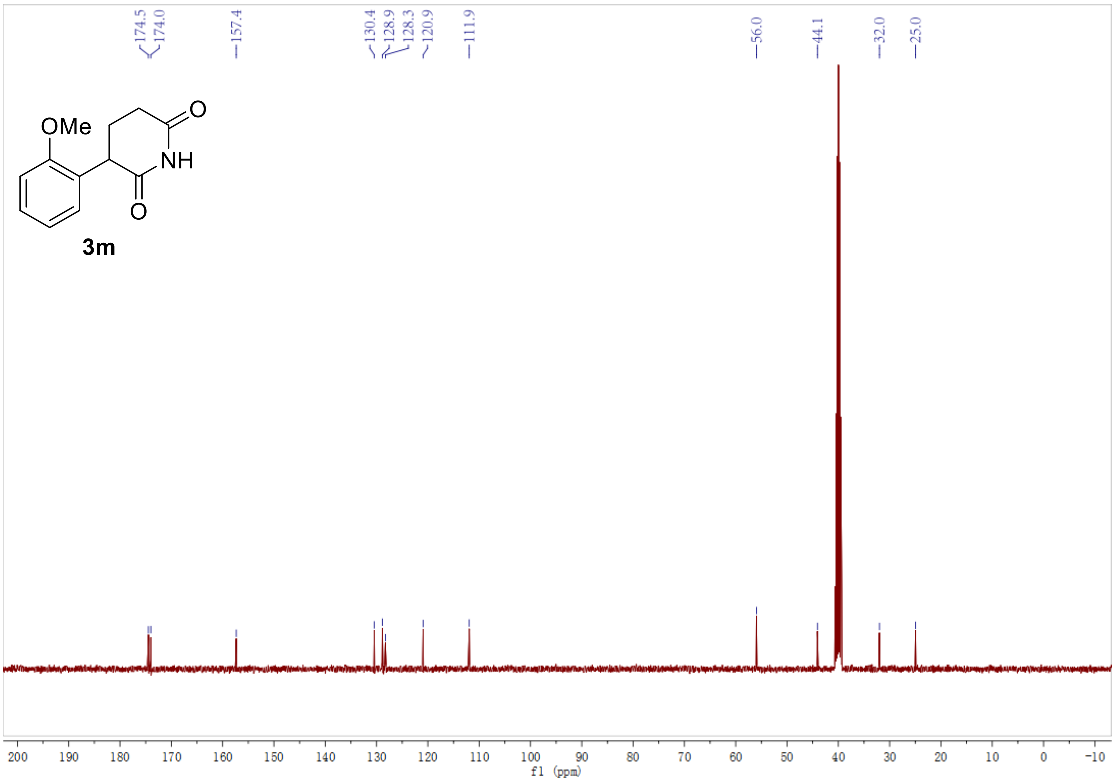

^{1}H NMR spectrum of 3-(3-methoxyphenyl)piperidine-2,6-dione (3n). (DMSO-d_6, 800 MHz)

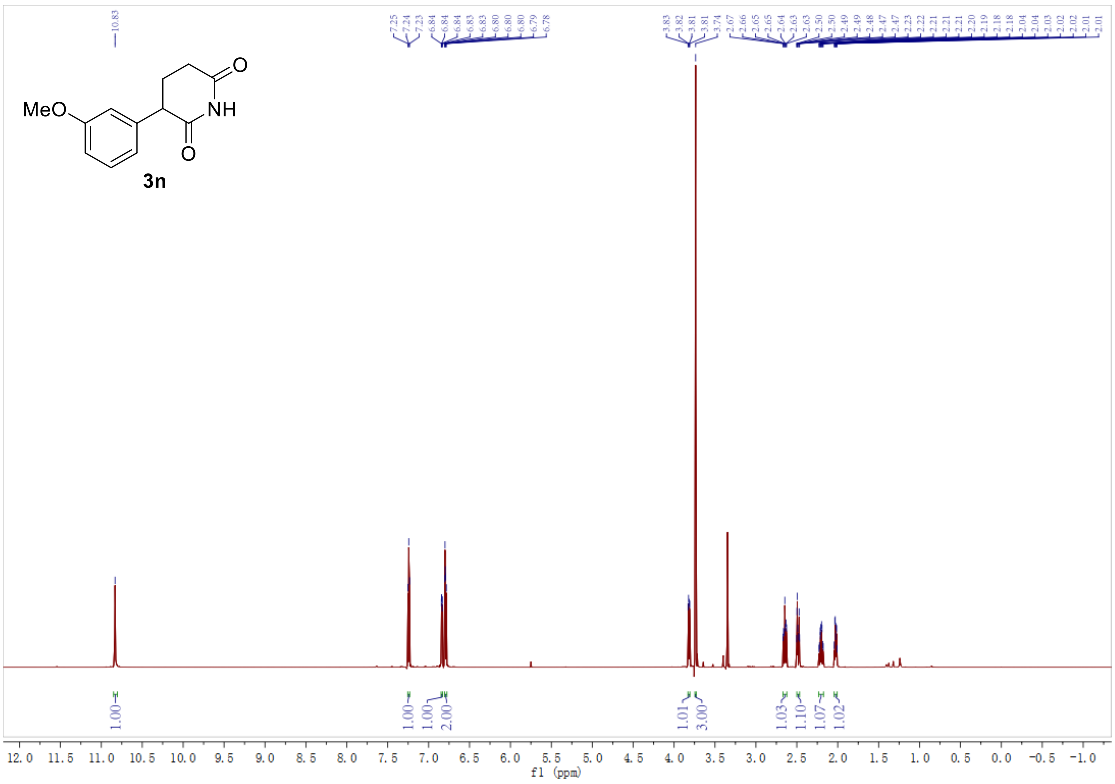

^{13}C NMR spectrum of 3-(3-methoxyphenyl)piperidine-2,6-dione (3n). (DMSO-d_6, 200 MHz)

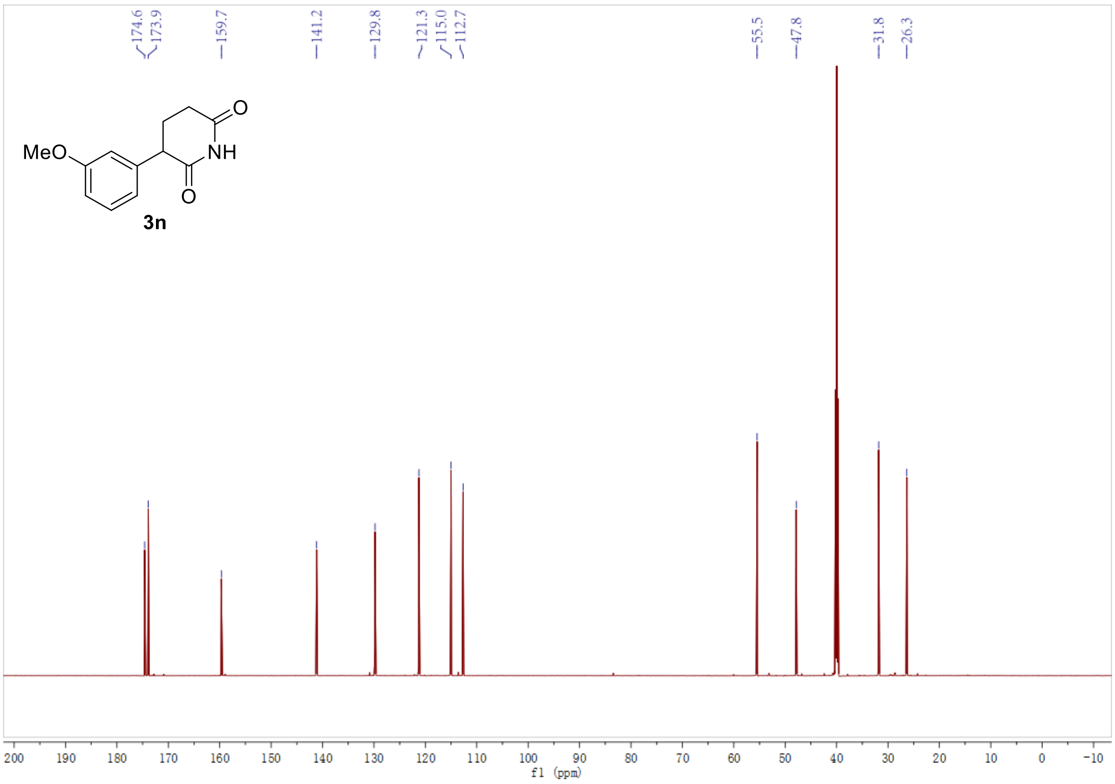

^{1}H NMR spectrum of 3-(4-methoxyphenyl)piperidine-2,6-dione (3o). (DMSO-d_6, 800 MHz)

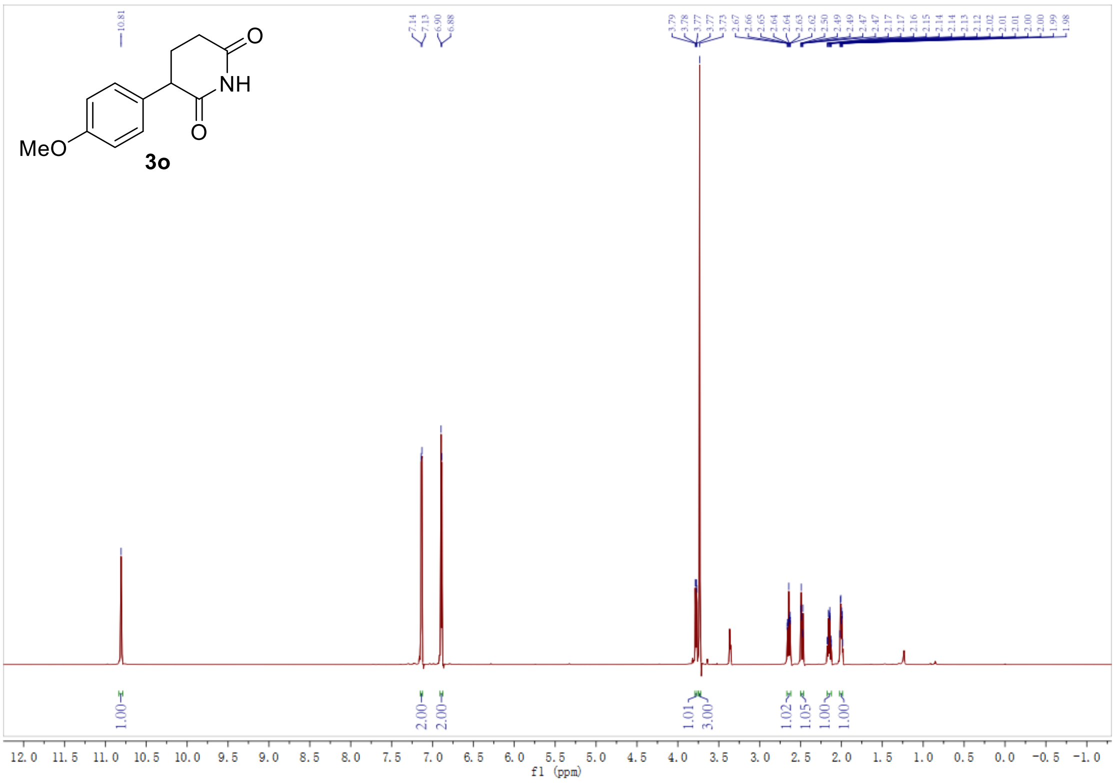

^{13}C NMR spectrum of 3-(4-methoxyphenyl)piperidine-2,6-dione (3o). (DMSO-d_6, 200 MHz)

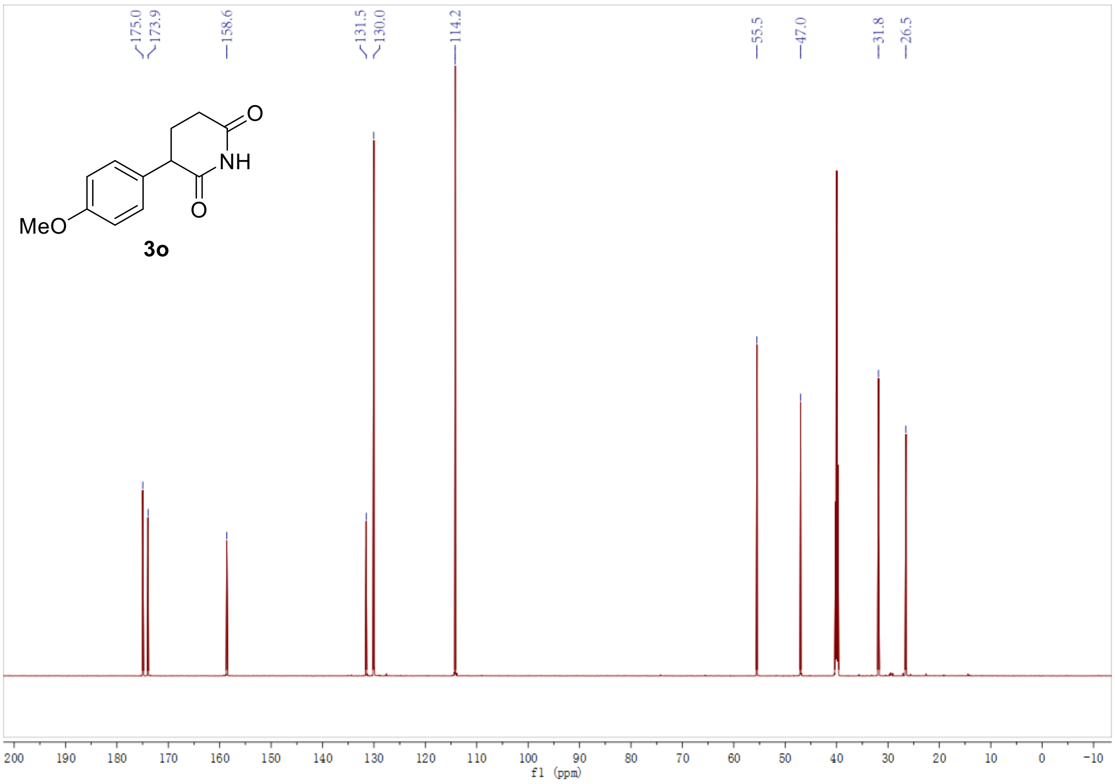

^{1}H NMR spectrum of 3-(4-ethoxyphenyl)piperidine-2,6-dione (3p). (DMSO-d_6, 800 MHz)

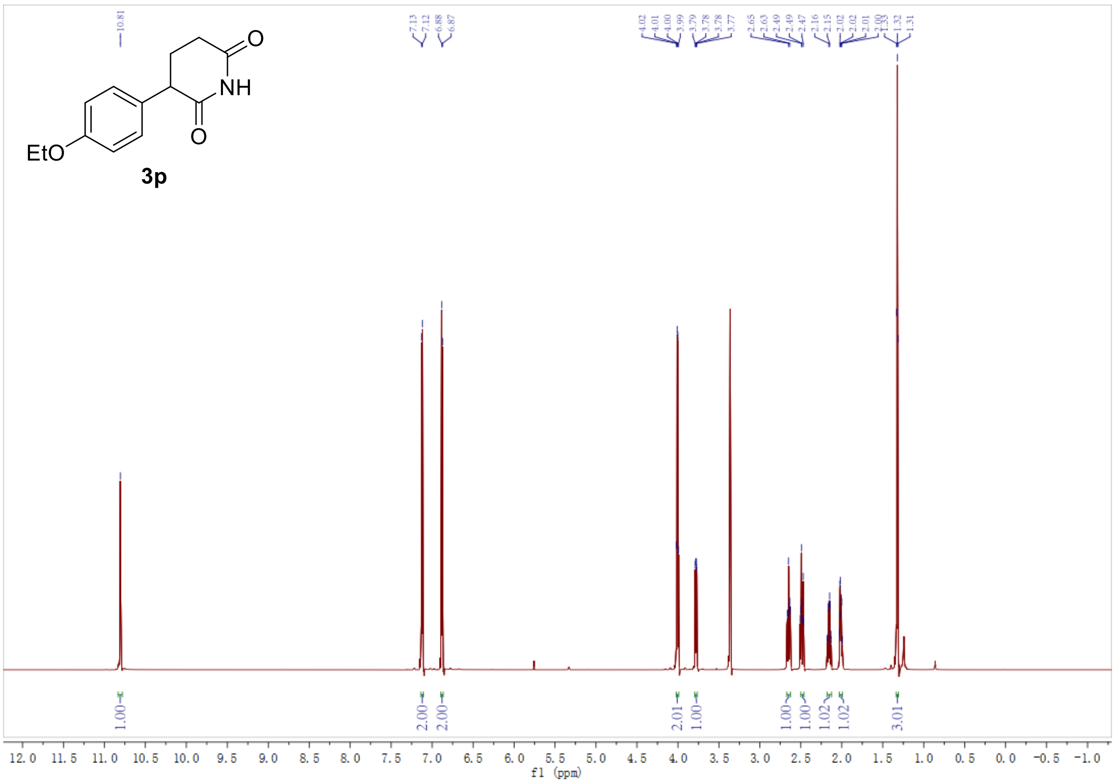

^{13}C NMR spectrum of 3-(4-ethoxyphenyl)piperidine-2,6-dione (3p). (DMSO-d_6, 200 MHz)

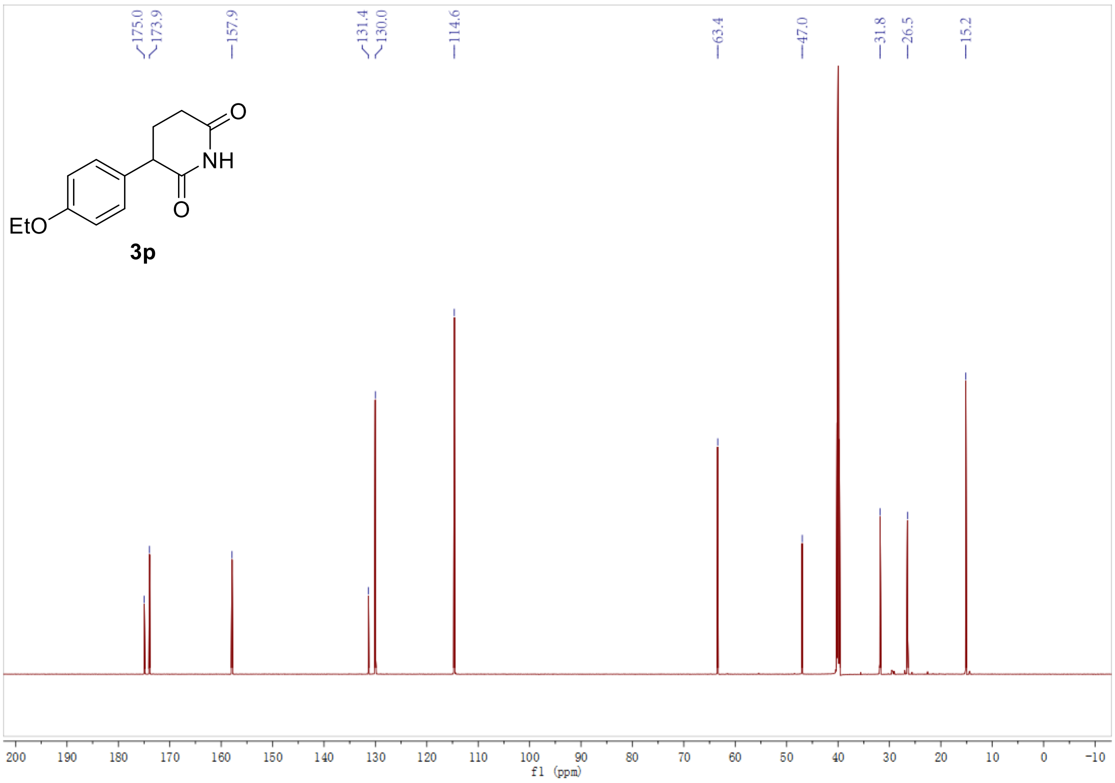

^{1}H NMR spectrum of 3-(*o*-tolyl)piperidine-2,6-dione (3q). (DMSO-d_6, 800 MHz)

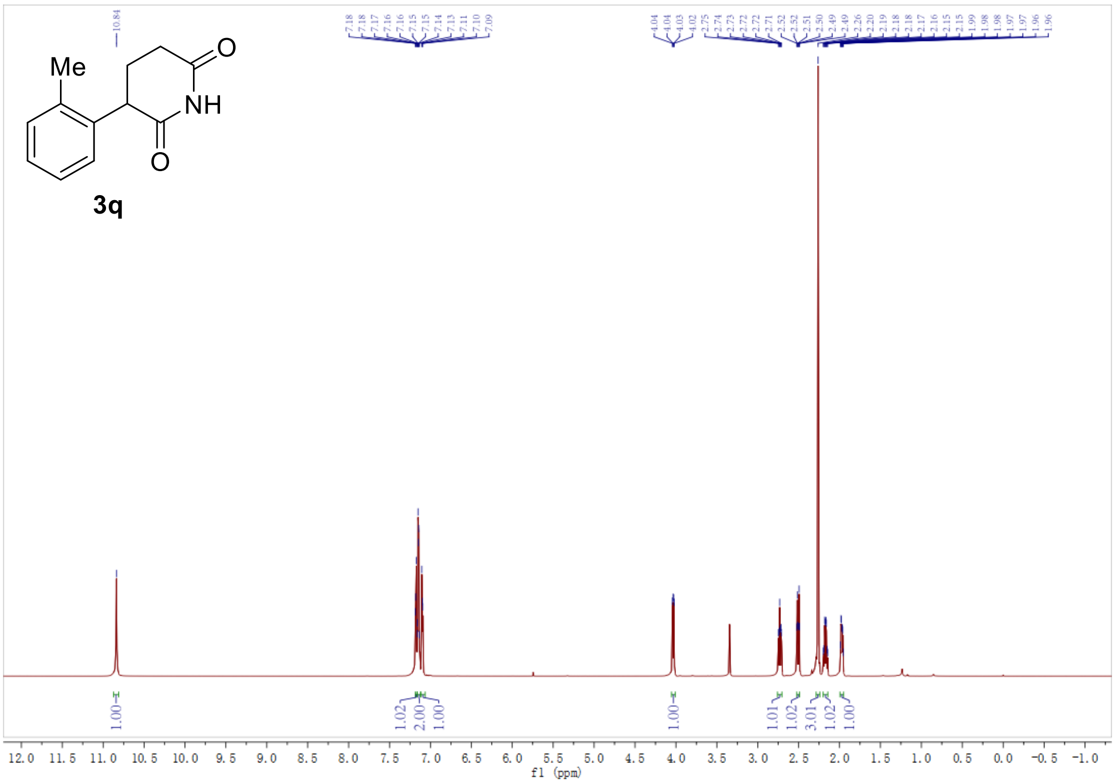

^{13}C NMR spectrum of 3-(*o*-tolyl)piperidine-2,6-dione (3q). (DMSO-d_6, 200 MHz)

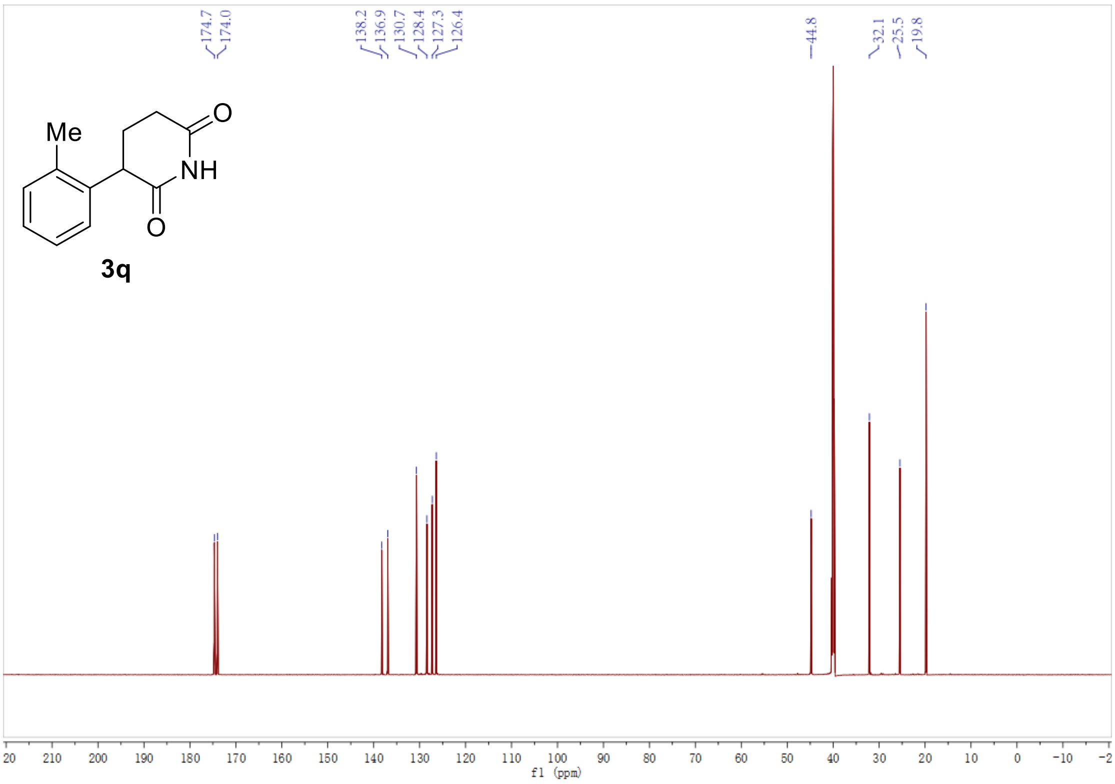

^{1}H NMR spectrum of 3-(*m*-tolyl)piperidine-2,6-dione (3r). (DMSO-d_6, 800 MHz)

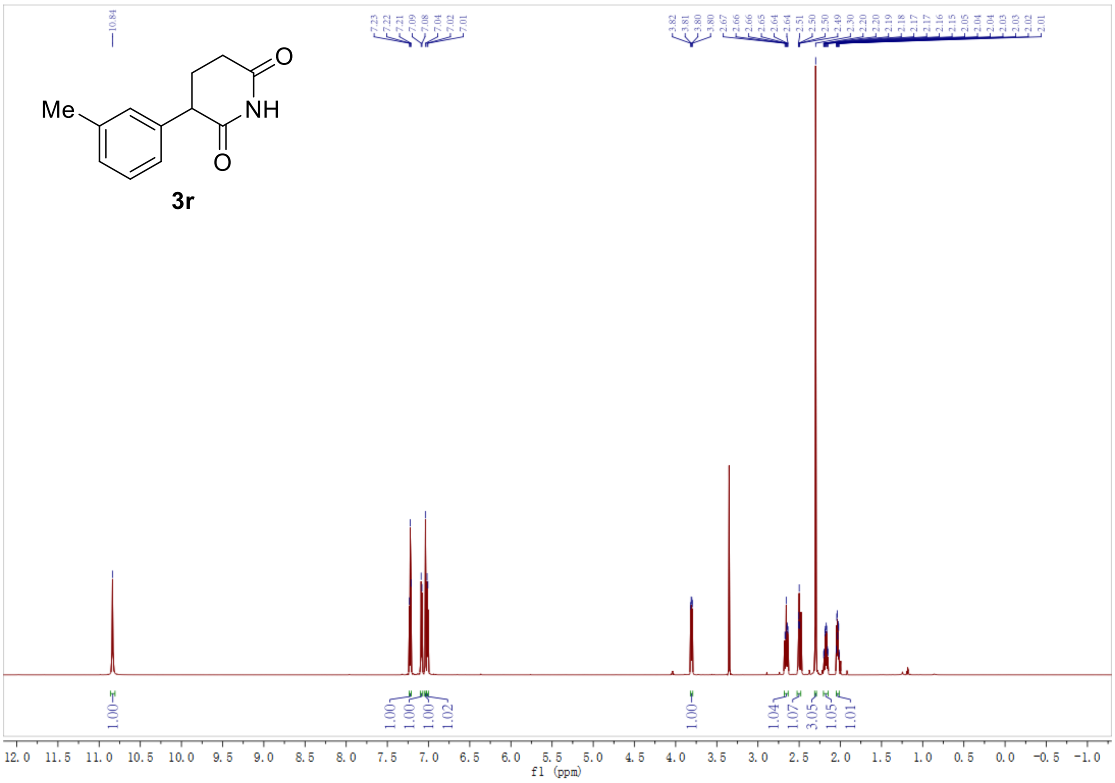

^{13}C NMR spectrum of 3-(*m*-tolyl)piperidine-2,6-dione (3r). (DMSO-d_6, 200 MHz)

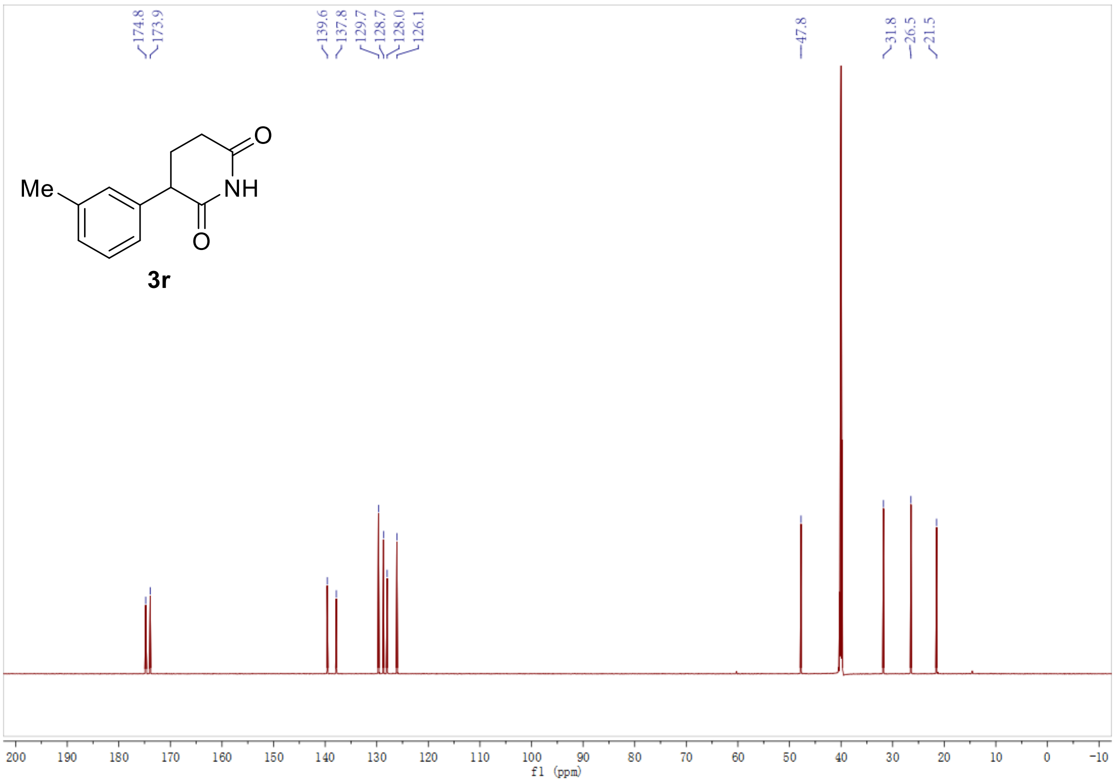

^{1}H NMR spectrum of 3-(*p*-tolyl)piperidine-2,6-dione (3s). (DMSO-d_6, 800 MHz)

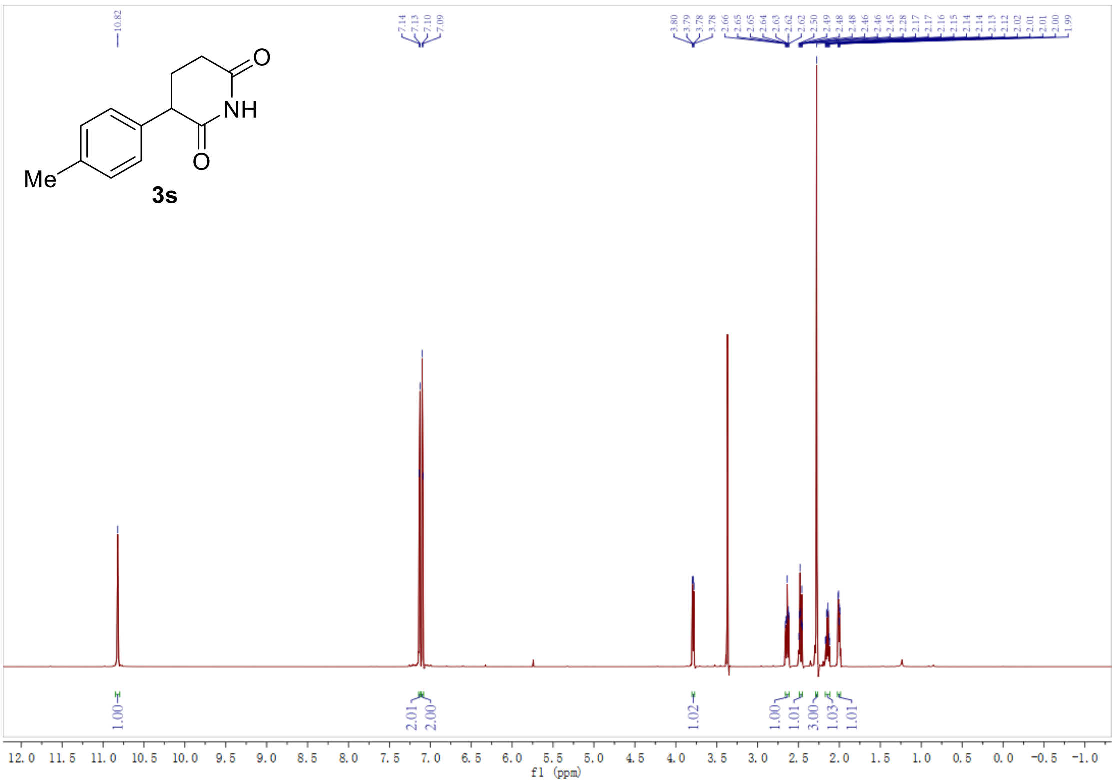

^{13}C NMR spectrum of 3-(*p*-tolyl)piperidine-2,6-dione (3s). (DMSO-d_6, 200 MHz)

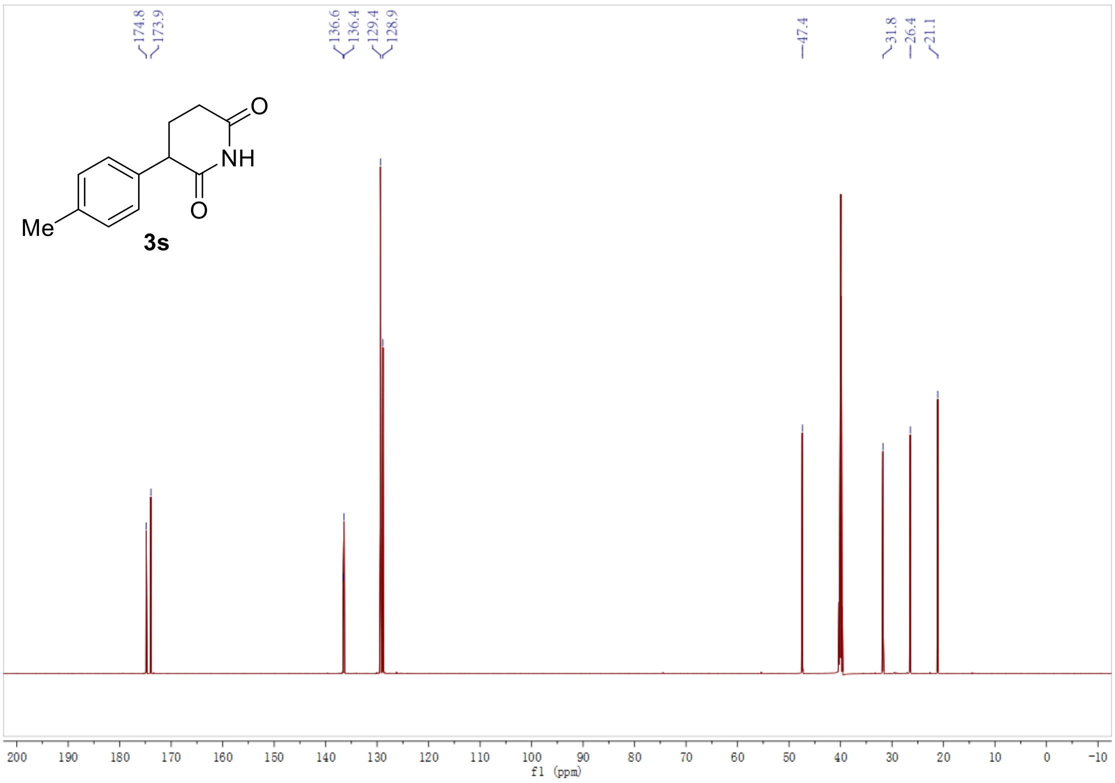

^{1}H NMR spectrum of *tert*-butyl (4-(2,6-dioxopiperidin-3-yl)phenyl)carbamate (3t). (DMSO-d_6, 800 MHz)

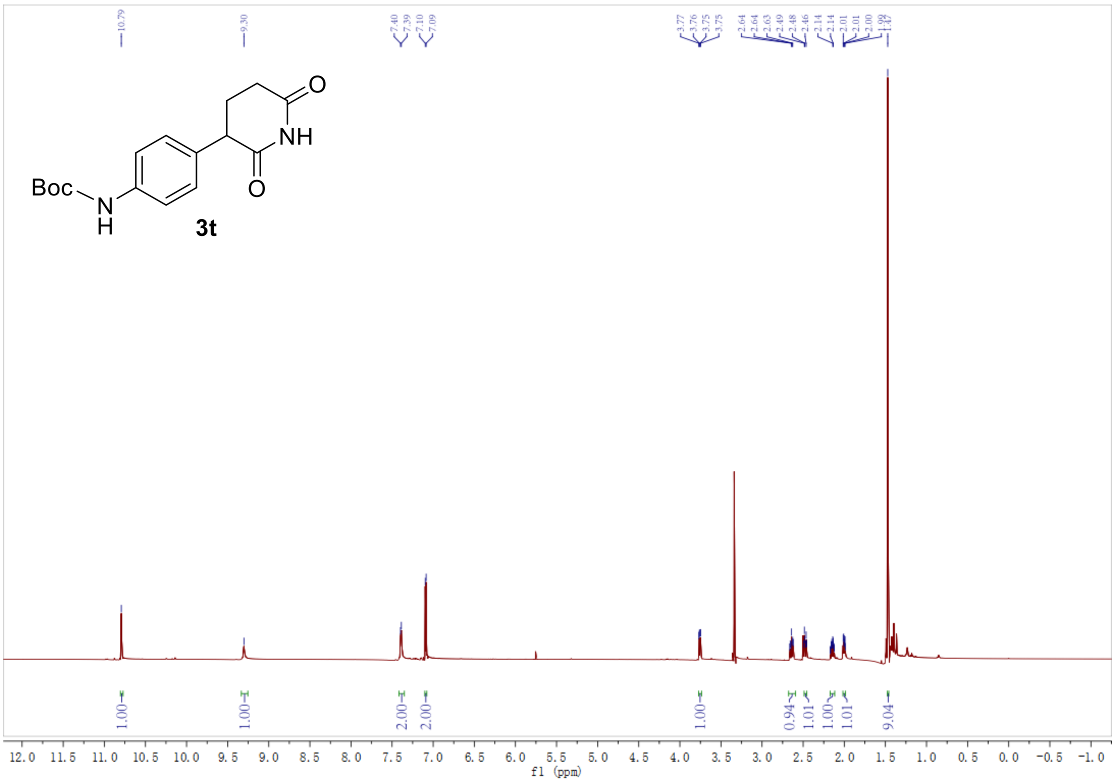

^{13}C NMR spectrum of *tert*-butyl (4-(2,6-dioxopiperidin-3-yl)phenyl)carbamate (3t). (DMSO-d_6, 200 MHz)

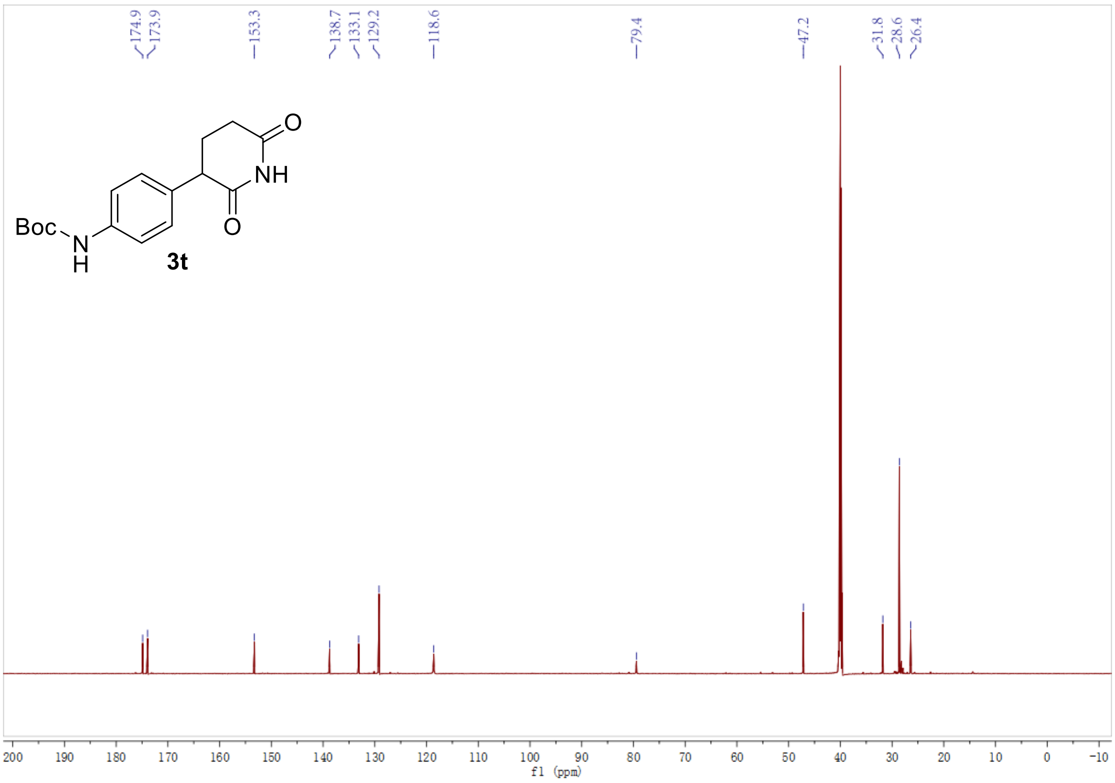

^{1}H NMR spectrum of 3-(4-bromo-3-methylphenyl)piperidine-2,6-dione (3u). (DMSO-d_6, 800

MHz)

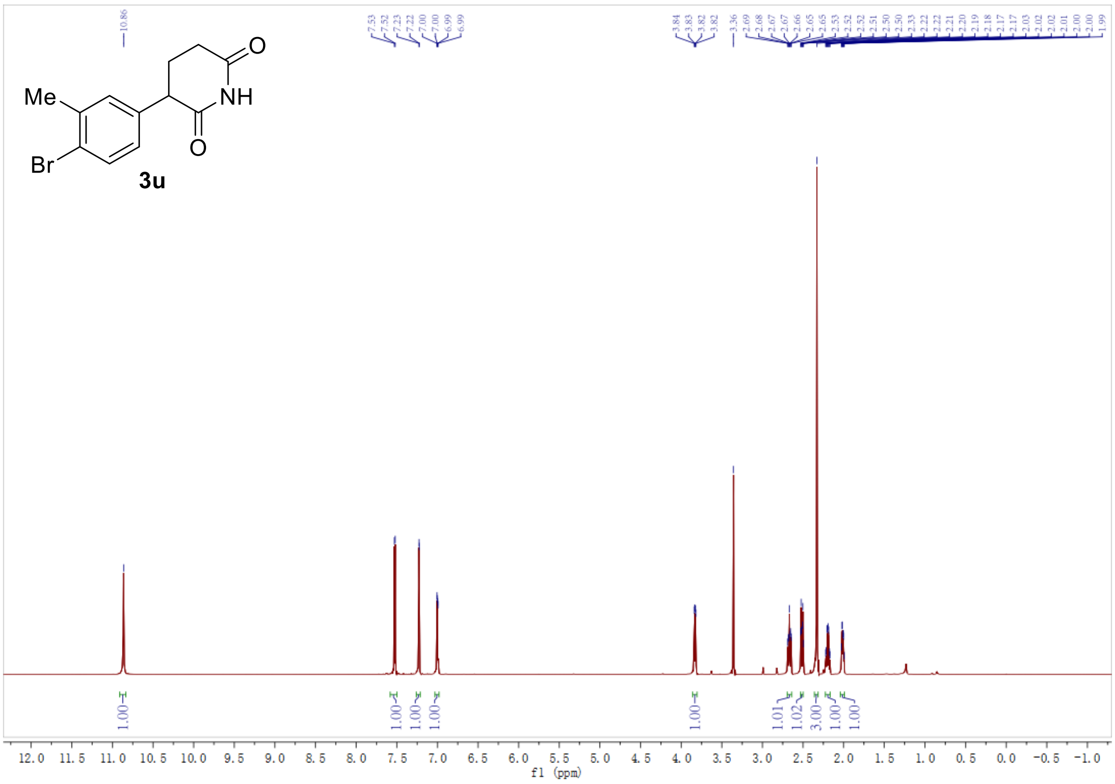

^{13}C NMR spectrum of 3-(4-bromo-3-methylphenyl)piperidine-2,6-dione (3u). (DMSO-d_6, 200 MHz)

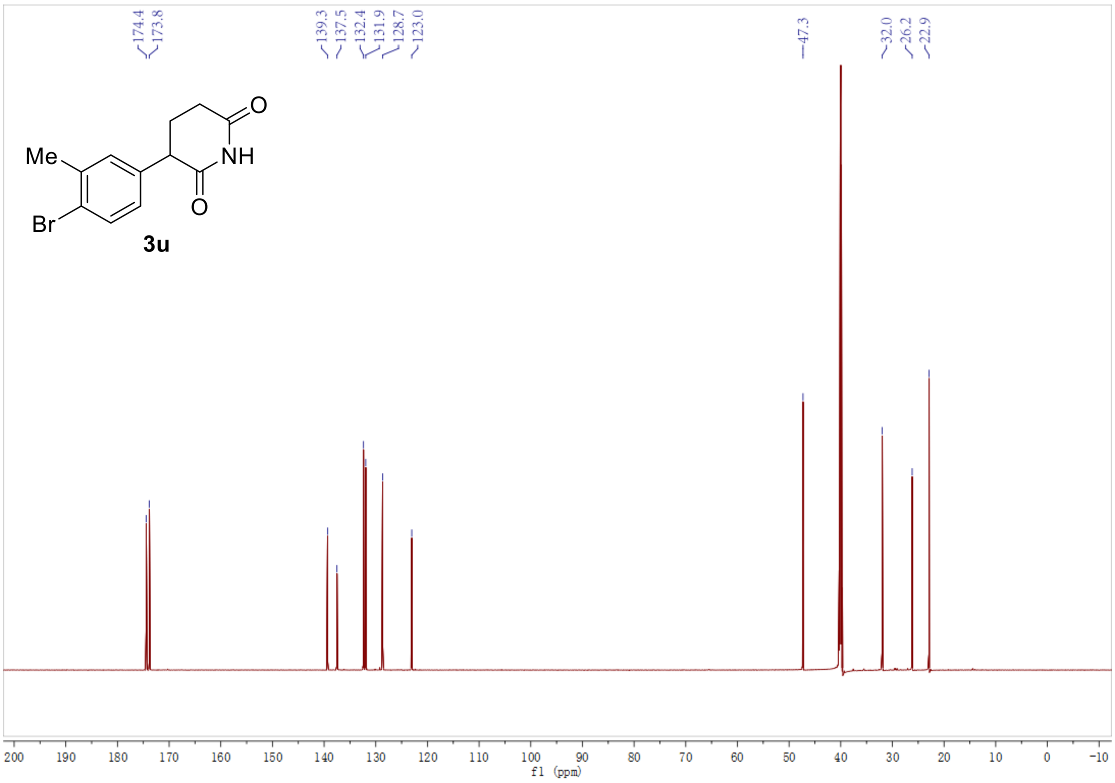

^{1}H NMR spectrum of 3-(4-bromo-2-fluorophenyl)piperidine-2,6-dione (3v). (DMSO-d_6, 400 MHz)

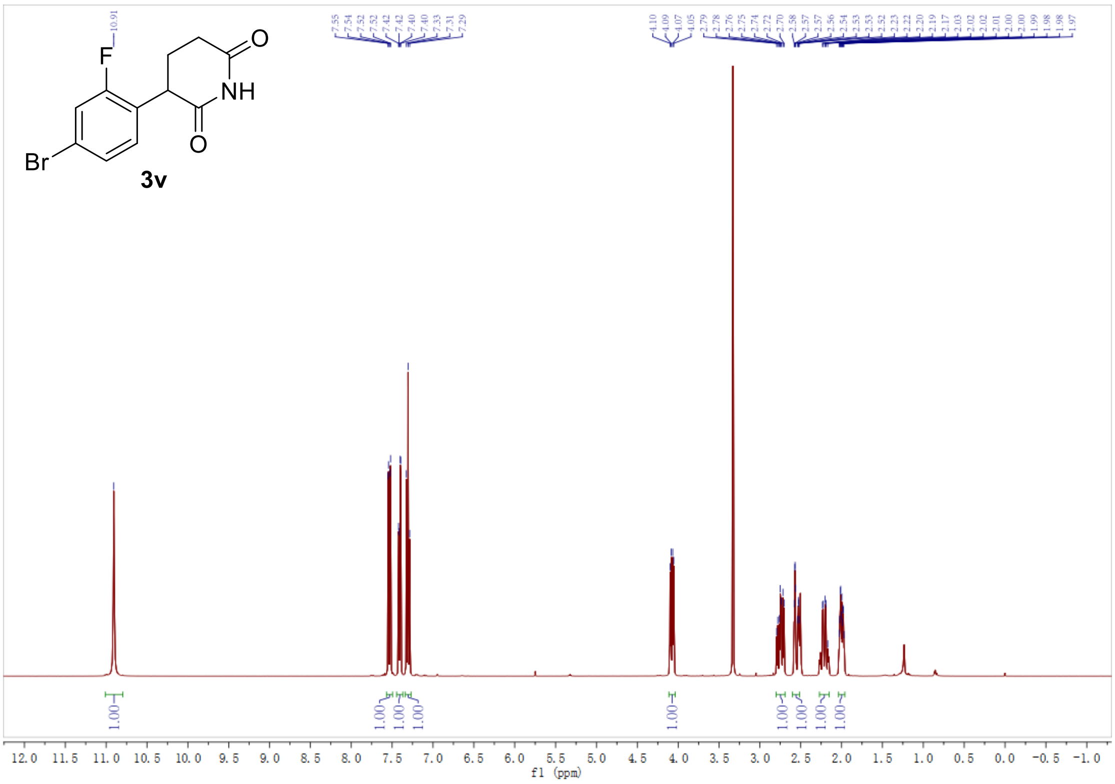

^{13}C NMR spectrum of 3-(4-bromo-2-fluorophenyl)piperidine-2,6-dione (3v). (DMSO-d_6, 100 MHz)

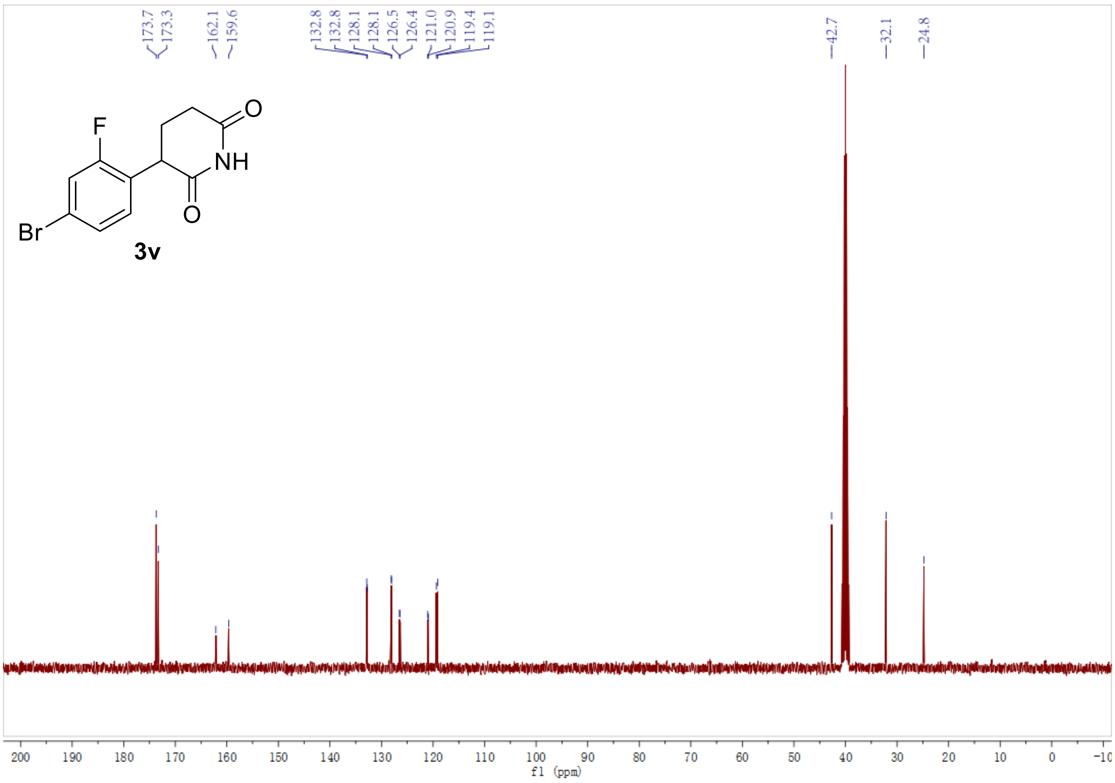

^{19}F NMR spectrum of 3-(4-bromo-2-fluorophenyl)piperidine-2,6-dione (3v). (DMSO-d_6, 376 MHz)

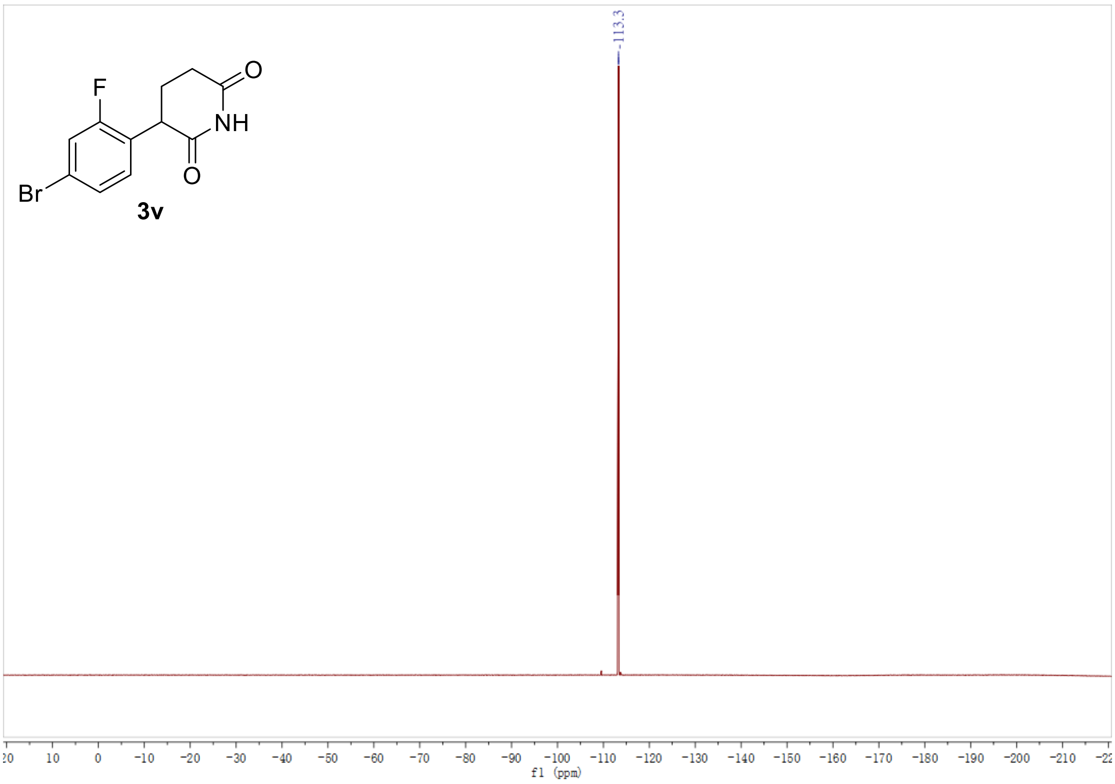

^{1}H NMR spectrum of 3-(4-bromo-3-fluorophenyl)piperidine-2,6-dione (3w). (DMSO-d_6, 400 MHz)

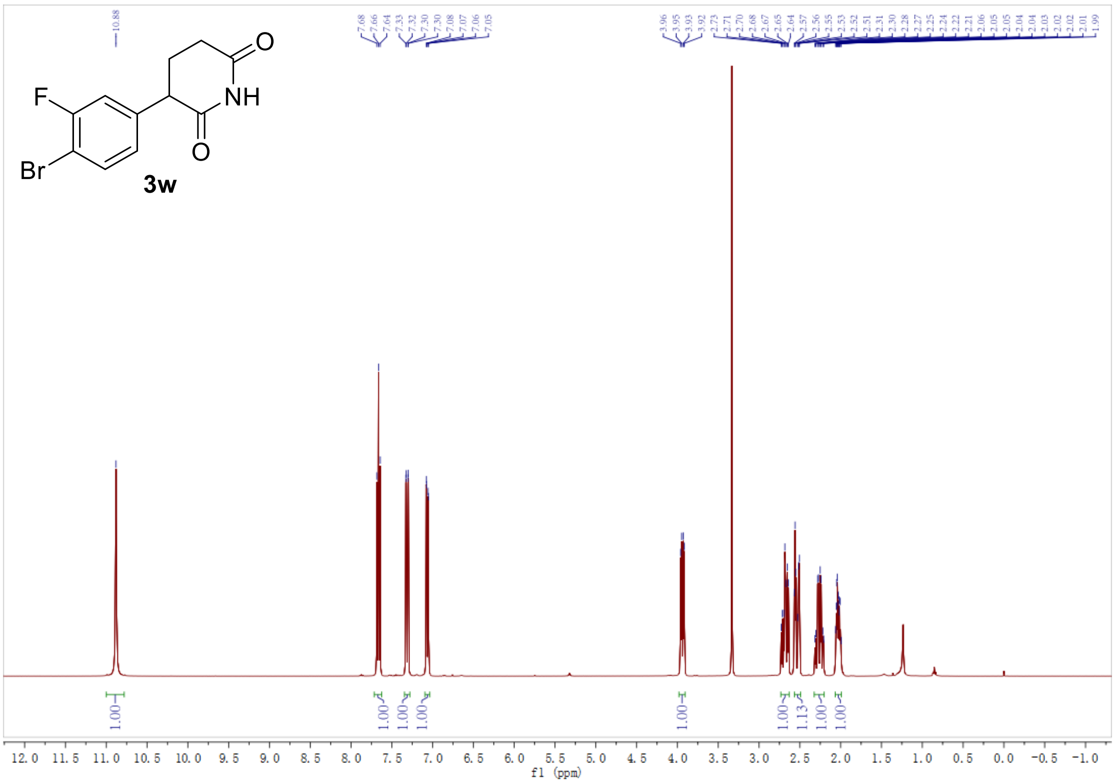

^{13}C NMR spectrum of 3-(4-bromo-3-fluorophenyl)piperidine-2,6-dione (3w). (DMSO-d_6, 100 MHz)

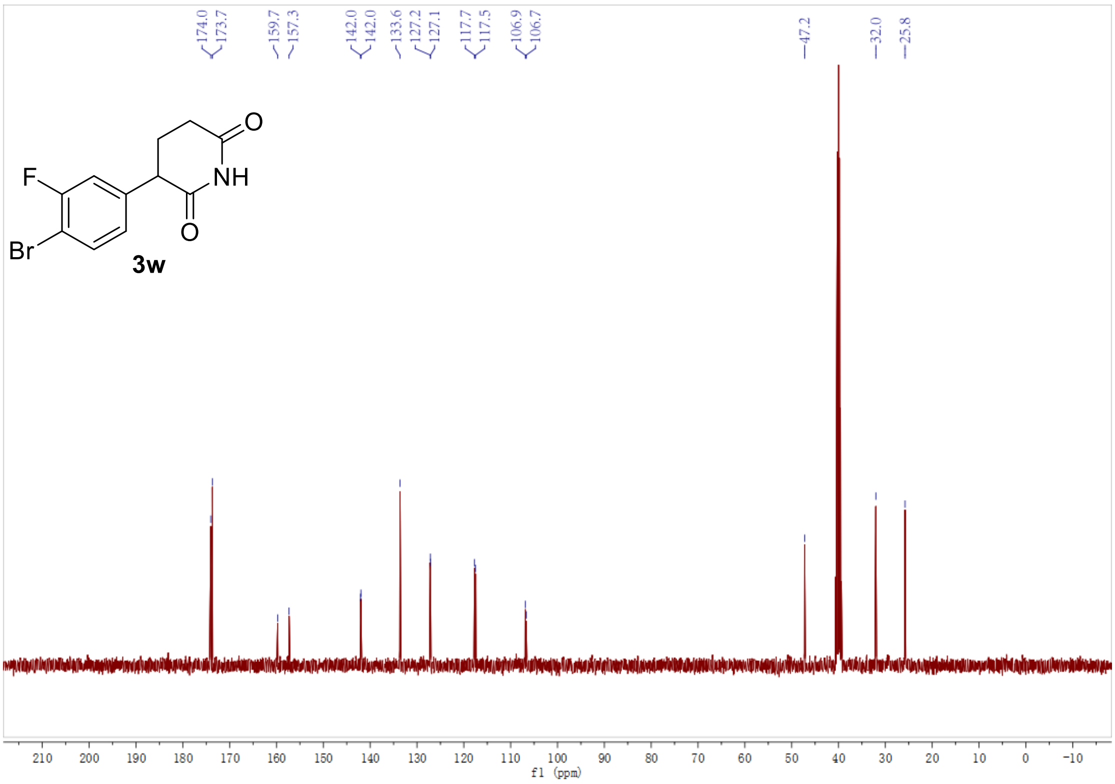

^{19}F NMR spectrum of 3-(4-bromo-3-fluorophenyl)piperidine-2,6-dione (3w). (DMSO-d_6, 377 MHz)

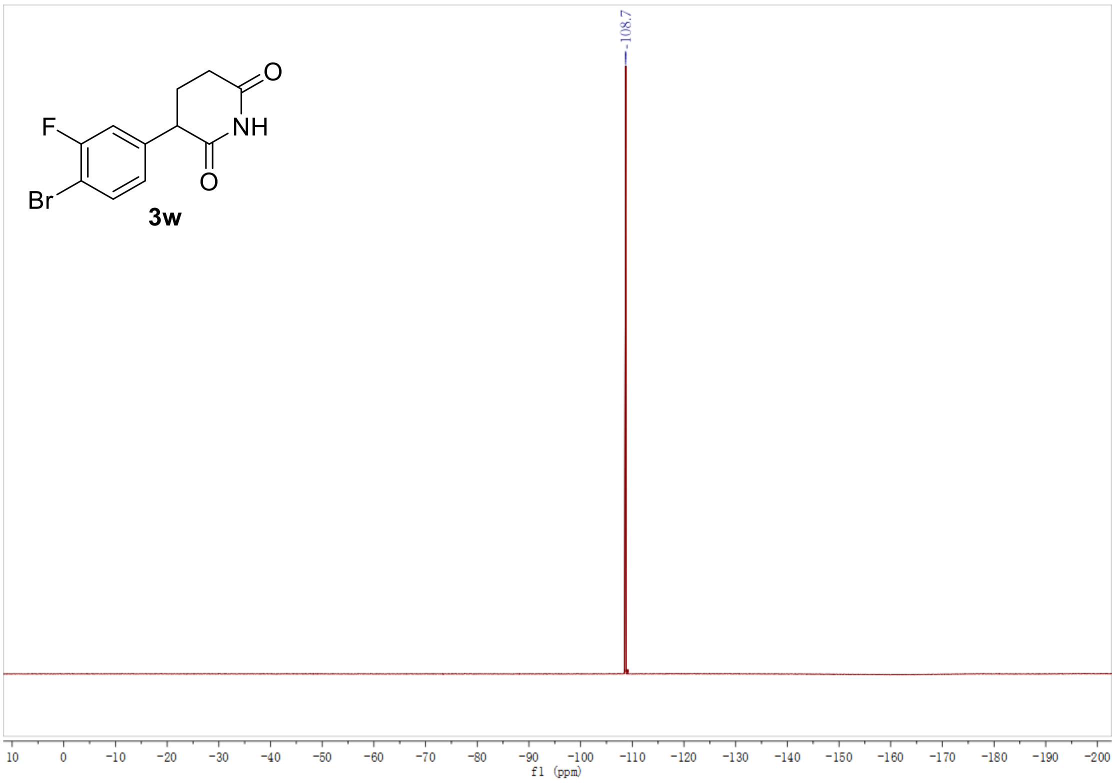

^{1}H NMR spectrum of 3-(4-isobutylphenyl)-3-methylpiperidine-2,6-dione (3z). (DMSO-d_6, 800 MHz)

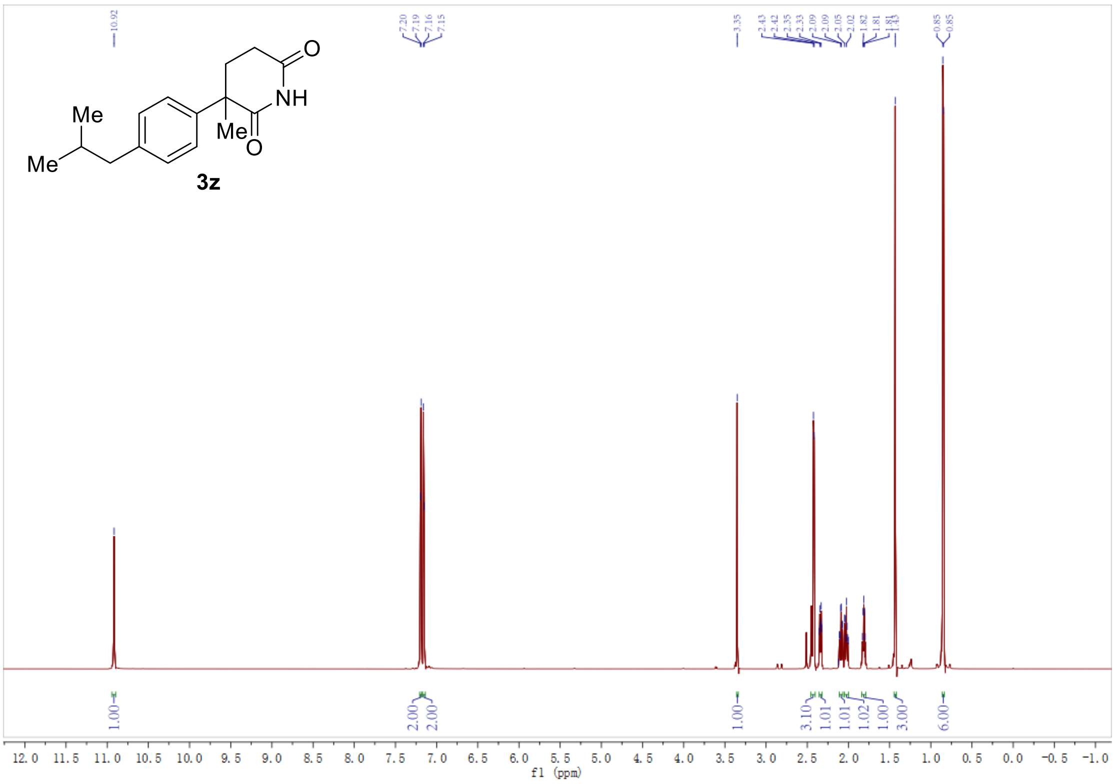

^{13}C NMR spectrum of 3-(4-isobutylphenyl)-3-methylpiperidine-2,6-dione (3z). (DMSO-d_6, 200 MHz)

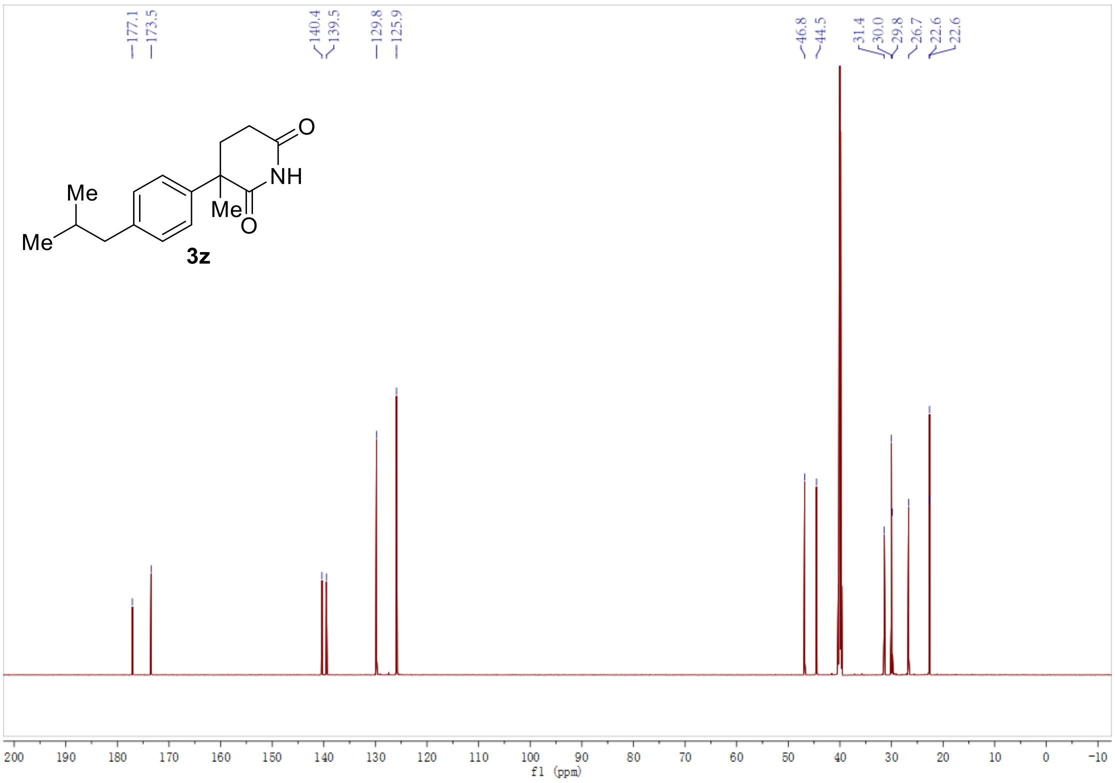

^{1}H NMR spectrum of 3,3-diphenylpiperidine-2,6-dione (3aa). (DMSO-d_6, 800 MHz)

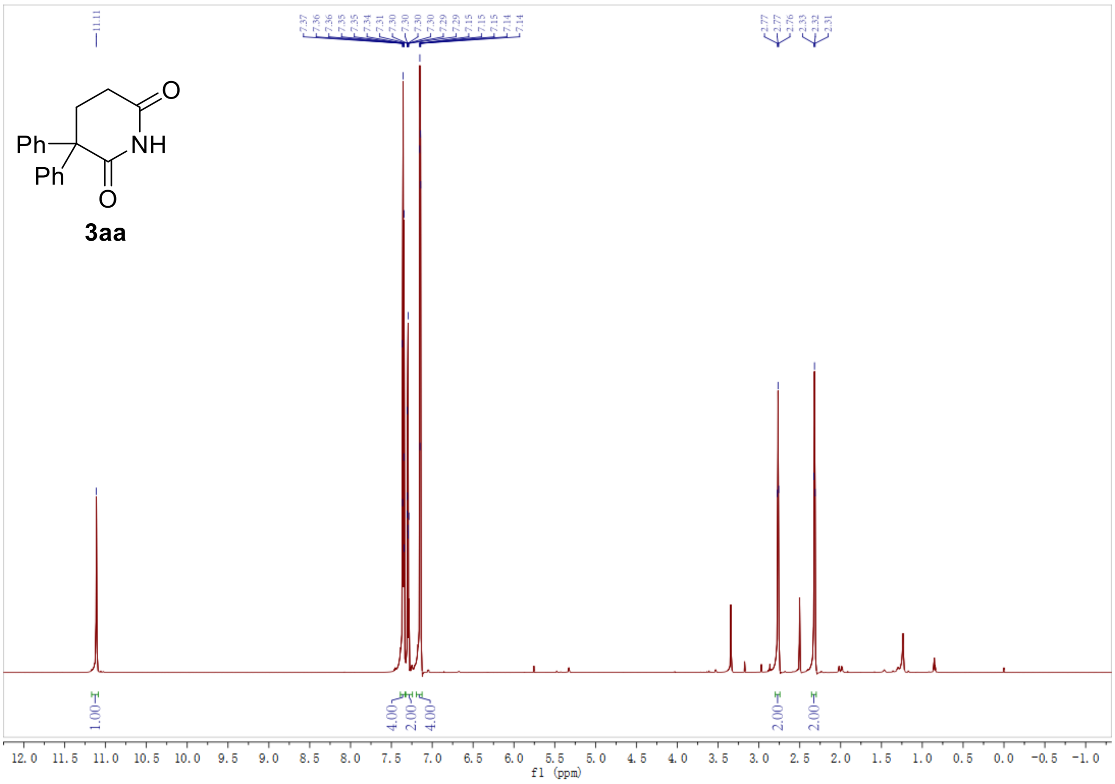

^{13}C NMR spectrum of 3,3-diphenylpiperidine-2,6-dione (3aa). (DMSO-d_6, 200 MHz)

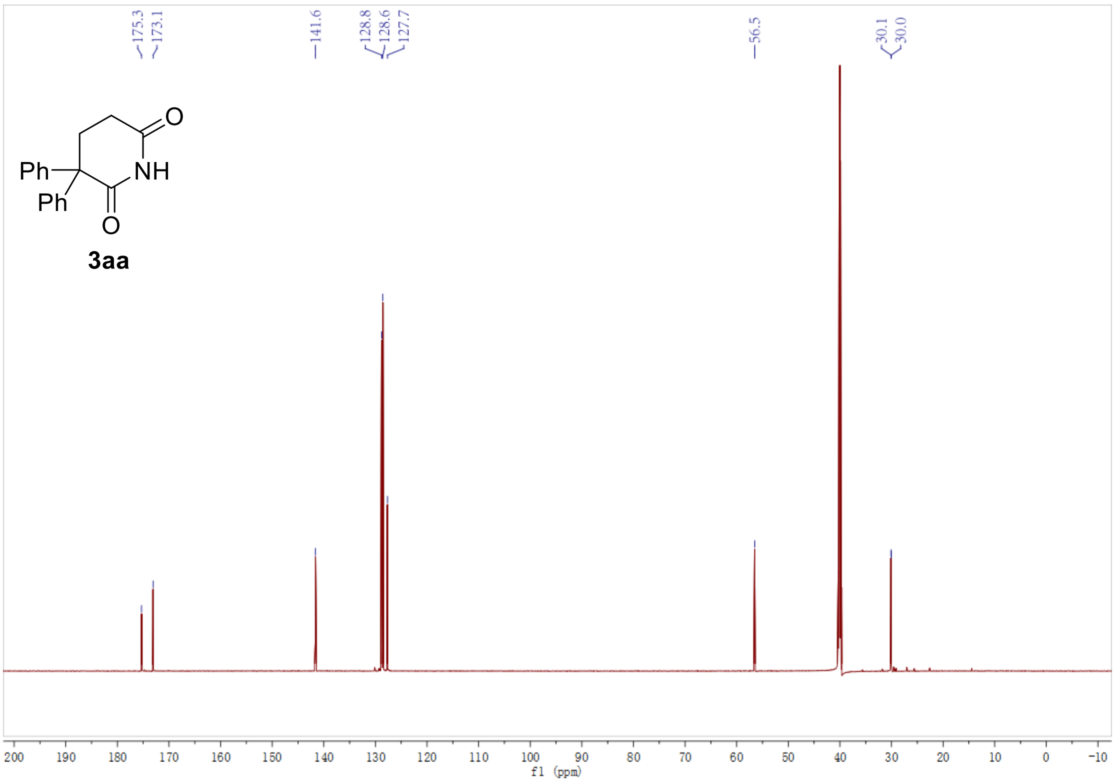

^{1}H NMR spectrum of 3-methyl-3-phenylpiperidine-2,6-dione (3ab). (DMSO-d_6, 800 MHz)

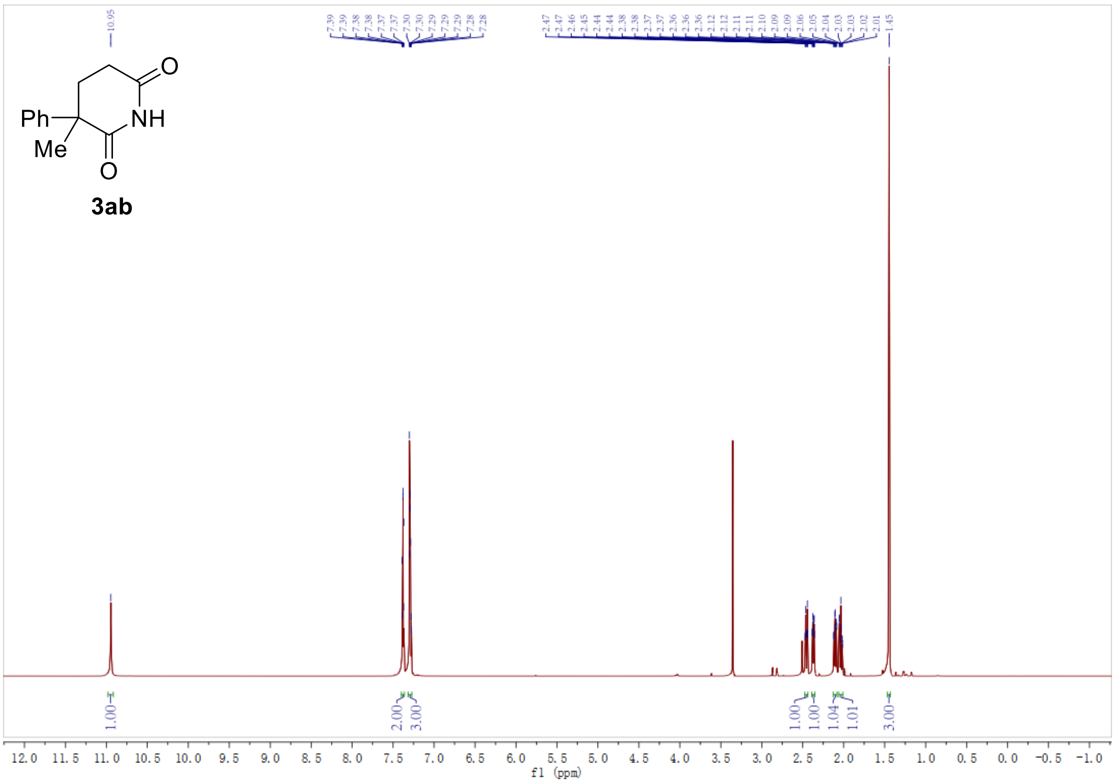

^{13}C NMR spectrum of 3-methyl-3-phenylpiperidine-2,6-dione(3ab). (DMSO-d_6, 200 MHz)

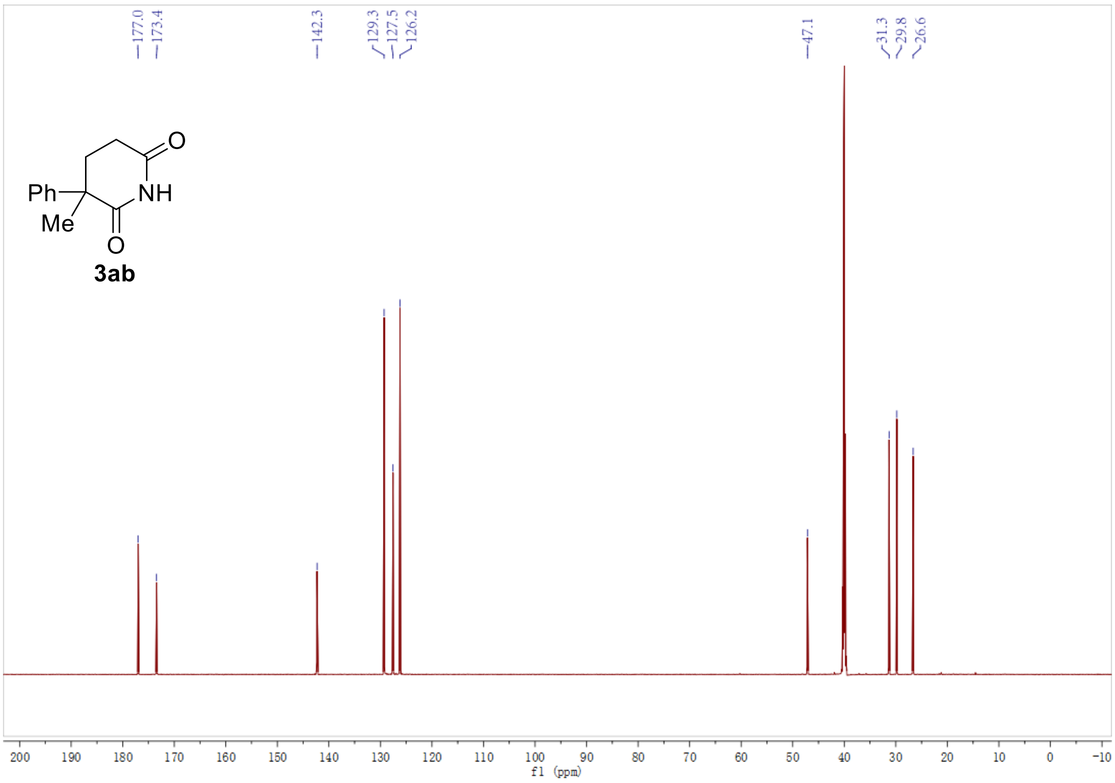

^{1}H NMR spectrum of 3-ethyl-3-phenylpiperidine-2,6-dione (3ac). (DMSO-d_6, 800 MHz)

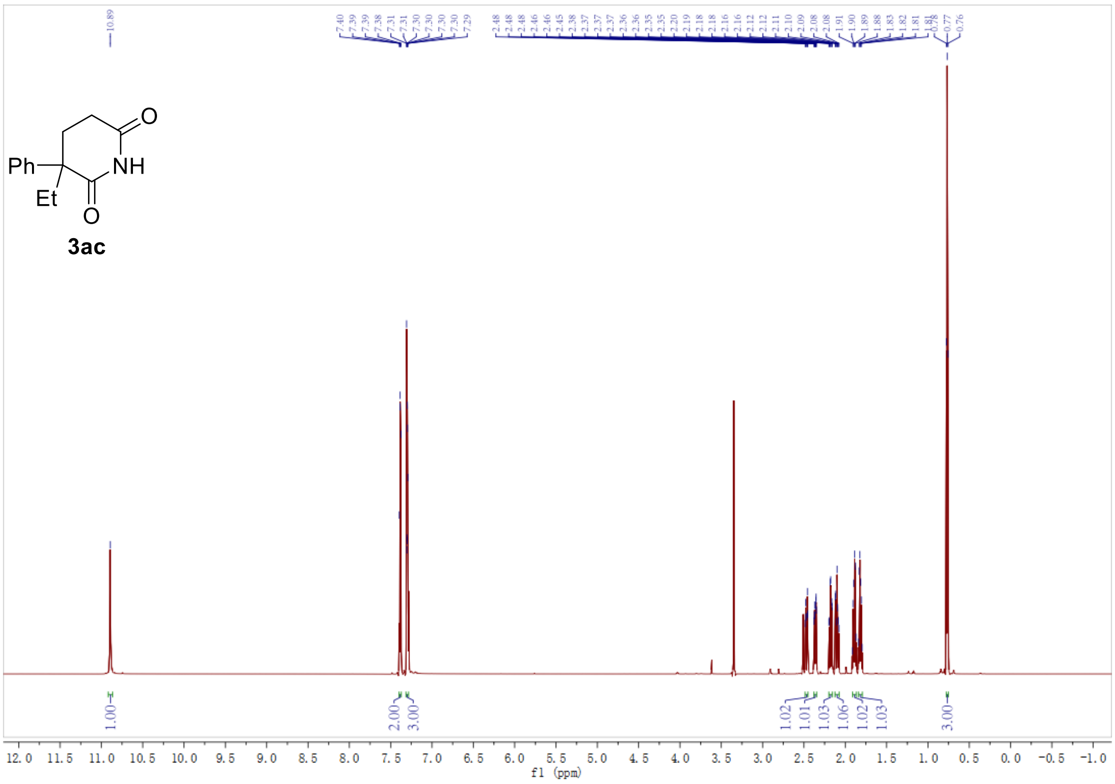

^{13}C NMR spectrum of 3-ethyl-3-phenylpiperidine-2,6-dione (3ac). (DMSO-d_6, 200 MHz)

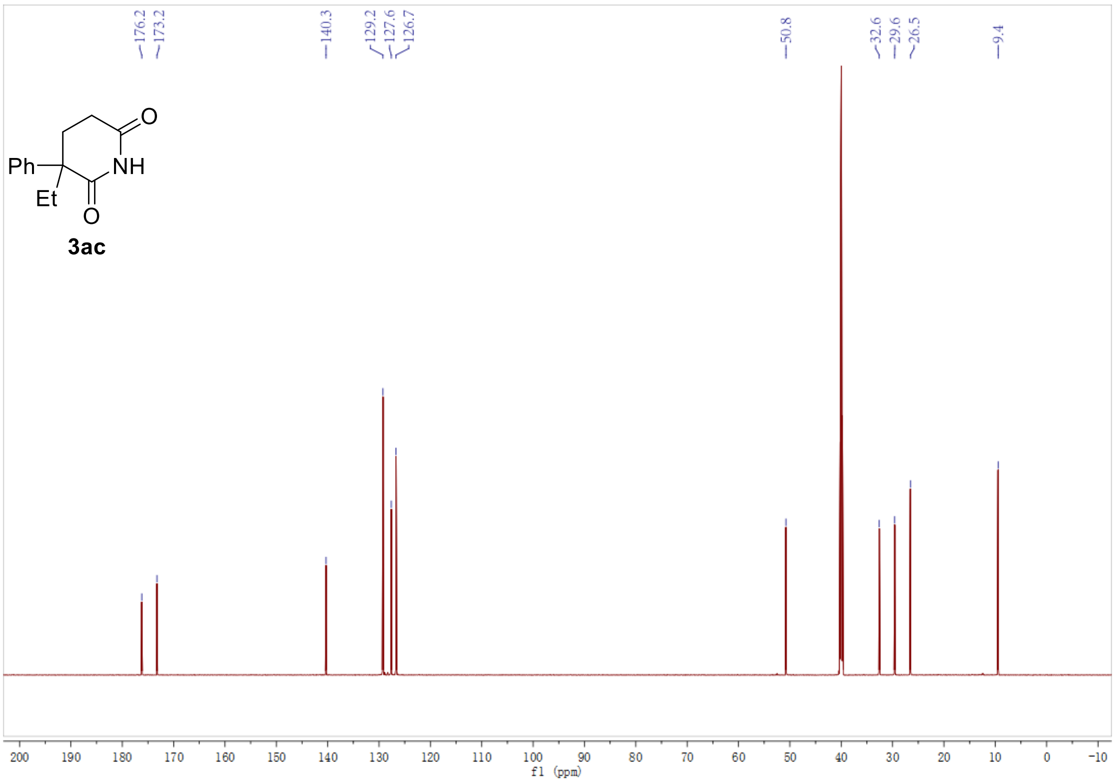

^{1}H NMR spectrum of 3-acetylpiperidine-2,6-dione (3ad). (DMSO-d_6, 800 MHz)

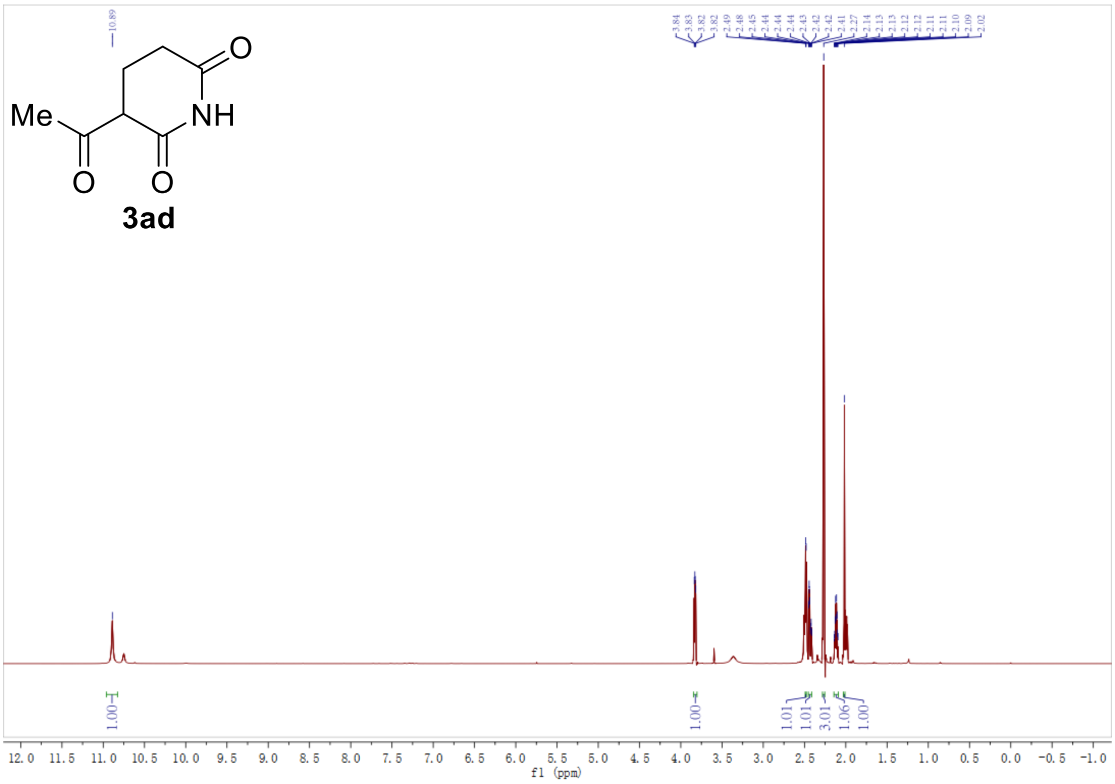

^{13}C NMR spectrum of 3-acetylpiperidine-2,6-dione (3ad). (DMSO-d_6, 200 MHz)

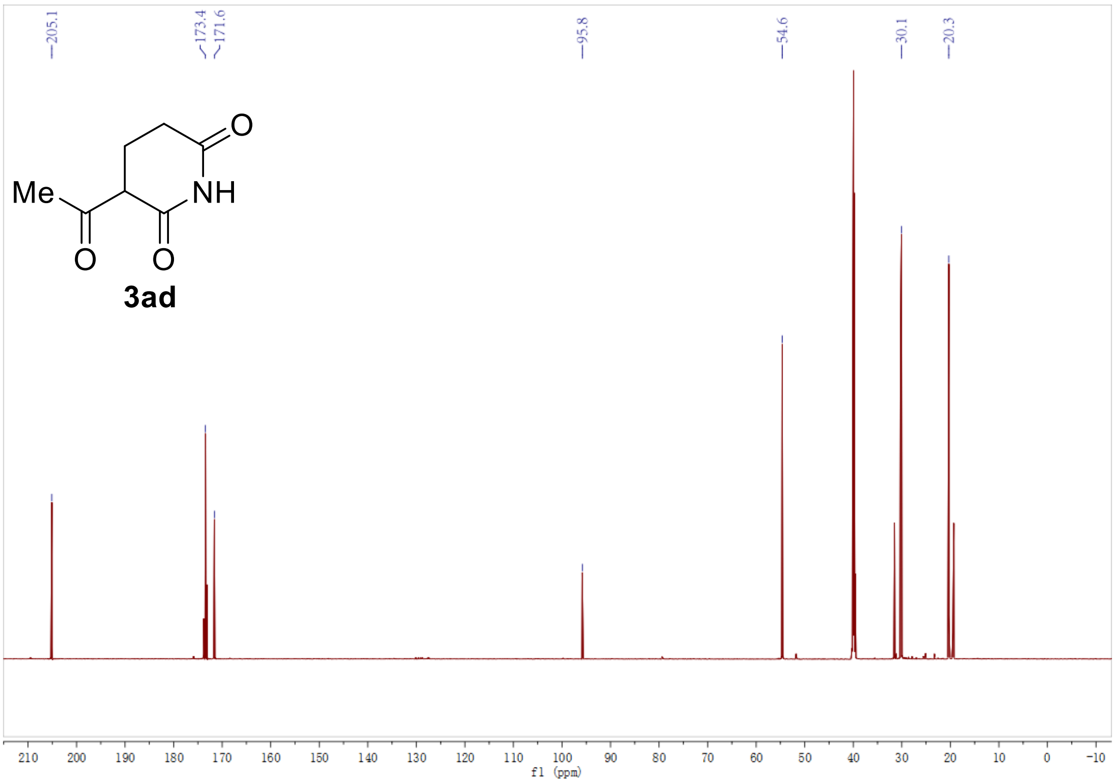

^{1}H NMR spectrum of ethyl 2,6-dioxopiperidine-3-carboxylate (3ae). ($CDCl_3$, 800 MHz)

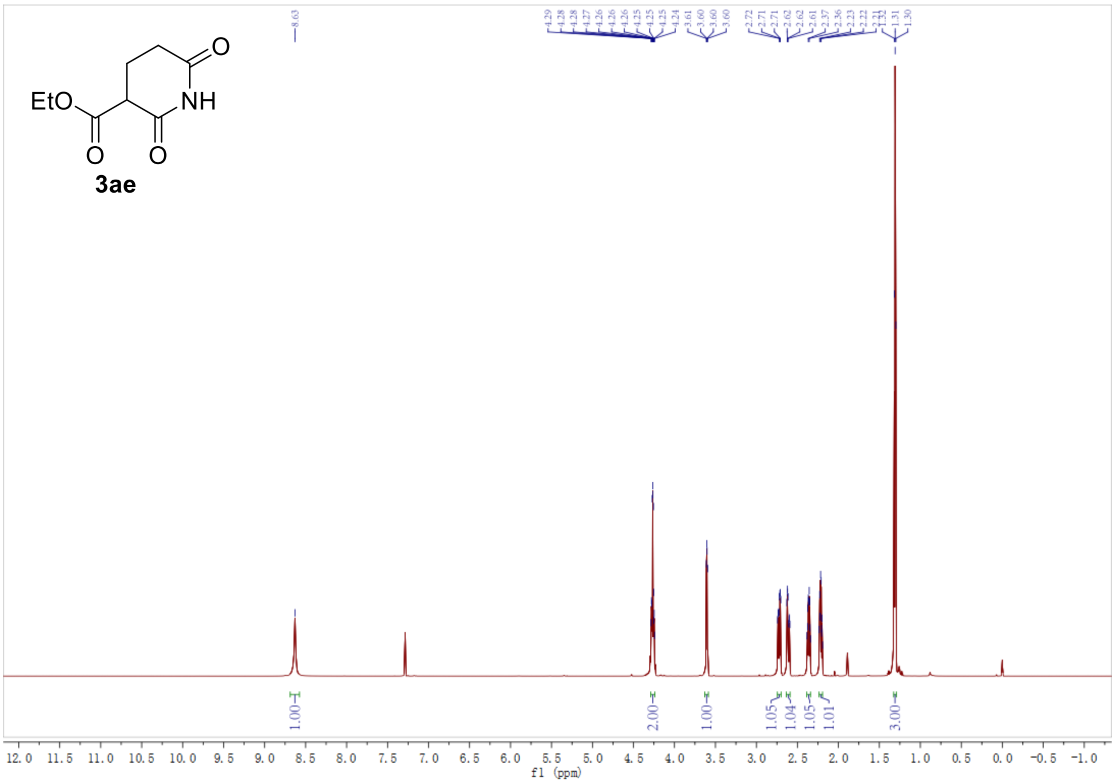

^{13}C NMR spectrum of ethyl 2,6-dioxopiperidine-3-carboxylate (3ae). ($CDCl_3$, 200 MHz)

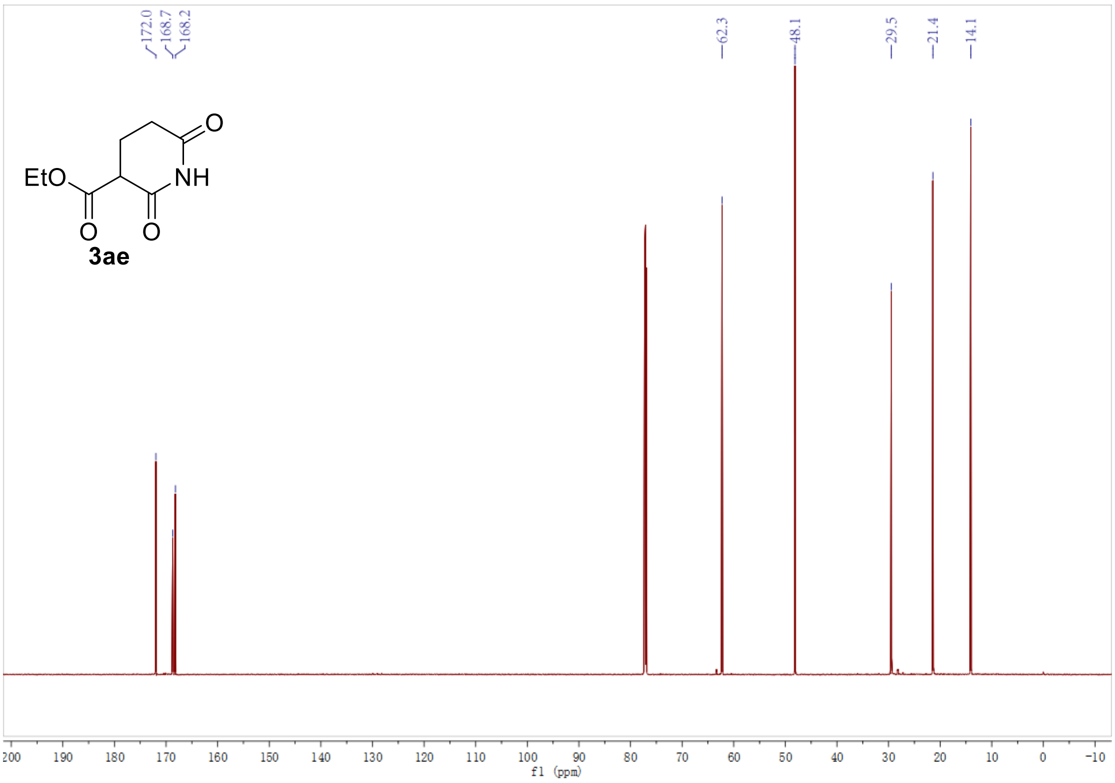

^{1}H NMR spectrum of methyl 4-(4-fluorophenyl)-2,6-dioxopiperidine-3-carboxylate (3af). (DMSO-d_6, 400 MHz)

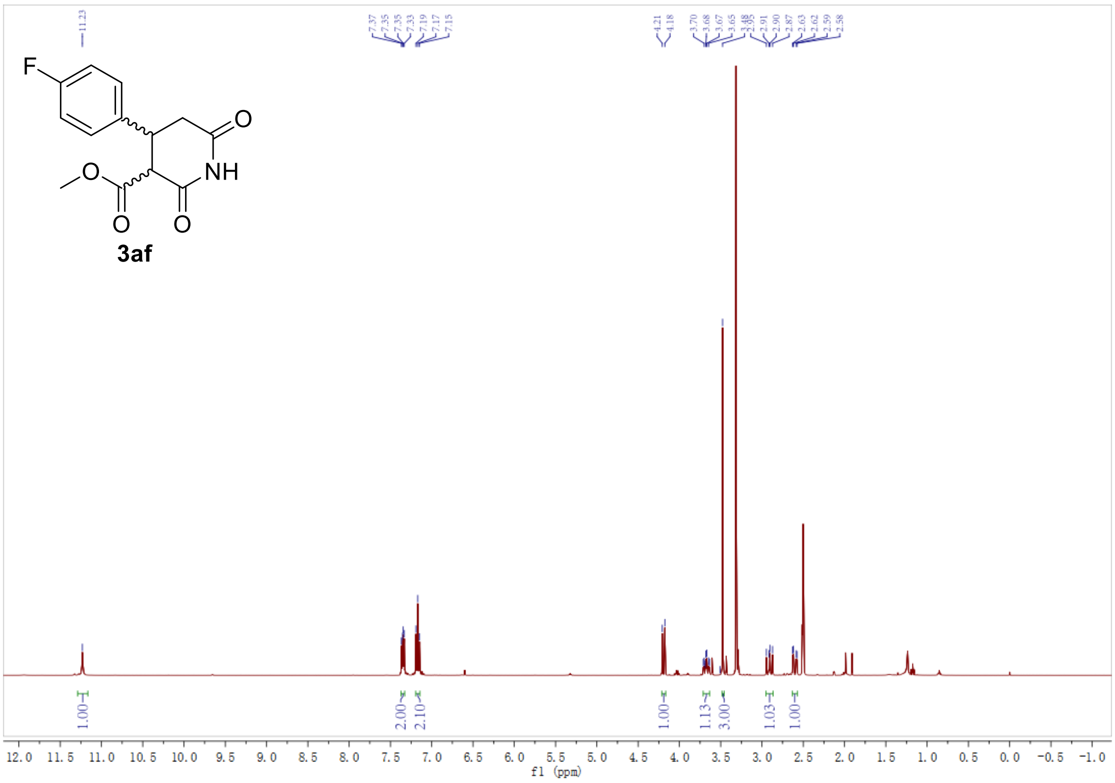

^{13}C NMR spectrum of methyl 4-(4-fluorophenyl)-2,6-dioxopiperidine-3-carboxylate (3af).

(DMSO-d_6,100 MHz)

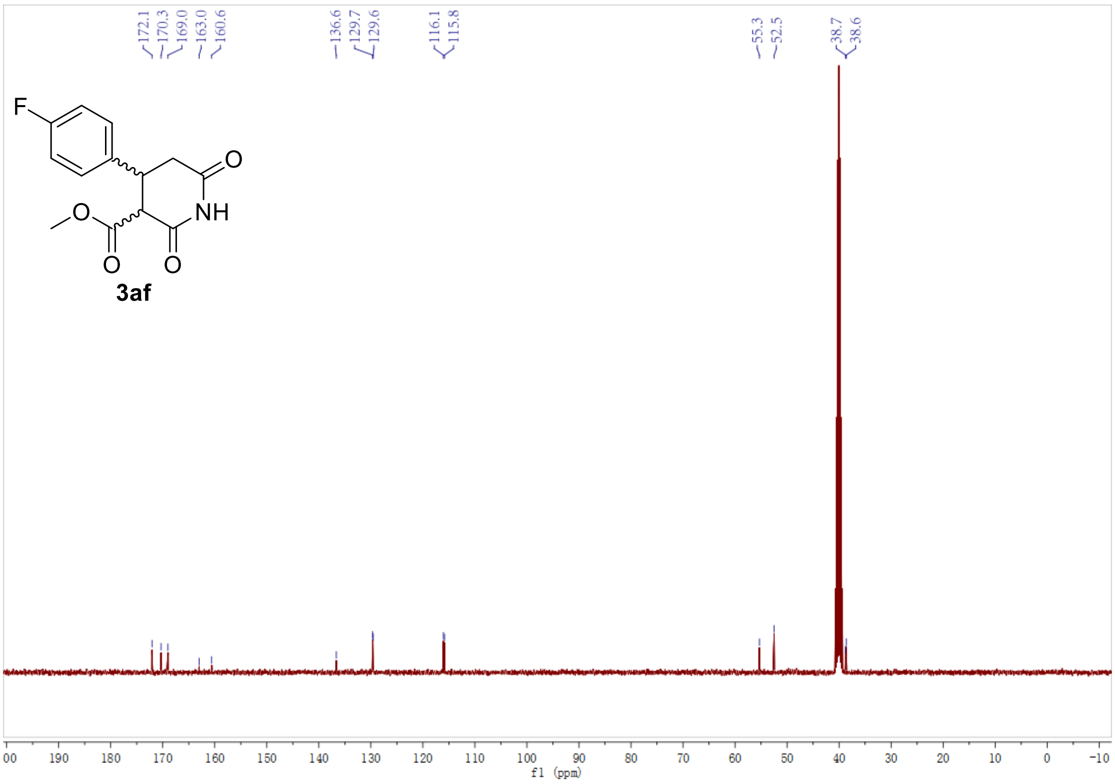

^{1}H NMR spectrum of 3-(4-bromophenyl)-4-phenylpiperidine-2,6-dione (3ag). (DMSO-d_6, 800 MHz)

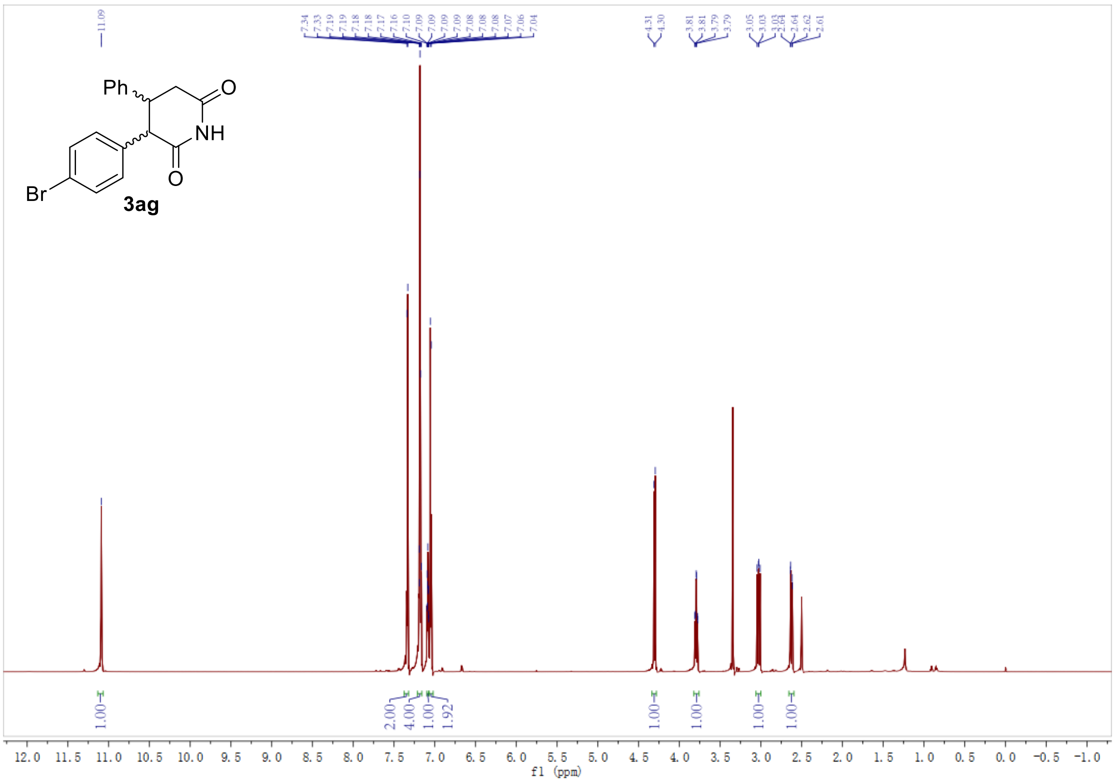

^{13}C NMR spectrum of 3-(4-bromophenyl)-4-phenylpiperidine-2,6-dione (3ag). (DMSO-d_6, 200 MHz)

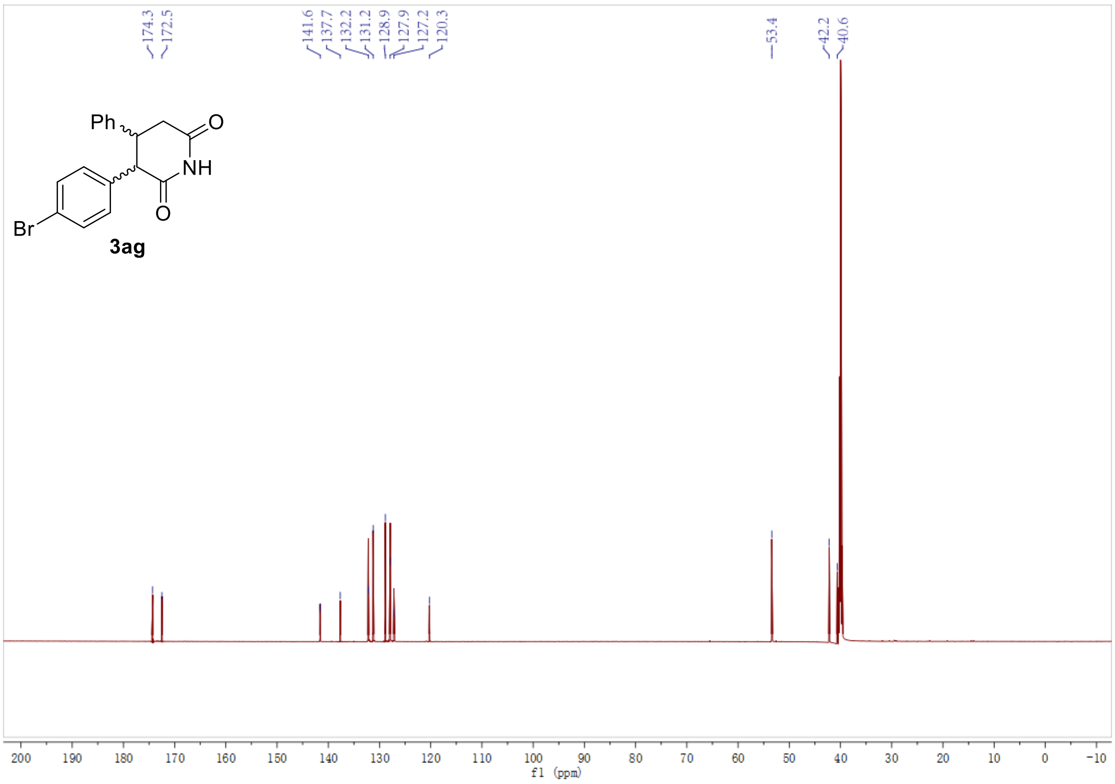

^{1}H NMR spectrum of 3-(4-chlorophenyl)-4-phenylpiperidine-2,6-dione (3ah). (DMSO-d_6, 800 MHz)

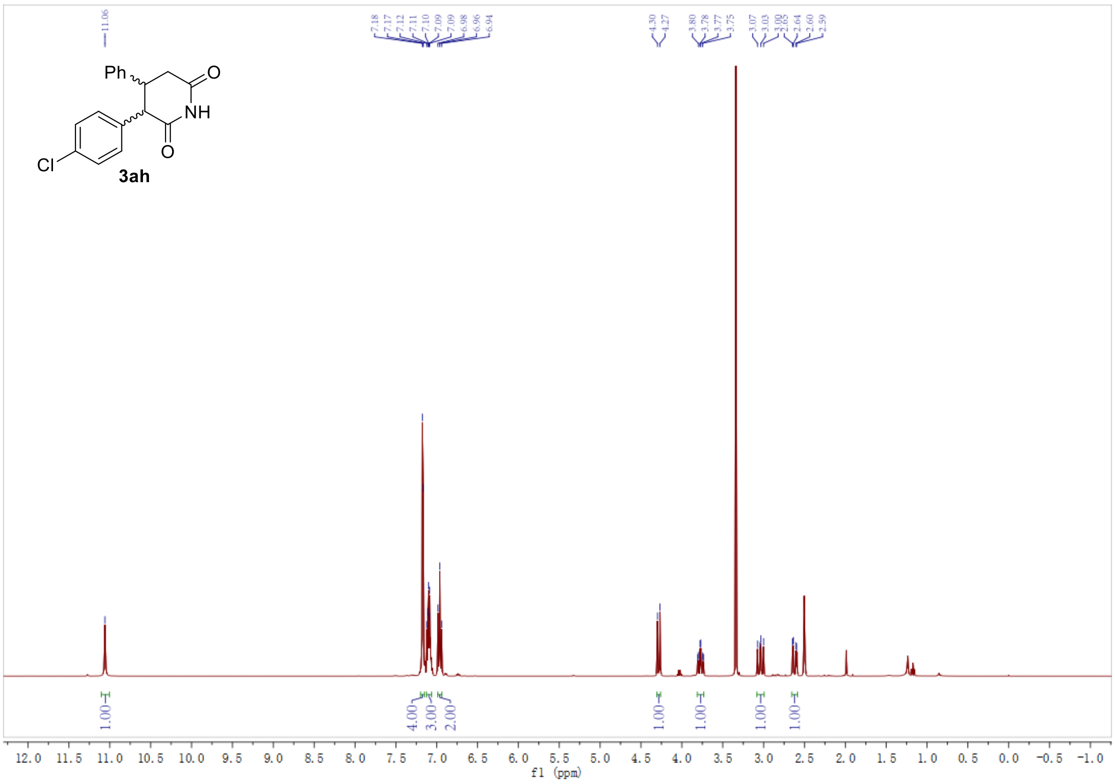

^{13}C NMR spectrum of 3-(4-chlorophenyl)-4-phenylpiperidine-2,6-dione (3ah). (DMSO-d_6, 200 MHz)

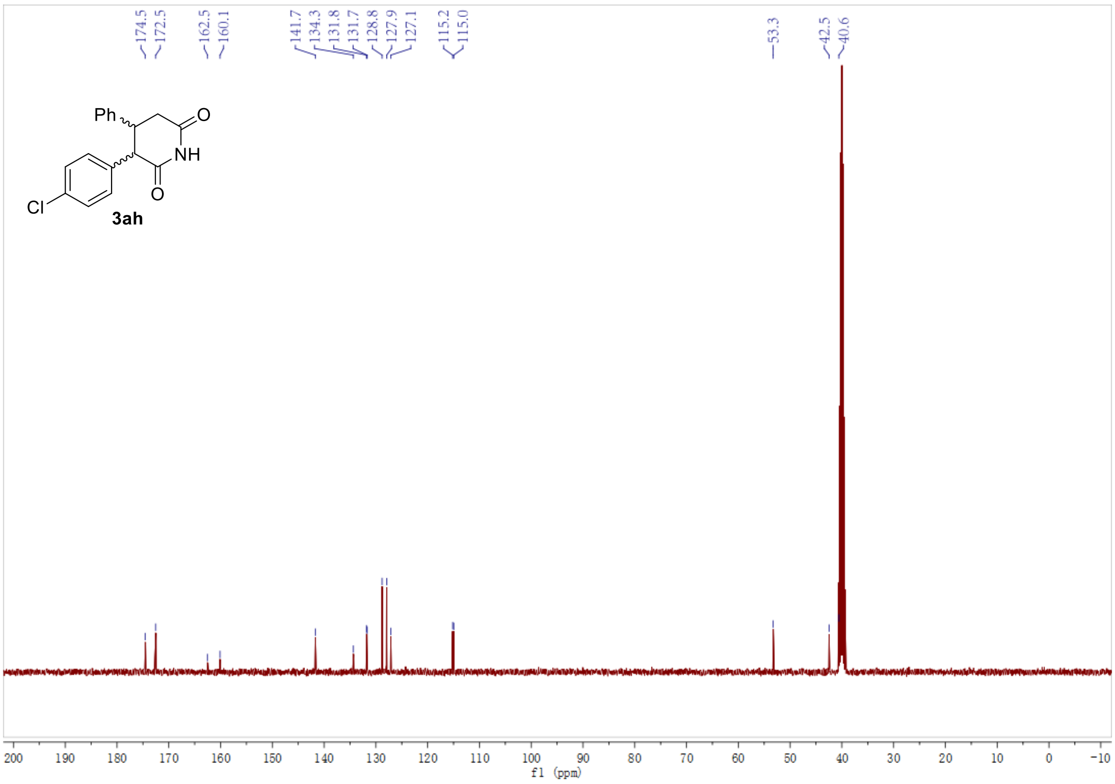

^{1}H NMR spectrum of 3-(4-fluorophenyl)-4-phenylpiperidine-2,6-dione (3ai). (DMSO-d_6, 800 MHz)

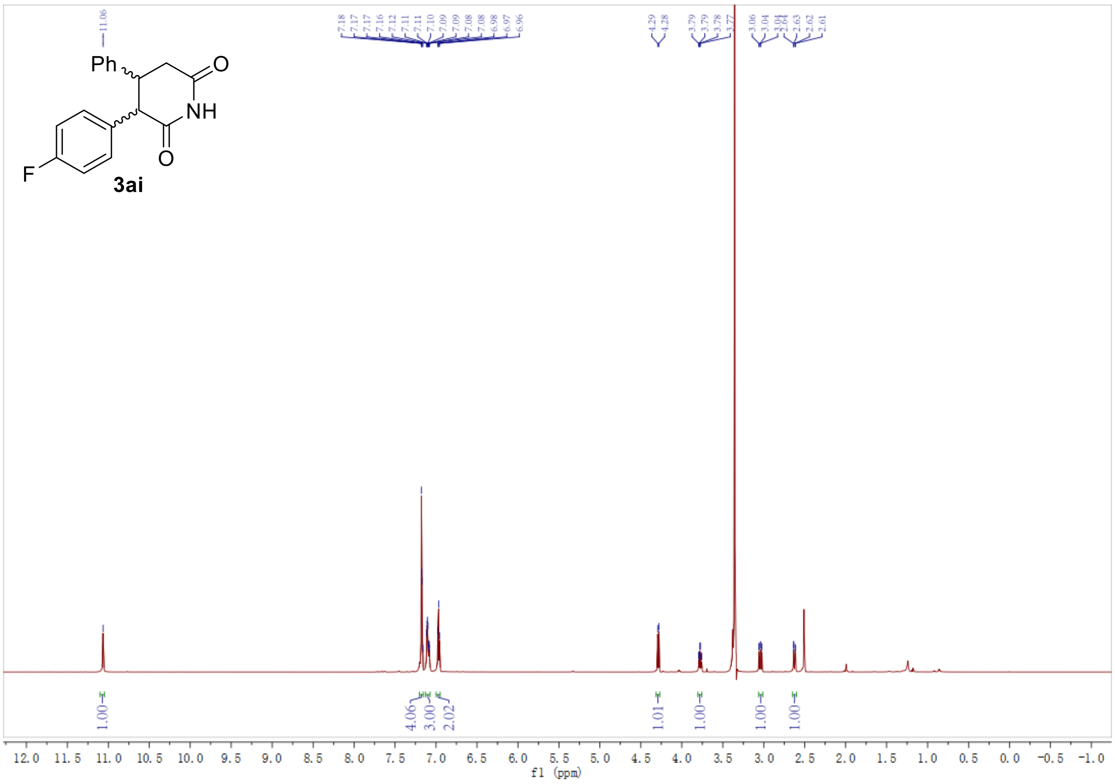

^{13}C NMR spectrum of 3-(4-chlorophenyl)-4-phenylpiperidine-2,6-dione (3ai). (DMSO-d_6, 200 MHz)

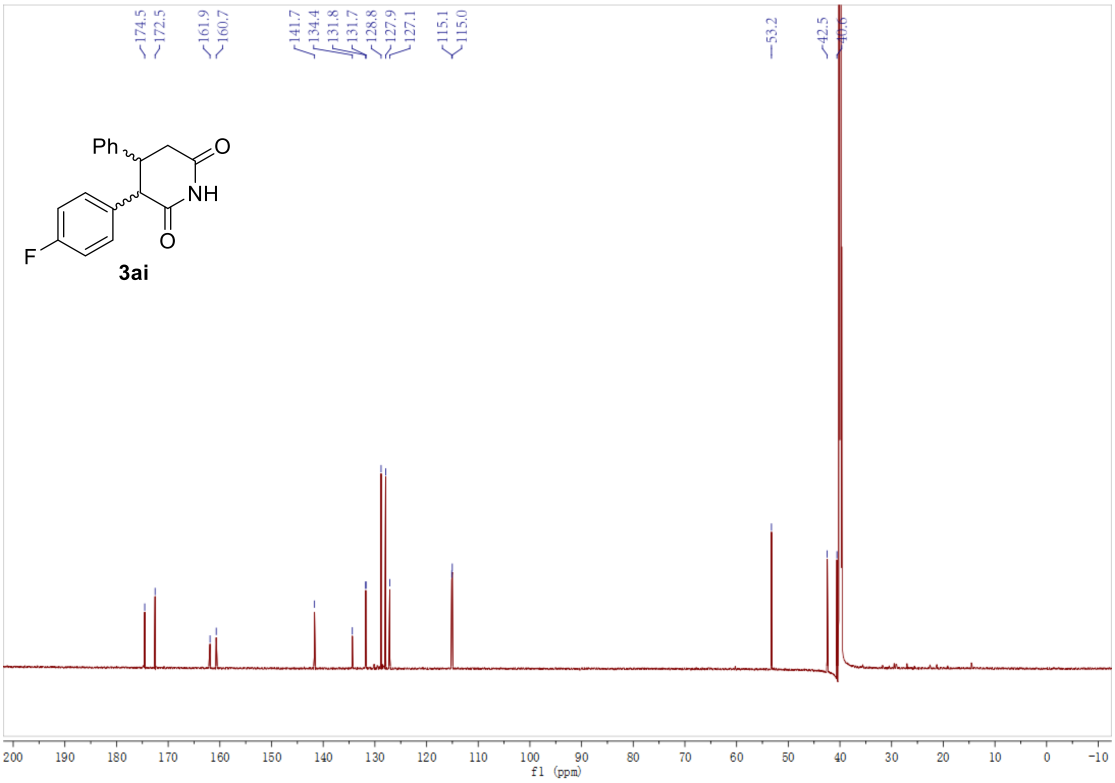

^{19}F NMR spectrum of 3-(4-chlorophenyl)-4-phenylpiperidine-2,6-dione (3ai). (DMSO-d_6, 377 MHz)

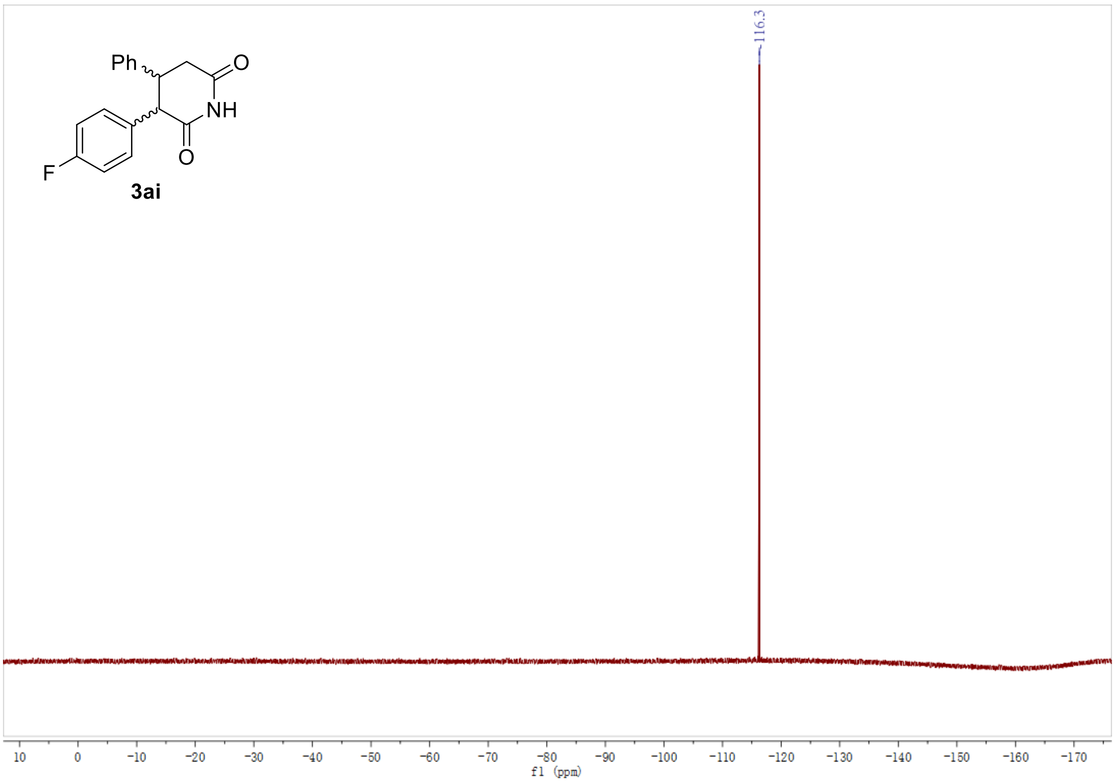

^{1}H NMR spectrum of 3-(4-methoxyphenyl)-4-phenylpiperidine-2,6-dione (3aj). (DMSO-d_6, 800 MHz)

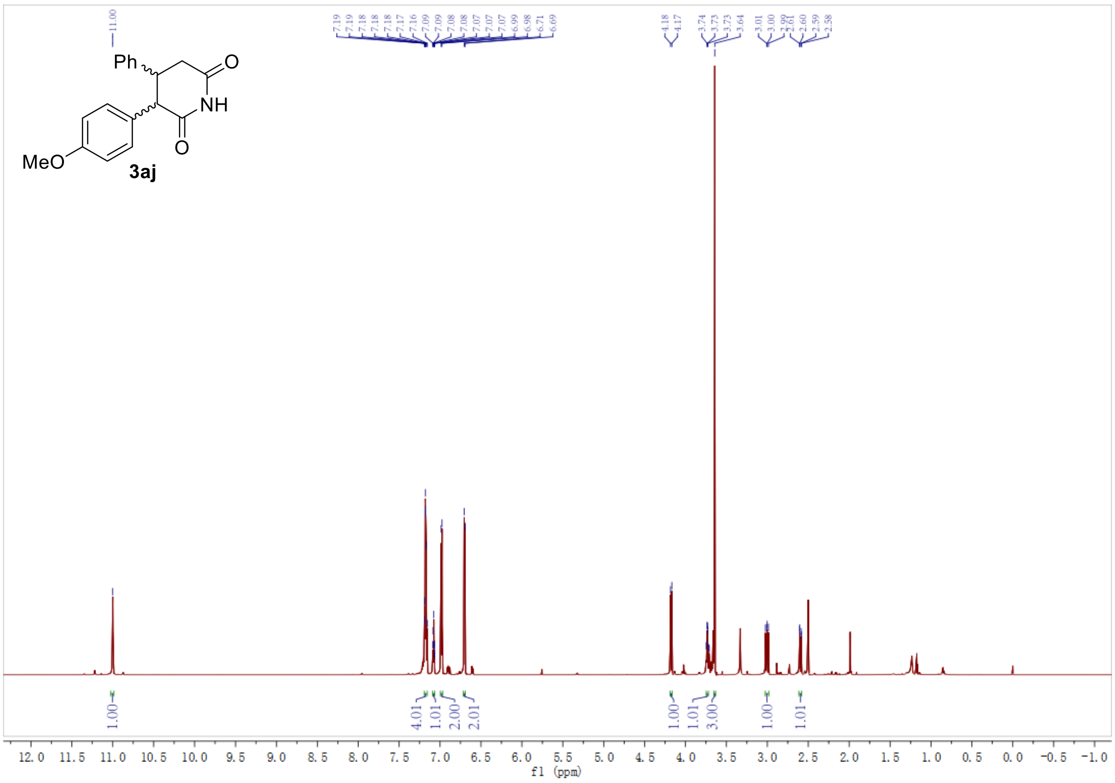

^{13}C NMR spectrum of 3-(4-methoxyphenyl)-4-phenylpiperidine-2,6-dione (3aj). (DMSO-d_6, 200 MHz)

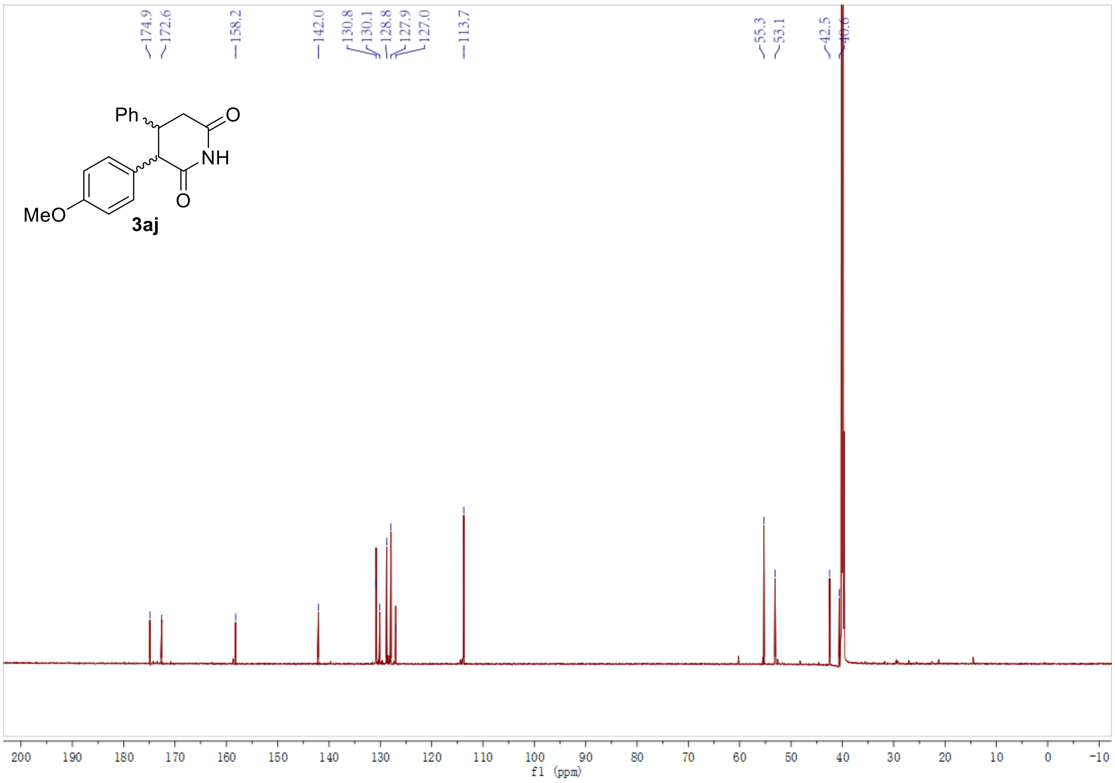

^{1}H NMR spectrum of 3-(4-bromophenyl)-1-methylpiperidine-2,6-dione (3ak). (DMSO-d_6, 800 MHz)

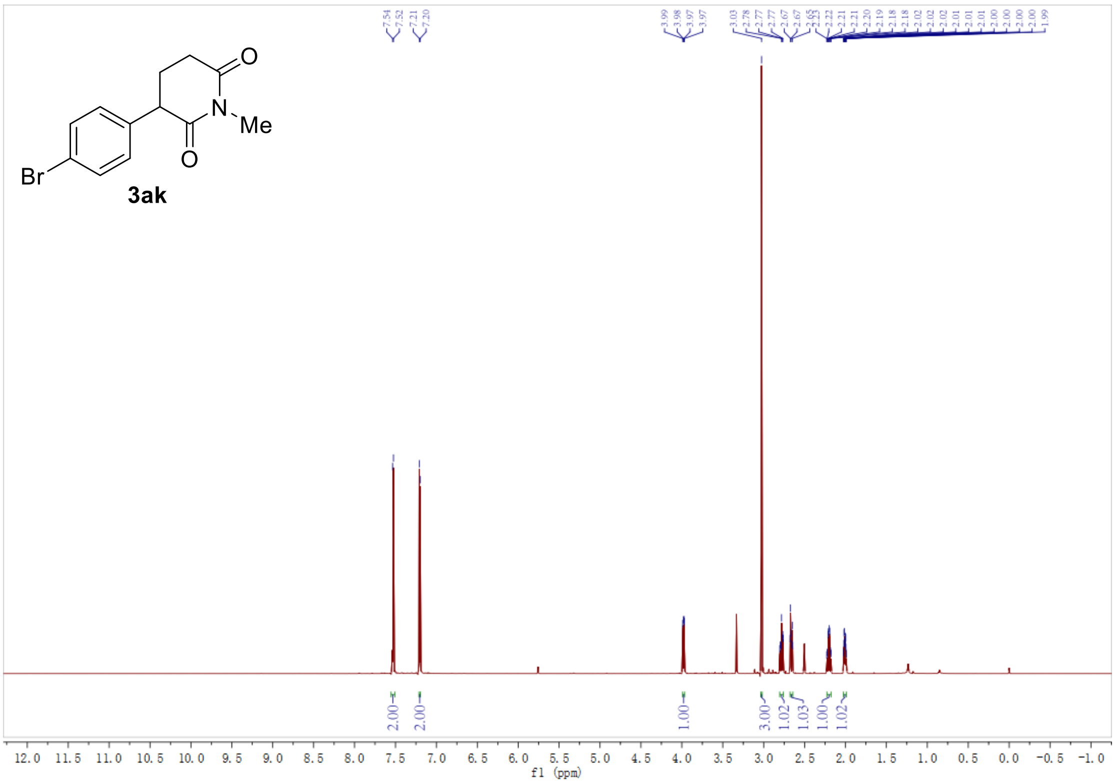

^{13}C NMR spectrum of 3-(4-bromophenyl)-1-methylpiperidine-2,6-dione (3ak). (DMSO-d_6, 200

MHz)

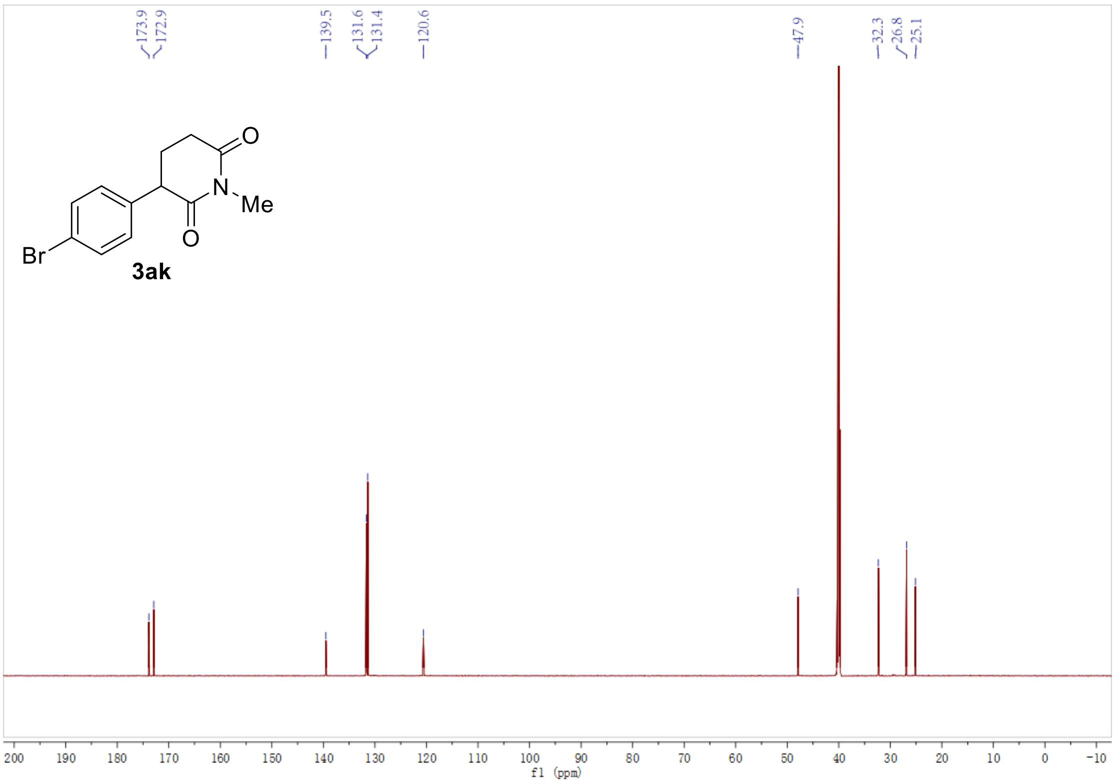

^{1}H NMR spectrum of 5-(4-bromophenyl)-6-methyl-3,4-dihydropyridin-2(1*H*)-one (5a). (DMSO-d_6, 800 MHz)

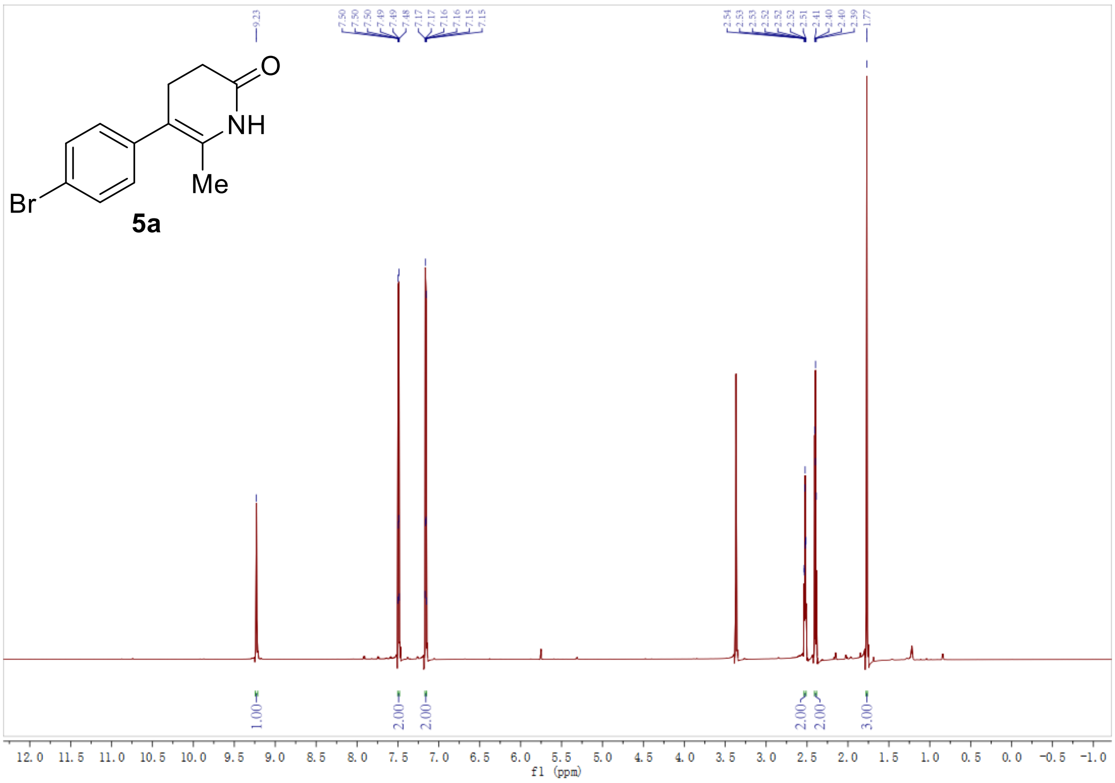

^{13}C NMR spectrum of 5-(4-bromophenyl)-6-methyl-3,4-dihydropyridin-2(1*H*)-one (5a).

(DMSO-d_6, 200 MHz)

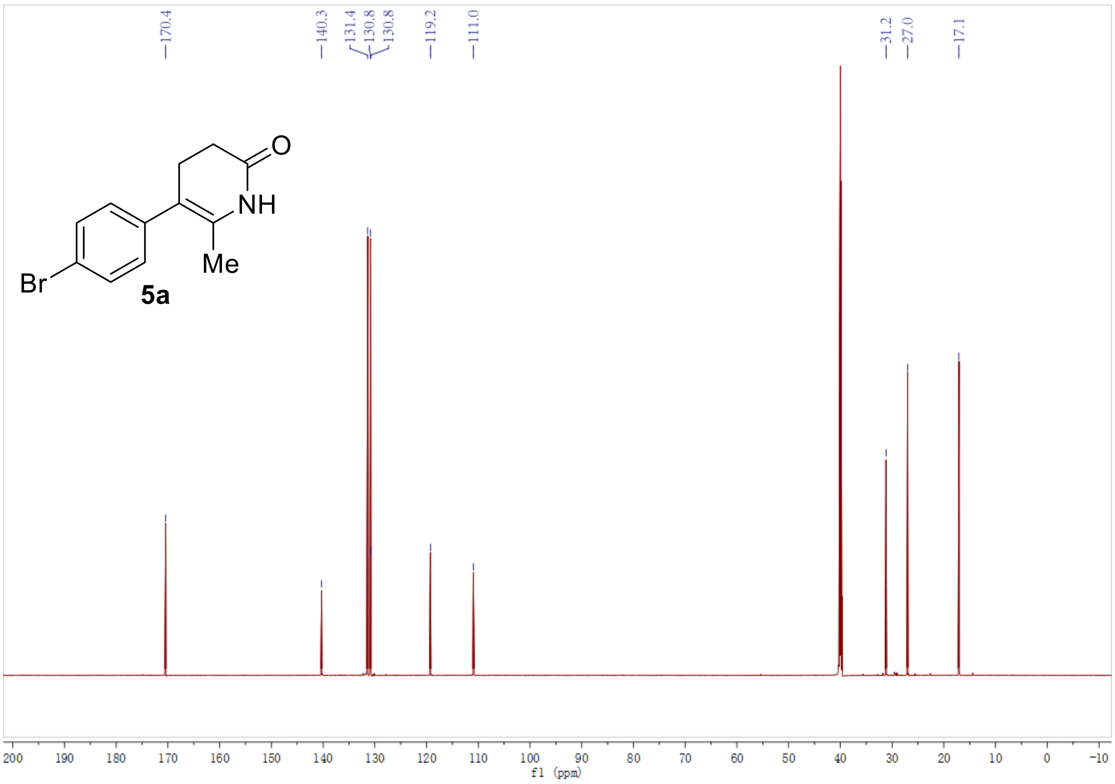

^{1}H NMR spectrum of 5-(4-chlorophenyl)-6-methyl-3,4-dihydropyridin-2(1*H*)-one (5b). (DMSO-d_6, 800 MHz)

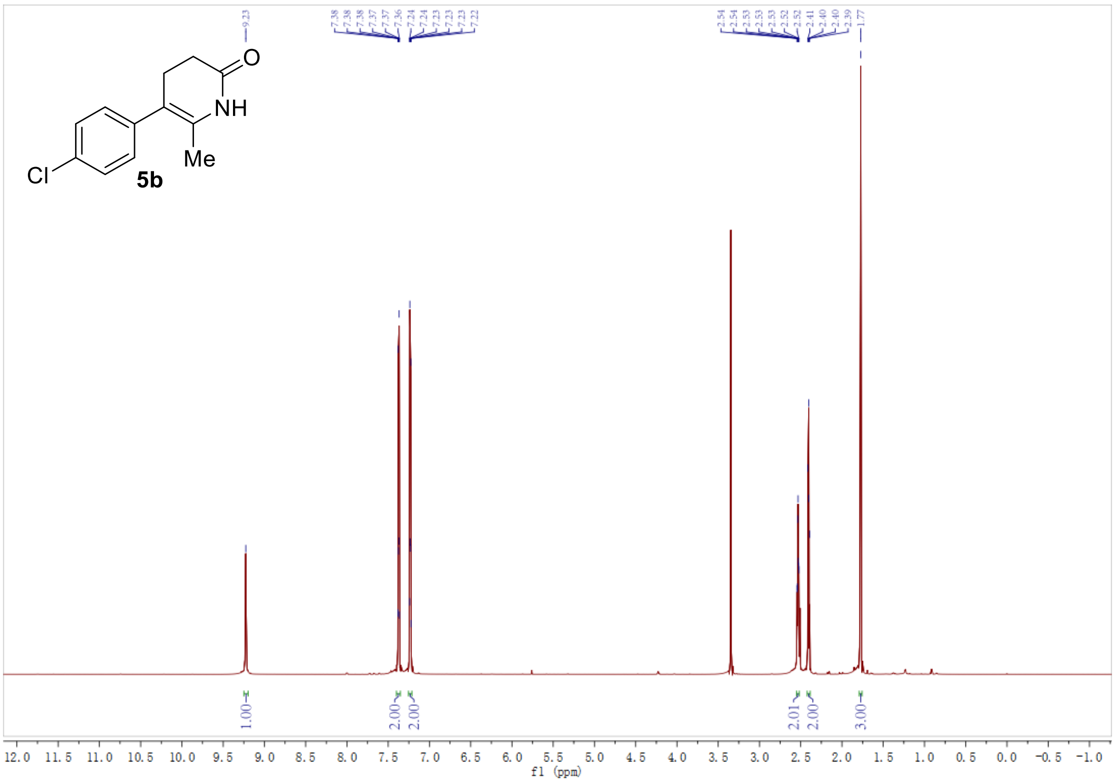

^{13}C NMR spectrum of 5-(4-chlorophenyl)-6-methyl-3,4-dihydropyridin-2(1*H*)-one (5b).

(DMSO-d_6, 200 MHz)

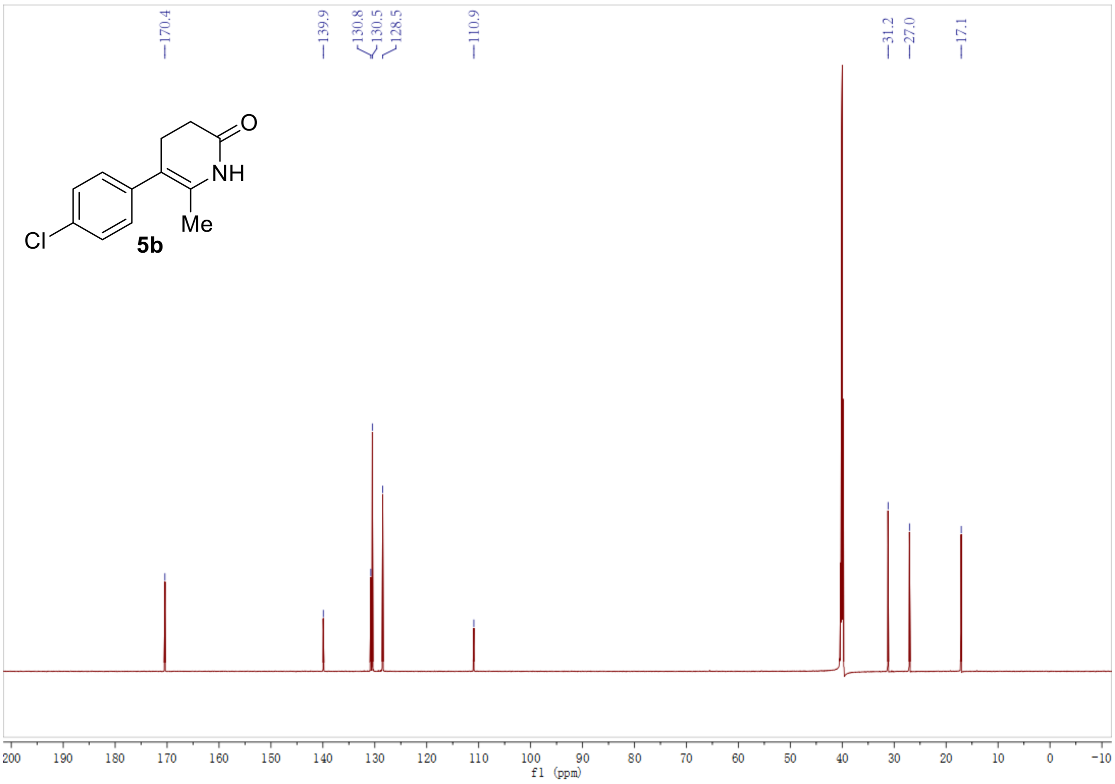

^{1}H NMR spectrum of 5-(4-fluorophenyl)-6-methyl-3,4-dihydropyridin-2(1*H*)-one (5c). (DMSO-d_6, 800 MHz)

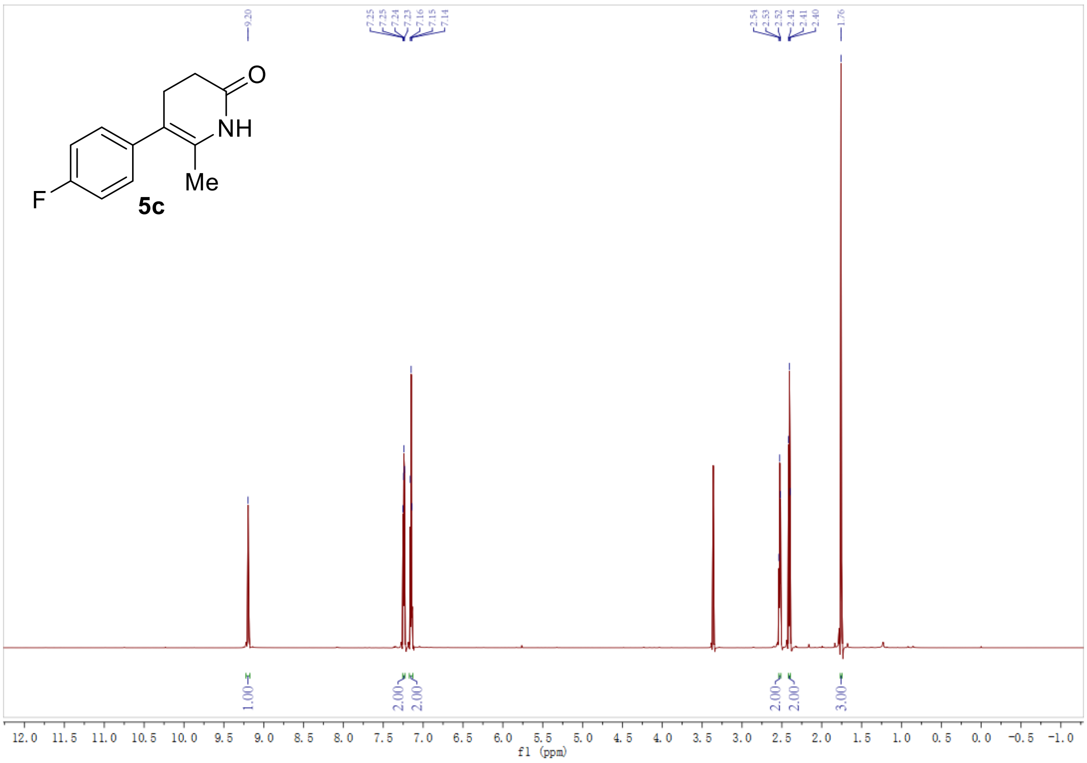

^{13}C NMR spectrum of 5-(4-fluorophenyl)-6-methyl-3,4-dihydropyridin-2(1*H*)-one (5c). (DMSO-d_6, 200 MHz)

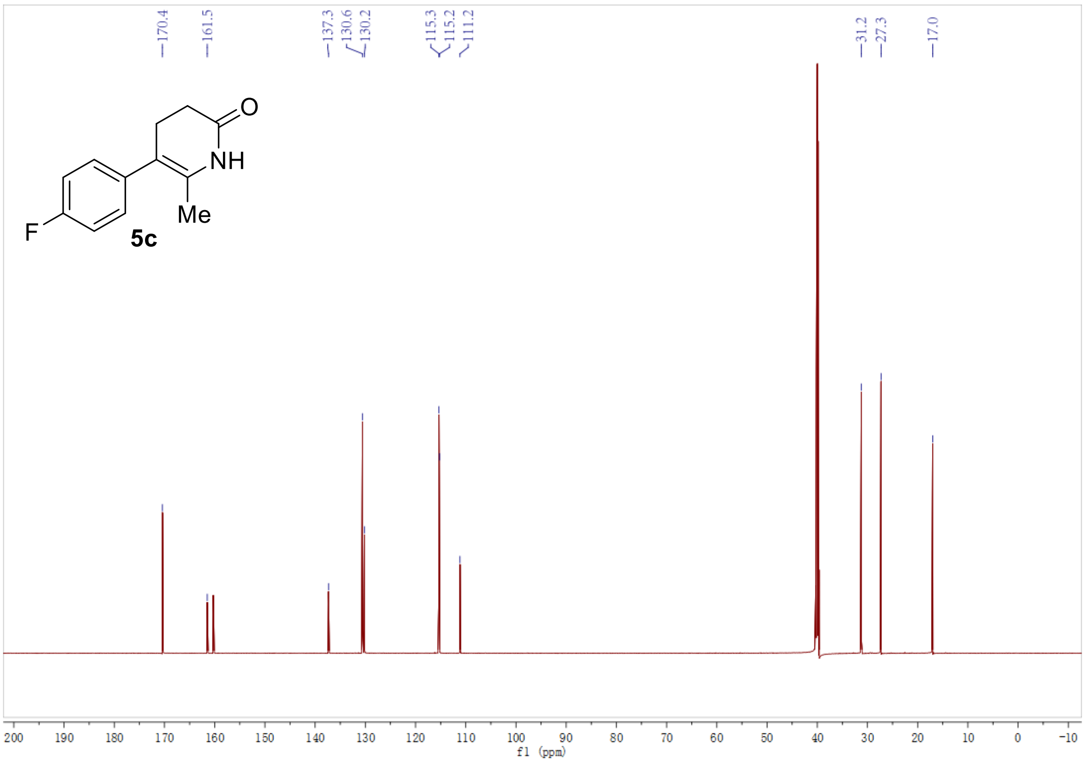

^{19}F NMR spectrum of 5-(4-fluorophenyl)-6-methyl-3,4-dihydropyridin-2(1H)-one (5c). (DMSO-d_6, 377 MHz)

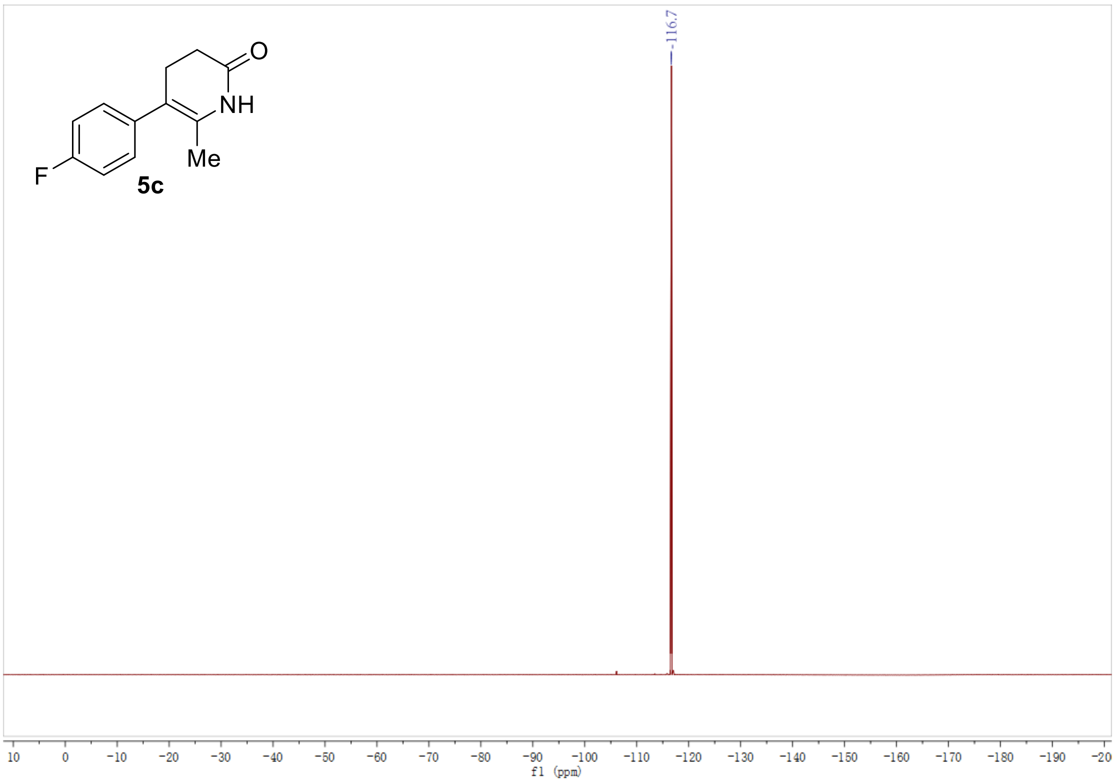

^{1}H NMR spectrum of 3-(4-aminophenyl)piperidine-2,6-dione (6). (DMSO-d_6, 800 MHz)

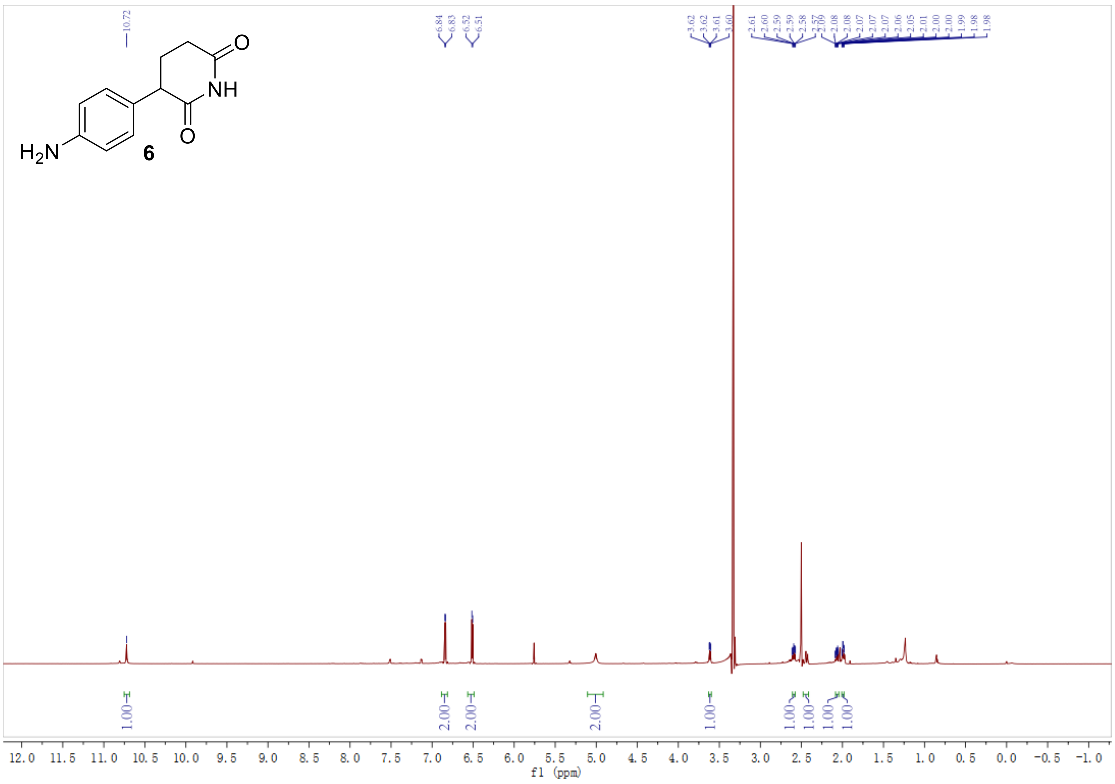

^{13}C NMR spectrum of 3-(4-aminophenyl)piperidine-2,6-dione (6). (DMSO-d_6, 200 MHz)

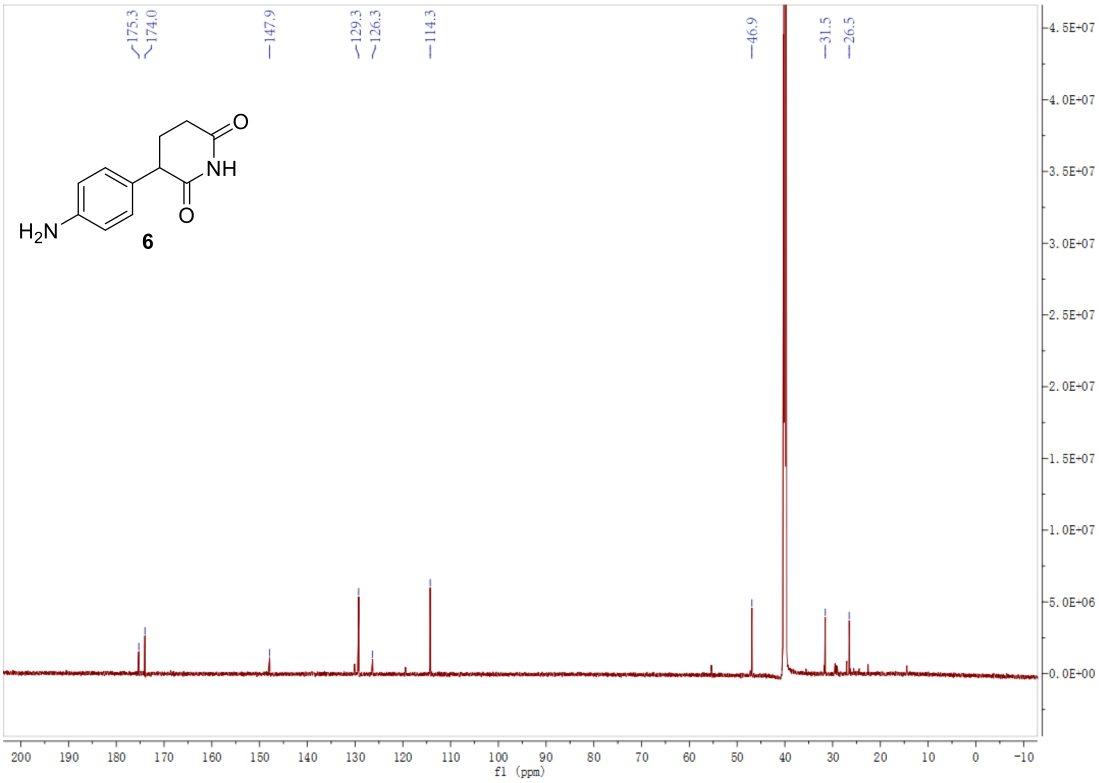

^{1}H NMR spectrum of 3-ethyl-3-(4-nitrophenyl)piperidine-2,6-dione. **(DMSO-d_6, 800 MHz)**

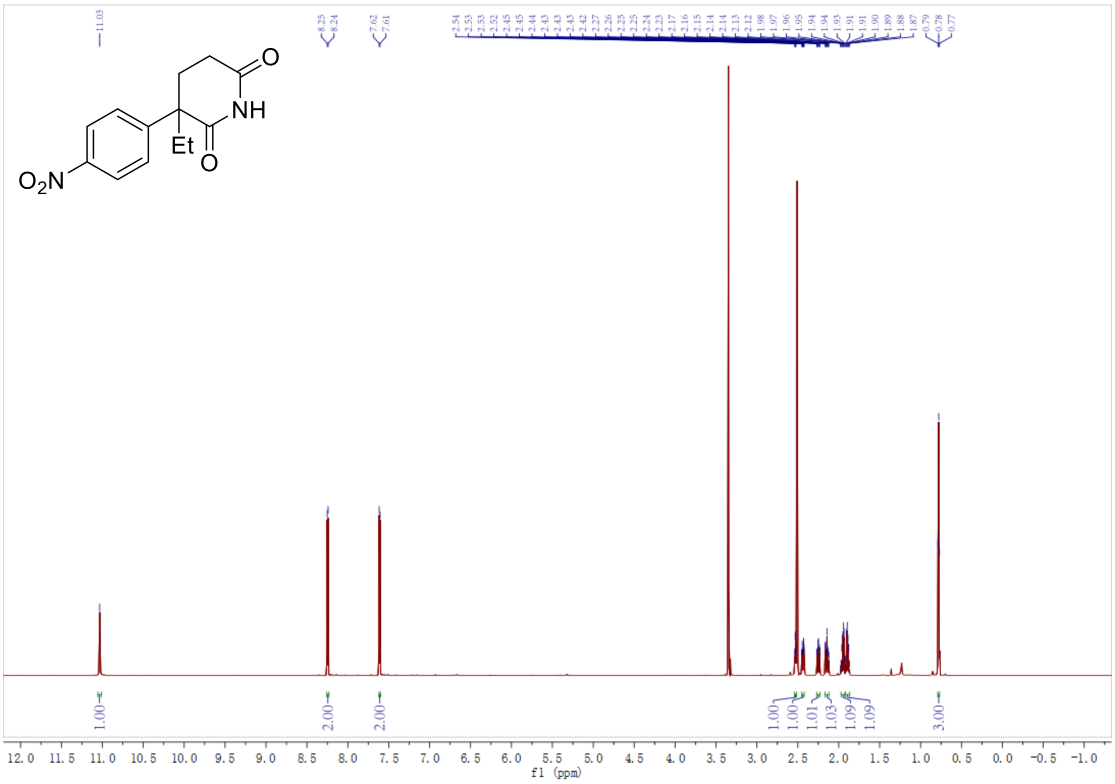

^{13}C NMR spectrum of 3-ethyl-3-(4-nitrophenyl)piperidine-2,6-dione. **(DMSO-d_6, 200 MHz)**

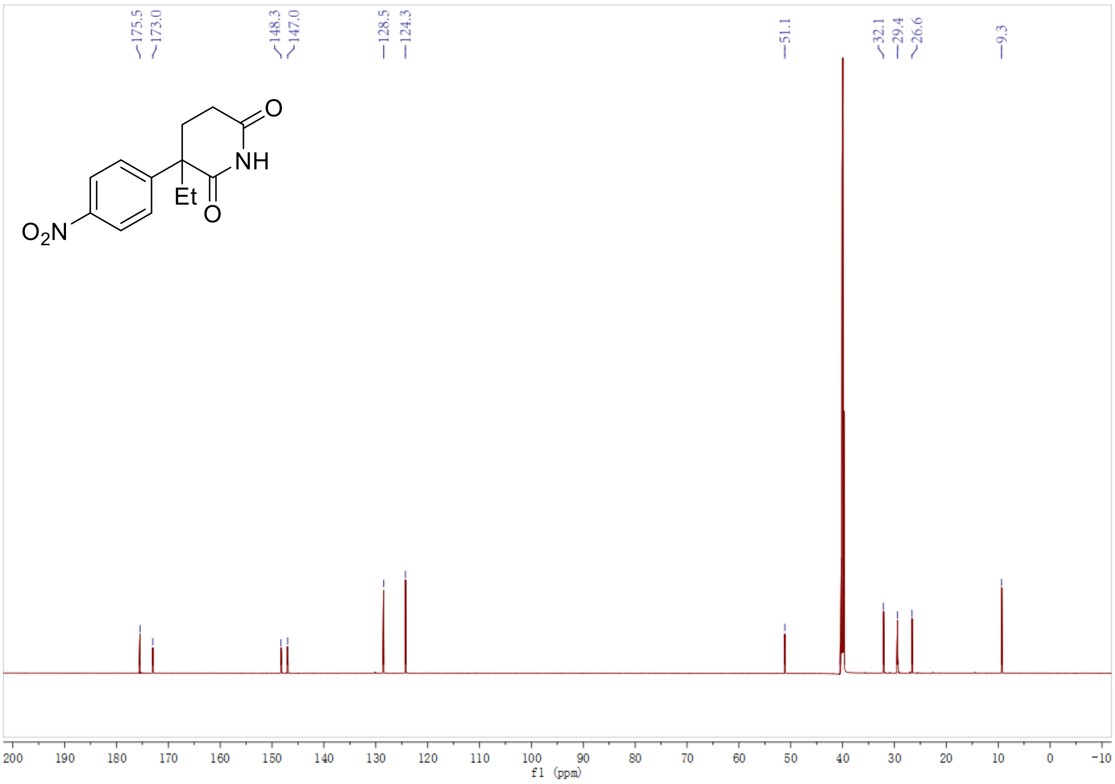

^{1}H NMR spectrum of Aminoglutethimide. (DMSO-d_6, 800 MHz)

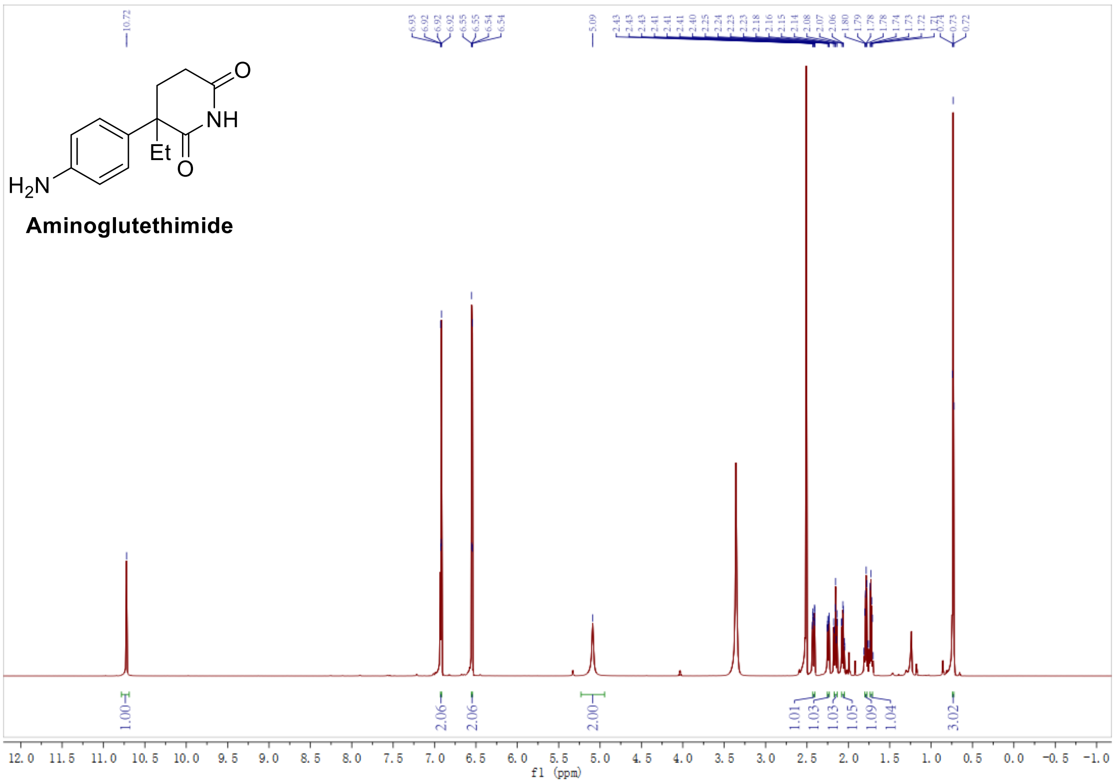

^{13}C NMR spectrum of Aminoglutethimide. (DMSO-d_6, 200 MHz)

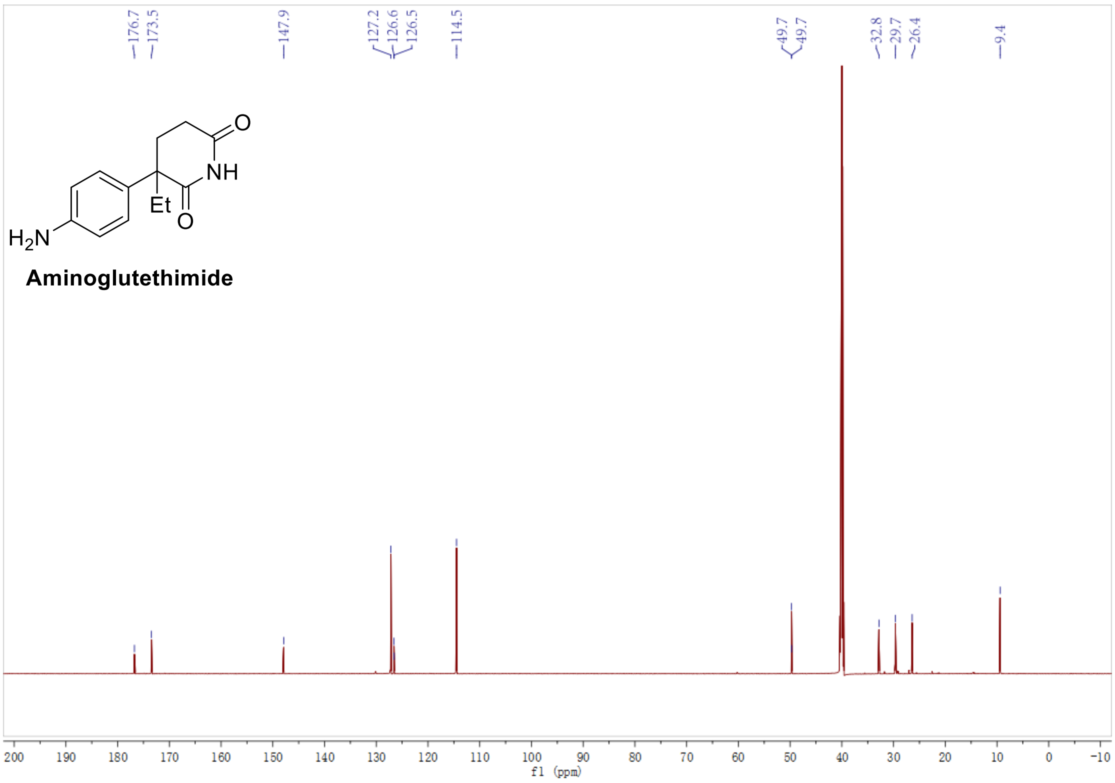

^{1}H NMR spectrum of (4-(4-fluorophenyl)piperidin-3-yl)methanol (7d). (DMSO-d_6, 500 MHz)

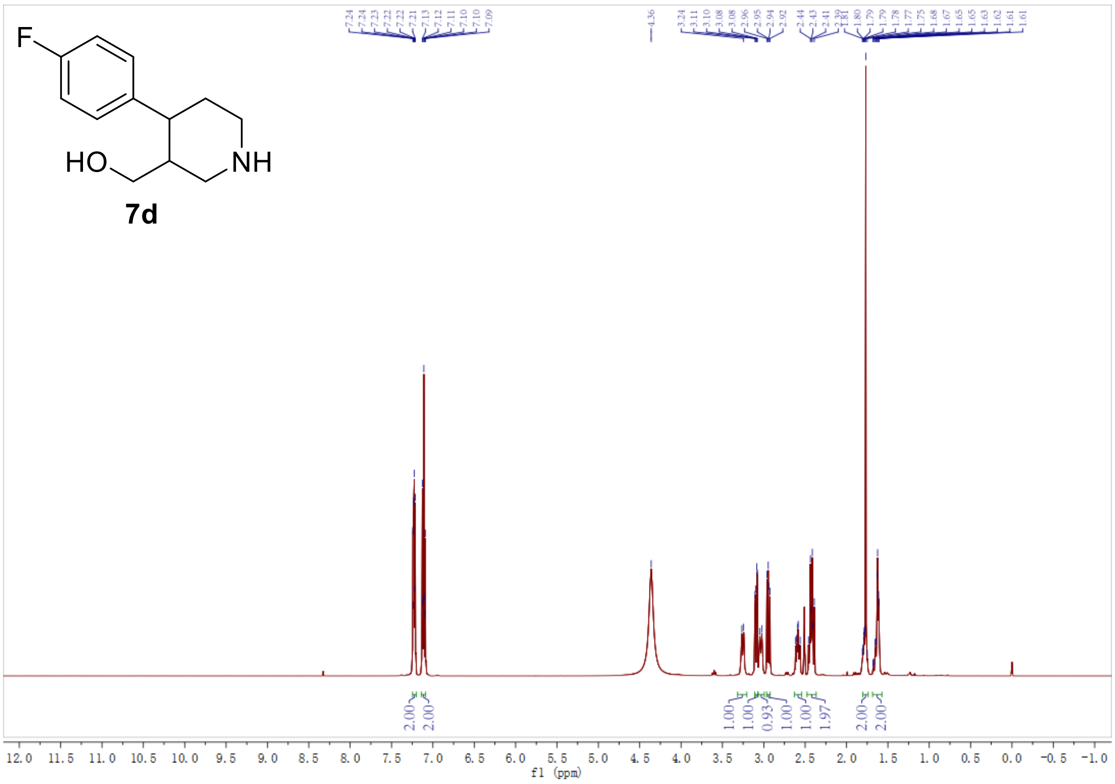

^{13}C NMR spectrum of (4-(4-fluorophenyl)piperidin-3-yl)methanol (7d). (DMSO-d_6, 125 MHz)

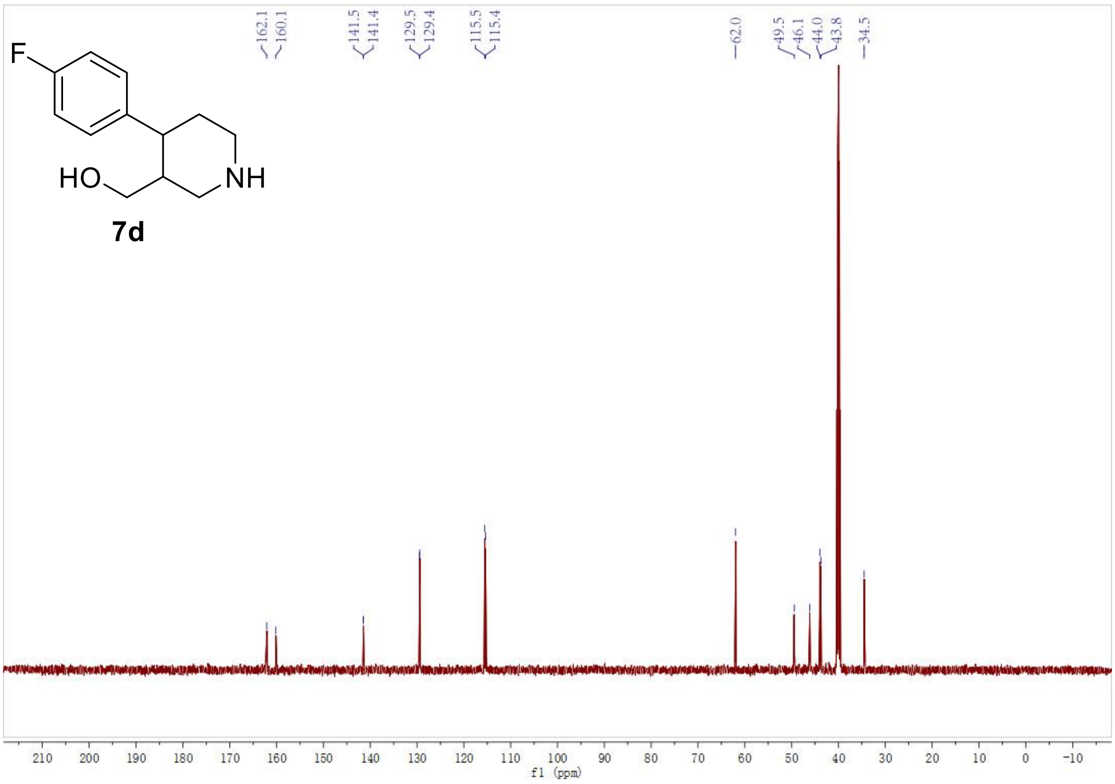

^{1}H NMR spectrum of 3-(4-bromophenyl)piperidine (8). ($CDCl_3$, 400 MHz)

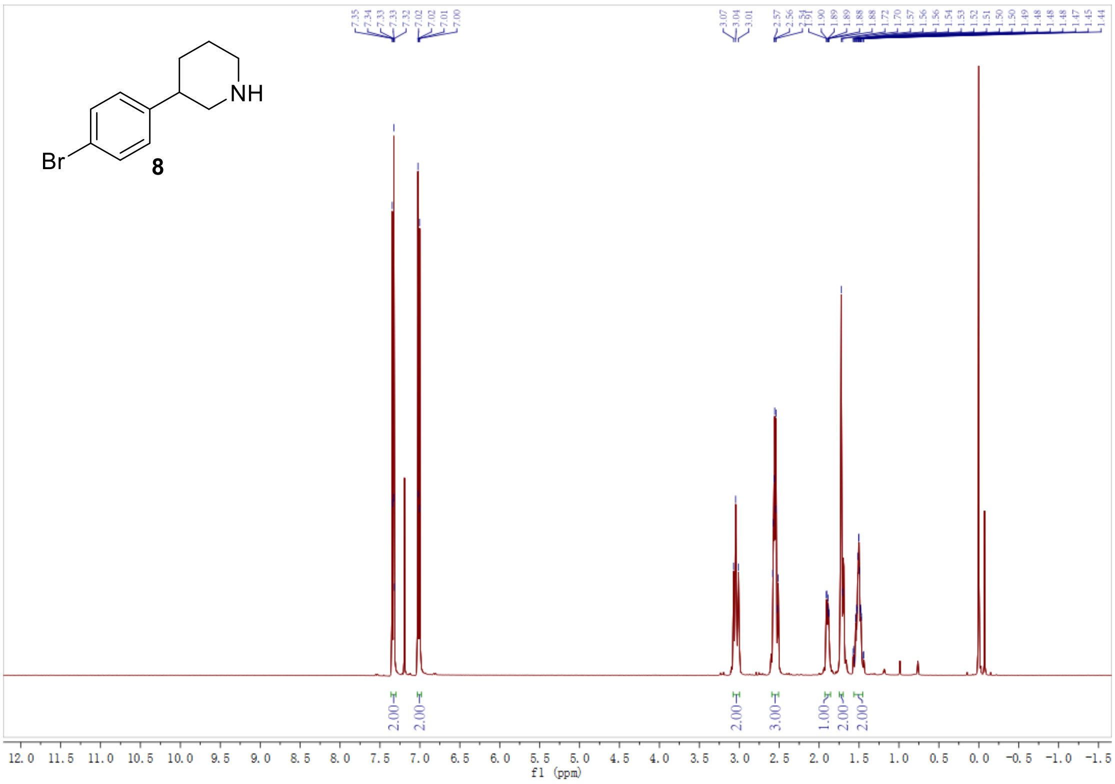

^{13}C NMR spectrum of 3-(4-bromophenyl)piperidine (8). ($CDCl_3$, 100 MHz)

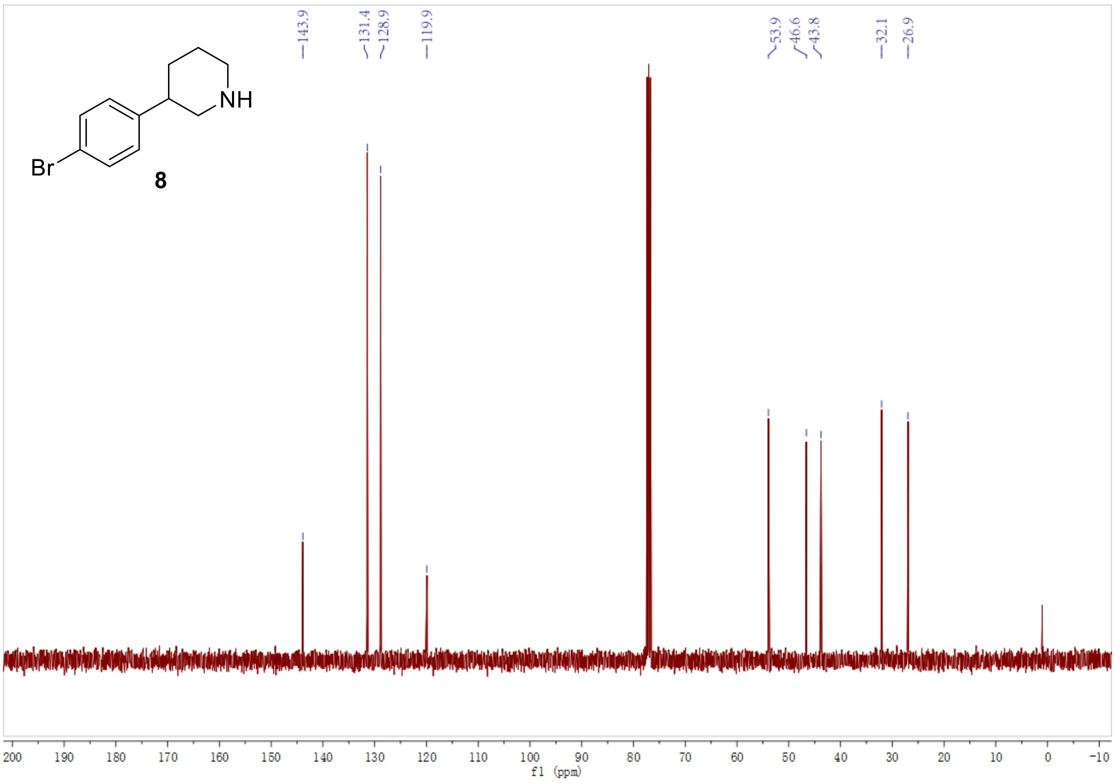

^{1}H NMR spectrum of *(S)*-*tert*-butyl 3-(4-bromophenyl)piperidine-1-carboxylate (9). (DMSO-*d*$_6$, 800 MHz)

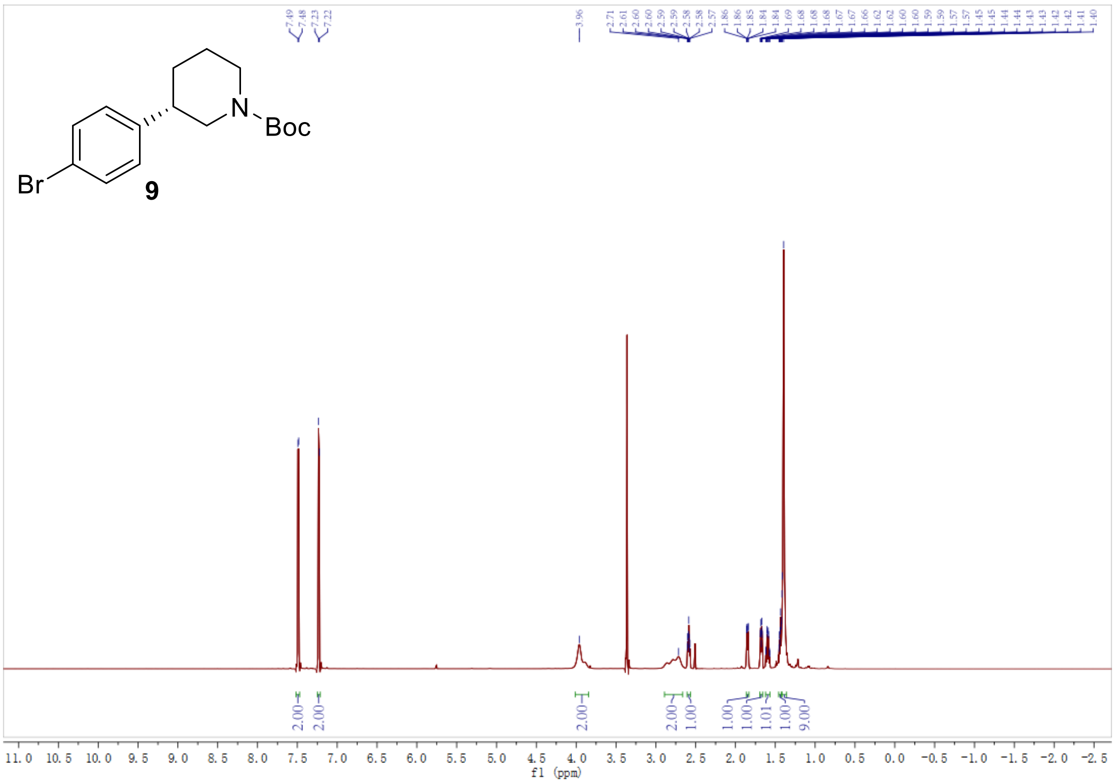

^{13}C NMR spectrum of *(S)*-*tert*-butyl 3-(4-bromophenyl)piperidine-1-carboxylate (9) . (DMSO-*d*$_6$, 200 MHz)

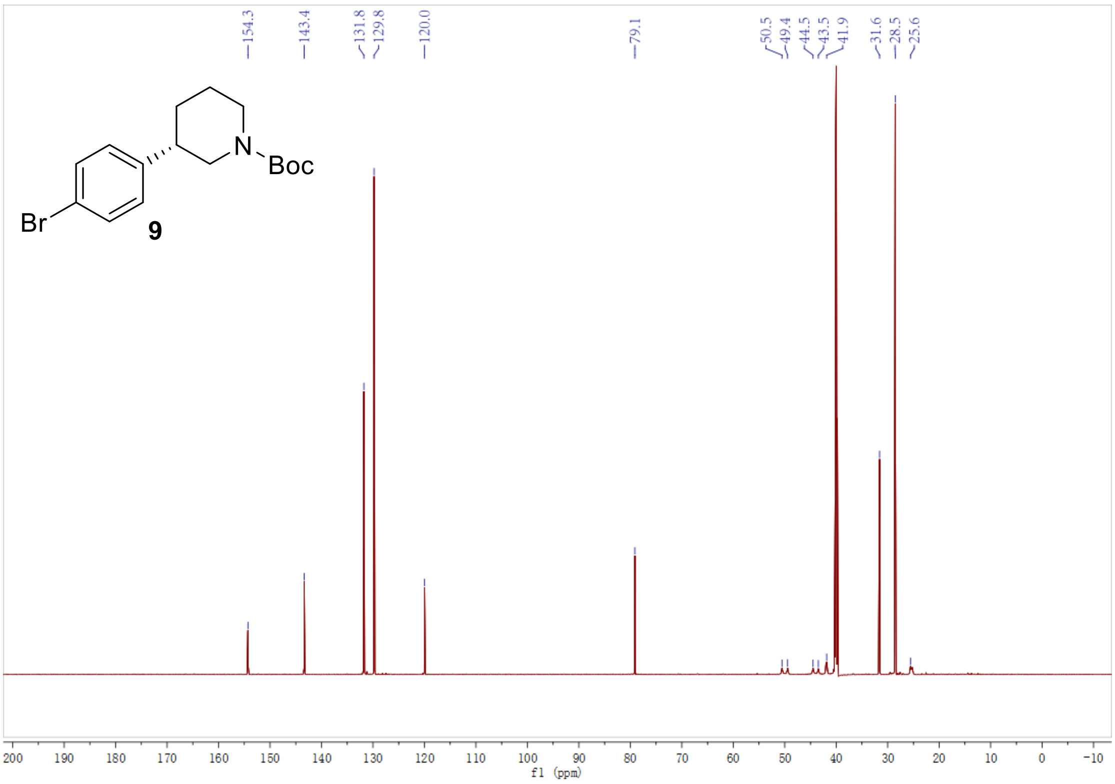

^{1}H NMR spectrum of N-(*tert*-butyl)-2*H*-indazole-7-carboxamide (11). (DMSO-d_6, 400 MHz)

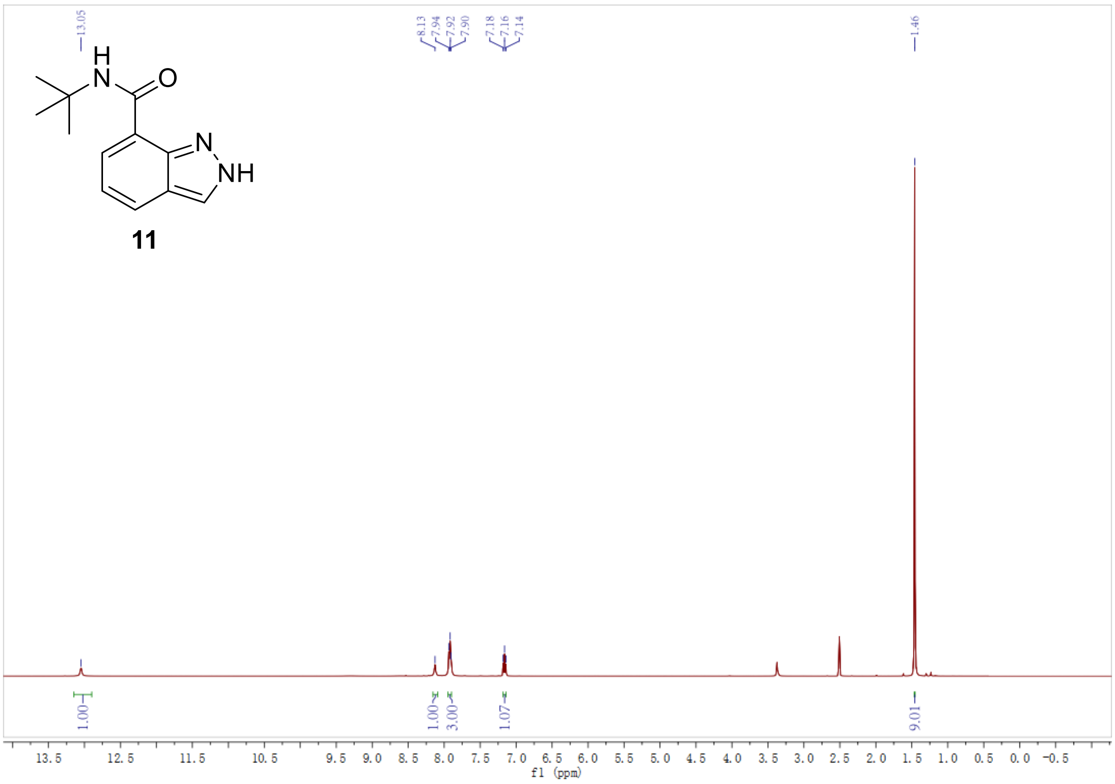

^{13}C NMR spectrum of N-(*tert*-butyl)-2*H*-indazole-7-carboxamide (11). (DMSO-d_6, 100 MHz)

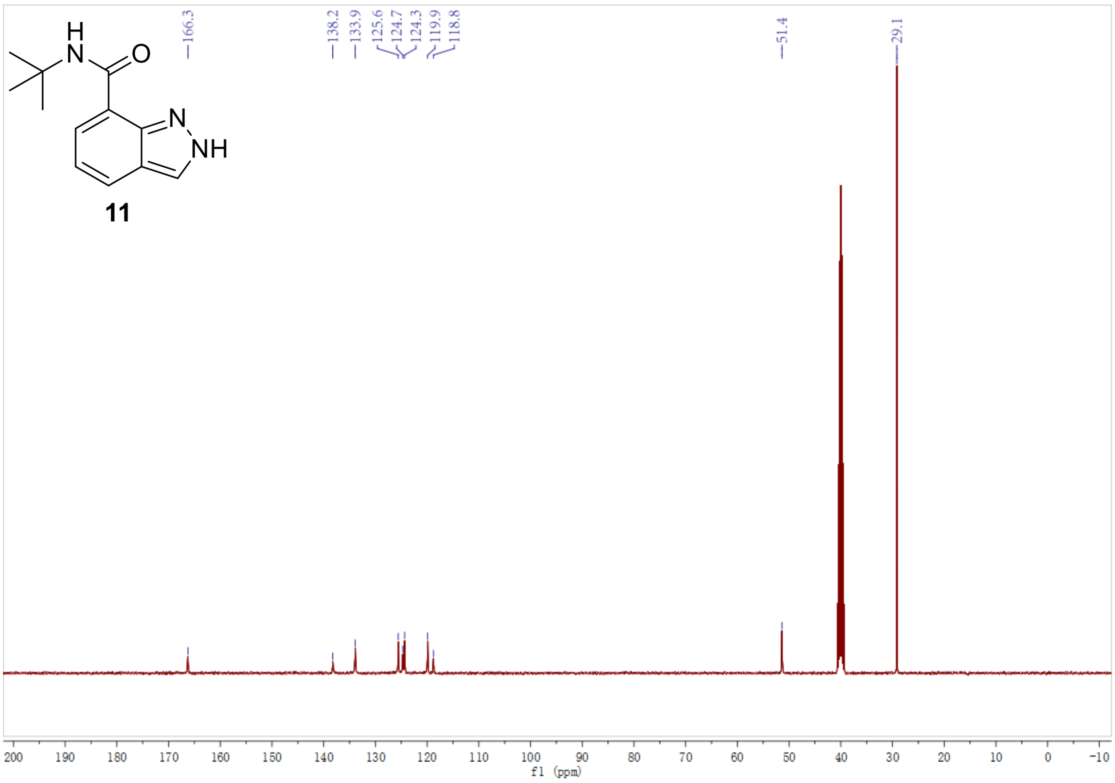

^{1}H NMR spectrum of *(S)-tert*-butyl 3-(4-(7-(*tert*-butylcarbamoyl)-2*H*-indazol-2-yl)phenyl)piperidine-1-carboxylate (12). ($CDCl_3$, 800 MHz)

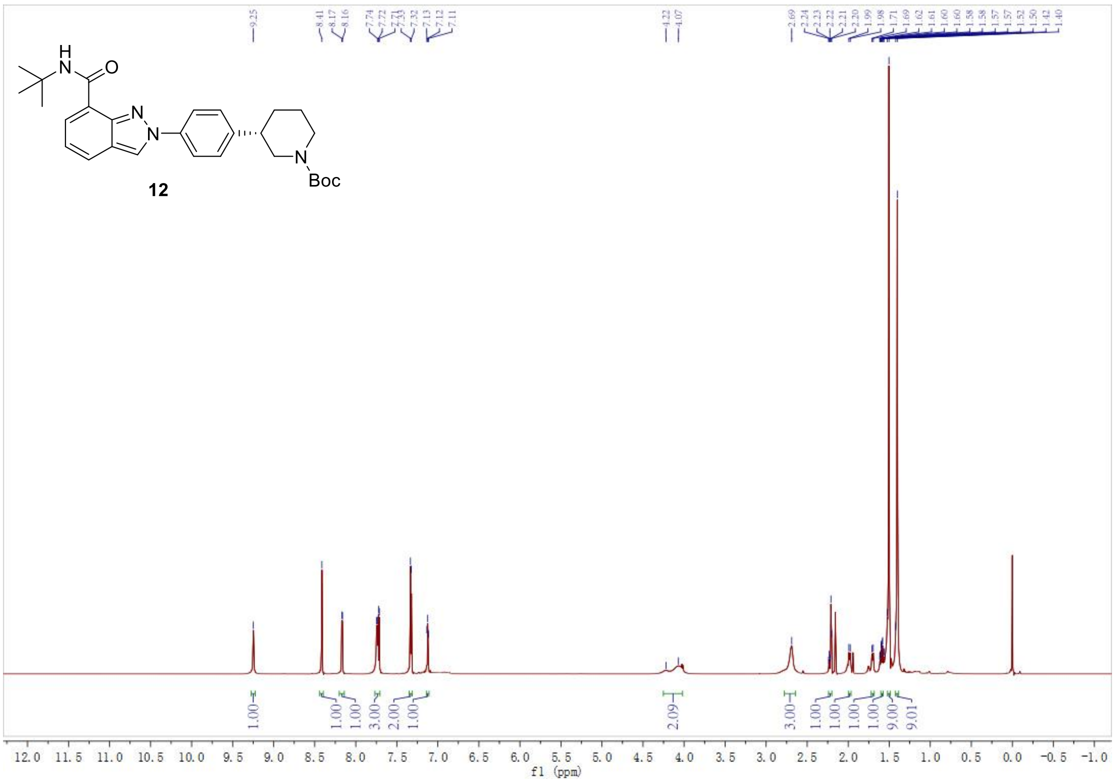

^{13}C NMR spectrum of *(S)-tert*-butyl 3-(4-(7-(*tert*-butylcarbamoyl)-2*H*-indazol-2-yl)phenyl)piperidine-1-carboxylate (12). ($CDCl_3$, 200 MHz)

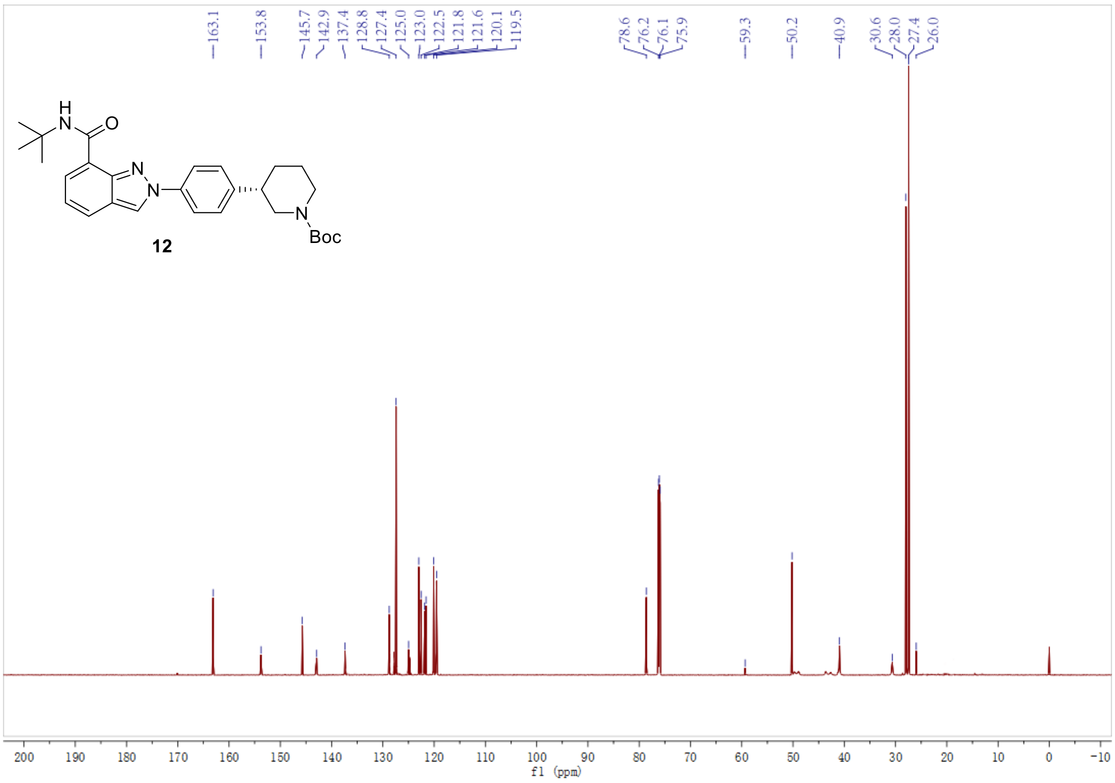

^{1}H NMR spectrum of *(S)*-2-(4-(piperidin-3-yl)phenyl)-2*H*-indazole-7-carboxamide (Niraparib). (DMSO-d_6, 400 MHz)

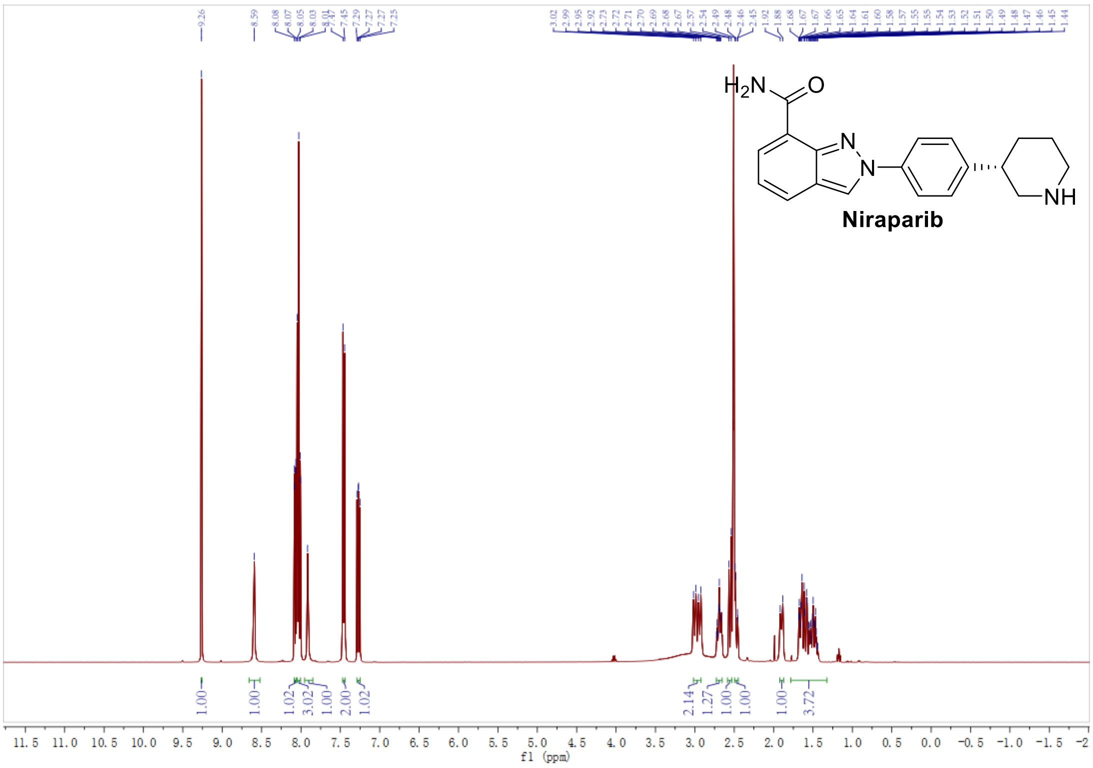

^{13}C NMR spectrum of *(S)*-2-(4-(piperidin-3-yl)phenyl)-2*H*-indazole-7-carboxamide

(Niraparib). (DMSO-d_6, 100 MHz)

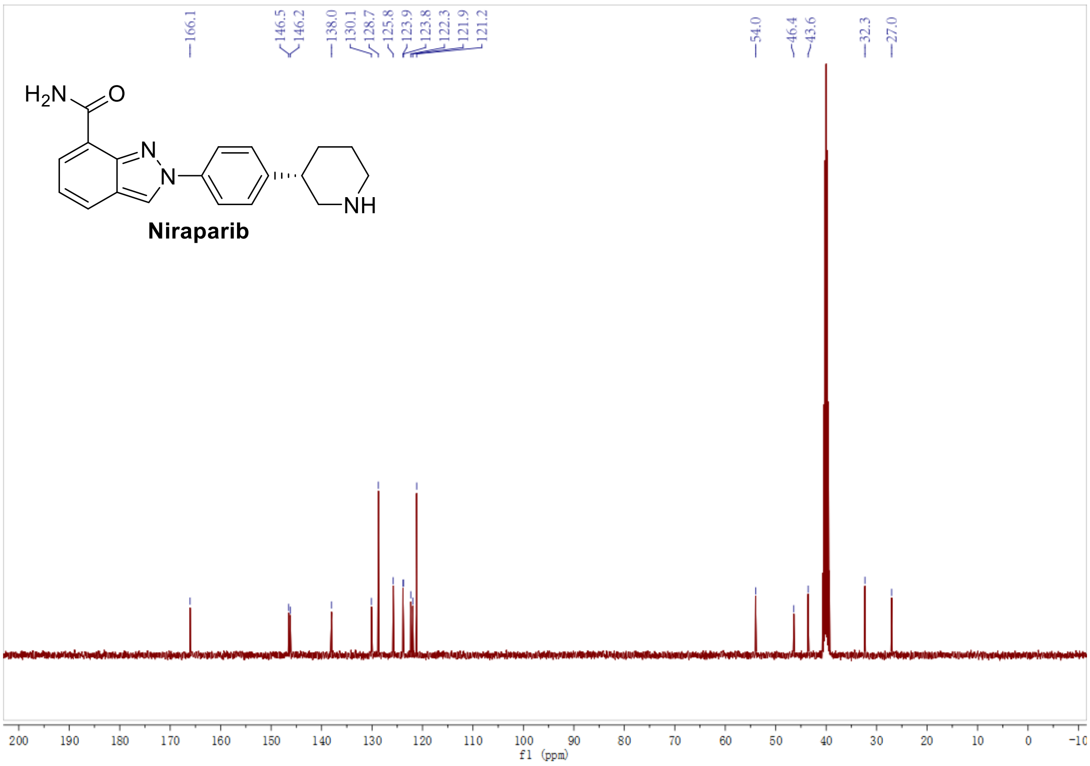

Supplement: Supplementary file 1 — Supporting Information [file OPEN-14-e202500067-s001.pdf]
